# Evolution from Bioinert to Bioresorbable: In Vivo Comparative Study of Additively Manufactured Metal Bone Scaffolds

**DOI:** 10.1002/advs.202302702

**Published:** 2023-07-10

**Authors:** Juncen Zhou, Elias Georgas, Yingchao Su, Jiayi Zhou, Nadja Kröger, Felix Benn, Alexander Kopp, Yi‐Xian Qin, Donghui Zhu

**Affiliations:** ^1^ Department of Biomedical Engineering University of Stony Brook Stony Brook NY 11794 USA; ^2^ Division of Plastic‐ Reconstructive‐ and Aesthetic Surgery University Hospital Cologne 50937 Cologne Germany; ^3^ Meotec GmbH 52068 Aachen Germany

**Keywords:** additive manufacturing, bioresorbable scaffold, bone regeneration, magnesium alloy, zinc alloy

## Abstract

Additively manufactured scaffolds offer significant potential for treating bone defects, owing to their porous, customizable architecture and functionalization capabilities. Although various biomaterials have been investigated, metals – the most successful orthopedic material – have yet to yield satisfactory results. Conventional bio‐inert metals, such as titanium (Ti) and its alloys, are widely used for fixation devices and reconstructive implants, but their non‐bioresorbable nature and the mechanical property mismatch with human bones limit their application as porous scaffolds for bone regeneration. Advancements in additive manufacturing have facilitated the use of bioresorbable metals, including magnesium (Mg), zinc (Zn), and their alloys, as porous scaffolds via Laser Powder Bed Fusion (L‐PBF) technology. This in vivo study presents a comprehensive, side‐by‐side comparative analysis of the interactions between bone regeneration and additively manufactured bio‐inert/bioresorbable metal scaffolds, as well as their therapeutic outcomes. The research offers an in‐depth understanding of the metal scaffold‐assisted bone healing process, illustrating that Mg and Zn scaffolds contribute to the bone healing process in distinct ways, but ultimately deliver superior therapeutic outcomes compared to Ti scaffolds. These findings suggest that bioresorbable metal scaffolds hold considerable promise for the clinical treatment of bone defects in the near future.

## Graphical abstract

In vivo performance of magnesium (Mg), zinc (Zn), and titanium (Ti) additively manufactured (laser powder bed fusion) scaffolds is compared using a rabbit femur defect model to tackle bone regeneration challenges. The authors evaluate biodegradation, bone regeneration, biomechanics, and biological responses during implantation, aiming to elucidate the distinctions between bioinert and bioresorbable scaffolds and provide a comprehensive understanding of the interplay between bone healing and metal scaffolds.

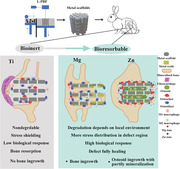

## 1 Introduction

Bone defects, arising from trauma, infections, or surgical procedures such as tumor resection and craniotomy, continue to present clinical challenges for regeneration. Although bone grafts are a viable treatment option, their clinical application is constrained by surgical risks and donor site morbidity associated with autologous grafts, as well as source limitations and alloimmunity risks in the case of allogeneic grafts.^[^ ^1^, ^2^ ^]^

Additive manufacturing of scaffolds, with its customizable design and fabrication capabilities, has demonstrated immense potential in the treatment of bone defects. A wide range of scaffolding materials has been investigated, including ceramics, polymers, metals, and composite materials.^[^ ^3^, ^4^ ^]^ While ceramic or polymer scaffolds may be suitable for low‐stress loading bone defect sites, metal scaffolds provide the necessary mechanical support and stability for high load‐bearing scenarios, such as long bone defects.^[^ ^5^ ^]^ Among the various additive manufacturing methods, laser powder bed fusion (L‐PBF) has proven to be the most successful approach for creating bioinert metal implants, such as titanium (Ti) and its alloys,^[^ ^6^, ^7^ ^]^ stainless steel,^[^ ^8^ ^]^ tantalum (Ta),^[^ ^9^ ^]^ and cobalt‐chrome (CoCr) alloys.^[^ ^10^ ^]^ L‐PBF can achieve high dimensional accuracy and near‐complete densification of metal parts, ensuring that the resulting metal scaffolds meet clinical requirement.^[^ ^11^ ^]^

Ti and its alloys have become the predominant materials for metal orthopedic implants, attributed to their exceptional mechanical properties and corrosion resistance. Consequently, research has delved into the potential of additively manufactured Ti scaffolds for bone regeneration applications.^[^ ^12^, ^13^, ^14^, ^15^ ^]^ The porous structure obtained through additive manufacturing techniques lowers the elastic moduli of Ti‐based materials, and numerous clinical trials have showcased the successful use of these scaffolds in calvaria and tibia defects.^[^ ^16^, ^17^, ^18^, ^19^ ^]^ Despite these promising preclinical findings, Ti‐based scaffolds possess non‐biodegradable and bio‐inert properties, causing their material, structure, and biomechanics to remain largely unaltered long after implantation. This may result in obstruction at the bone defect site and impede the intricate healing process.^[^ ^20^ ^]^ These constraints of Ti‐based scaffolds emphasize the necessity for developing the next generation of metal‐based scaffolds specifically aimed at bone regeneration.

Long bone defect healing is a dynamic, multistage process influenced by factors such as immune response, mechanical loading, osteogenesis, and angiogenesis.^[^ ^21^, ^22^, ^23^ ^]^ Implants impact these factors, with healing outcomes hinging on the dynamic interplay between scaffolds and the regeneration process.^[^ ^24^, ^25^ ^]^ Bioresorbable materials, more adaptable to bone regeneration dynamics than bio‐inert ones, offer a favorable healing environment, ultimately leading to improved therapeutic outcomes.^[^ ^26^, ^27^ ^]^

Bioresorbable metals, such as magnesium (Mg), zinc (Zn), and their alloys, gradually degrade over time after implantation. This not only minimizes the risk of obstruction but also releases degradation products that may have positive effects on tissue regeneration.^[^ ^28^, ^29^, ^30^, ^31^ ^]^ Both Mg and Zn ions have been proven to be beneficial for bone regeneration, as they can enhance osteogenesis and angiogenesis.^[^ ^32^, ^33^, ^34^ ^]^ Additionally, Zn ions are believed to suppress osteoclastogenesis and osteoclast resorption activities.^[^ ^35^, ^36^ ^]^ Thus, Mg, Zn, and their alloys are promising candidates for next‐generation additively manufactured scaffolds.

In 2015, L‐PBF was employed for the first time to fabricate a porous scaffold made of Mg alloy AZ91.^[^ ^37^ ^]^ Since then, other Mg‐based alloys, such as WE43 Alloys.^[^ ^38^ ^]^ and Mg‐Ca alloys,^[^ ^39^ ^]^ have also been explored as scaffolding materials, with detailed structural analyses conducted.^[^ ^40^ ^]^ In vitro studies investigating degradation behavior and biocompatibility have shown promising results for both porous scaffolds and bulk Mg samples fabricated by L‐PBF.^[^ ^11^, ^41^ ^]^ Zn‐based metals, processed more recently by L‐PBF, saw the first report on porous scaffolds composed of pure Zn and Zn‐xWE43 alloys in 2019.^[^ ^42^, ^43^ ^]^ Another alloy type, Zn‐Mg alloys, has been used as printing materials for scaffolds, exhibiting decent mechanical strength and accelerated degradation rates.^[^ ^44^, ^45^ ^]^ Studies have also demonstrated the good cytocompatibility of Zn‐based scaffolds with bone‐related cells.^[^ ^44^, ^45^, ^46^ ^]^ and one study reported enhanced bone formation around Zn‐1Mg porous scaffolds compared to pure Zn counterparts.^[^ ^45^ ^]^ However, despite these promising findings, an in vivo comparative study of bioinert and bioresorbable additively manufactured scaffolds for bone regeneration is lacking,^[^ ^11^, ^41^ ^]^ leading to an absence of comprehensive and detailed analysis of the bone regeneration process surrounding and within these metal scaffolds. Addressing this knowledge gap is crucial for optimizing scaffold design and enhancing clinical outcomes.

In this study, we conducted a side‐by‐side comparative analysis using a rabbit femur defect model to evaluate the in vivo performance of Mg, Zn, and Ti scaffolds. A sham group without implant intervention was included as a benchmark. Ti scaffolds were fabricated using an industrial‐scale L‐PBF machine, while Mg and Zn scaffolds were printed with a laboratory‐scale L‐PBF machine. To thoroughly compare bioinert and bioresorbable scaffolds and their impact on the bone healing process, we performed in vivo assessments covering various aspects of bone healing, including biodegradation behavior, bone regeneration, biomechanical analysis, and an array of biological responses during both early and late stages of implantation. These assessments targeted different scaffold regions exposed to a variety of physiological environments. Through this research, our goal is to provide an overview of the benefits and drawbacks of additively manufactured metal scaffolds and an extensive, in‐depth understanding of the interactions between the bone defect healing process and metal scaffolds.

## 2 Results

### 2.1 Scaffold Fabrication and In Vivo Study in the Rabbit Femur Model

**Figure 1:**
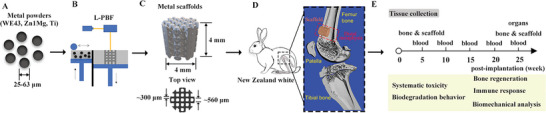
Study flowchart. A) Micro‐scale powders for additive manufacturing: Mg scaffold (WE43 alloy), Zn scaffold (Zn1Mg alloy), and Ti scaffold (Grade 1 pure Ti). B) Mg and Zn scaffolds are produced using a laboratory‐scale L‐PBF machine, while the Ti scaffold is fabricated with an industry‐scale L‐PBF machine. C) All scaffolds undergo processing with identical design parameters. D) New Zealand white rabbits are selected for in vivo animal studies, and a 4 mm diameter circular defect is created on the distal femur to accommodate the scaffold insertion. E) Timeline for procedures such as blood, organ, and tissue collection, as well as a list of subsequent analyses.

This study aimed to investigate the interaction between bone regeneration and bio‐inert/biodegradable metal scaffolds and the therapeutic outcomes. Pure Ti (Grade 1) was selected as the bio‐inert material, while WE43 and Zn1Mg alloys were chosen as representative biodegradable metals due to their superior degradation behavior and biocompatibility among Mg and Zn alloys. To achieve a complex structure, L‐PBF was employed as the additive manufacturing method (**Figure** **1**). All scaffolds shared the same blueprint for additive manufacturing, a cylindrical shape and overall size (4 mm in both diameter and height), allowing them to fit the defect model. A pore thickness of ≈560 µm ensured sufficient interspaces for new bone ingrowth. Despite using the same blueprint, the actual size of strut thickness, pore thickness, and pore area varied across scaffold types due to the influence of the material on the additive manufacturing process (Figure S1, Supporting Information). The scaffolds' microstructures also exhibited differences (Figure S2, Supporting Information), with significantly higher amounts of cavities found inside the Zn scaffold compared to the other two. Binary Zn alloy powders were more sensitive to additive manufacturing conditions, and these cavities likely originated from gas pores due to the high energy density during the additive manufacturing process. The Mg and Ti scaffolds exhibited smooth surfaces, while the Zn scaffold demonstrated a granular surface.

In this study, no fixation device was used to eliminate any potential impact on biodegradation behavior and biological response. Scaffold implantation was conducted through a push‐in approach. A circular 4 mm diameter defect (0.4–0.6 times the diameter of the affected bone) was created in the distal metaphysis of the right femur of female New Zealand White rabbits. This region has relatively high osteogenic activity and bears significant load, making it suitable for metal scaffolds to serve as a tissue engineering matrix for bone defects. The defect site and size ensured adequate bone regeneration activity to investigate interactions with bio‐inert/biodegradable scaffolds.

Blood chemistry panels and histological analyses of the three scaffold types at week 25 post‐implantation revealed no significant differences. Hematological indices and histological details of various organs indicated no systemic toxicity in all groups (Figure S3 and Table S1, Supporting Information). Detailed evaluations of biodegradable behavior, bone regeneration, immune response, and biomechanical analysis focused on tissues and scaffolds collected at weeks 5 and 25.

### 2.2 Biodegradation Trends of Mg and Zn Scaffolds Depend on the Localized Environment

Both Mg and Zn scaffolds exhibited distinct biodegradation trends in different regions exposed to varying physiological environments. The cross‐section of the scaffold in the longitudinal plane revealed that more degradation products formed in the scaffold region exposed to the bone marrow cavity than to the bone tissue.

**Figure 2:**
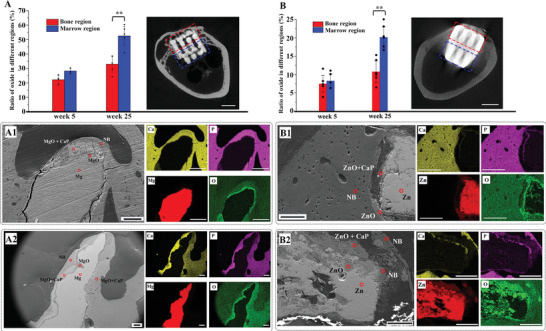
A) Mg and B) Zn scaffolds degradation behavior. (A) The ratio of oxides in the bone and marrow regions of Mg scaffolds (longitudinal plane of scaffold) derived from Micro‐CT, with an inset Micro‐CT image of the 25‐week implanted Mg scaffold illustrating bone and marrow regions. SEM images and EDX mapping of Mg‐25w scaffold in the bone region (A1) and marrow region (A2). (B) The volume ratio of Zn oxides in bone and marrow regions derived from SEM images and EDS/Elemental mapping, with an inset Micro‐CT image of the 25‐week implanted Zn scaffold illustrating bone and marrow regions. SEM images and EDS/Elemental mapping of the 25‐week implanted Zn scaffold in the bone region (B1) and marrow region (B2). (A,B) white scale bar: 2 mm, (A1‐2, B1‐2) Black scale bar: 100 µm, White scale bar: 200 µm. NB: new bone, Mg: Mg metal, MgO: Mg oxides, Zn: Zn metal, ZnO: Zn oxides, CaP: calcium phosphate composites. Data are presented as mean ± SEM. *P < 0.05, **P < 0.01, ***P < 0.001, ****P < 0.0001 (one‐way ANOVA).

The volume ratio of oxides in the Mg scaffold increased from 26% in week 5 to 41% in week 25, while the total volume, including Mg metal and oxides, exhibited a negligible decrease (Figure S4A, Supporting Information). X‐ray model images showed that the distribution of oxides on the Mg scaffold was inhomogeneous after 25 weeks of implantation (Figure S4B, Supporting Information). The majority of oxides were located in the central region of the scaffold exposed to the bone marrow cavity. The 5‐week implantation did not significantly alter the regional distribution of oxides, but by week 25, the volume ratio of oxides reached 51% in the marrow region of the Mg scaffold and only 33% in the bone region (**Figure** **2**A). In the bone region, the degradation product layer on the Mg scaffold was thin in week 5 and separated into two layers, with the inner layer (close to the Mg metal substrate) composed of Mg and (Oxygen) O elements, and the outer layer composed of Mg, O, Calcium (Ca), and Phosphrus (P) elements (Figure S4E1, Supporting Information). The thin inner layer was also observed in week 25, while the outer layer was rich in Ca and P elements, and seamless integration occurred between the outer layer and newly formed bone (Figure 2A1). On the other end of the scaffold in the marrow region, the degradation behavior was accelerated and rather

inhomogeneous distributed. In week 5, the conversion of the Mg metal substrate to oxides occurred on all surfaces, with significant portions of oxides rich in Ca and P elements (Figure S4E2, Supporting Information). By week 25, the majority of the Mg scaffold in the central region had converted to Ca/P‐rich oxides, and layers of new bone and oxides could be observed on the scaffold surface in the peripheral region (Figure 2A2). Figure S3 (Supporting Information)(C&D) displayed the distribution of oxides in different regions.

Due to the high density of Zn metal, beam hardening, scatter effects, and splay artifacts occurred in the Micro‐CT scan.^[^ ^47^ ^]^ Therefore, this study relied on scanning electron microscopy/energy dispersive X‐ray spectroscopy (SEM/EDS) images to quantify oxides in Zn scaffolds for better accuracy. The volume ratio of oxides in the bone and marrow regions of Zn scaffolds was approximately 7.5% after 5 weeks, which increased to 10% and 20%, respectively, by week 25 (Figure 2B). This suggests an inhomogeneous degradation progress in different regions of Zn scaffolds exposed to varying physiological environments, similar to that observed in Mg scaffolds. Although the surface of the Zn scaffold was granular and porous, the surface oxide layer was thin and scattered in both bone and marrow regions in week 5 (Figure S5A1,2, Supporting Information). Oxides were also observed in cavities, with Ca/P elements distributed separately. Unlike the two‐layer structure observed in Mg scaffolds, the oxide layer on Zn scaffolds in the bone region remained thin but was rich in Ca/P. In contrast, a significant portion of the scaffold in the marrow region had converted to oxide, accompanied by a Ca/P‐rich outer layer (Figure 2B1,2). The distribution of oxides in Zn scaffolds was much more scattered compared to that in Mg scaffolds, as highlighted in Figure S5 (Supporting Information)(B1–C2).

As anticipated, the oxide layer on the Ti scaffold was thin, and only the P element could be detected, regardless of location and implantation time (Figure S6, Supporting Information).

### 2.3 Bone Regeneration and Integration in Different Regions of Scaffolds

**Figure 3:**
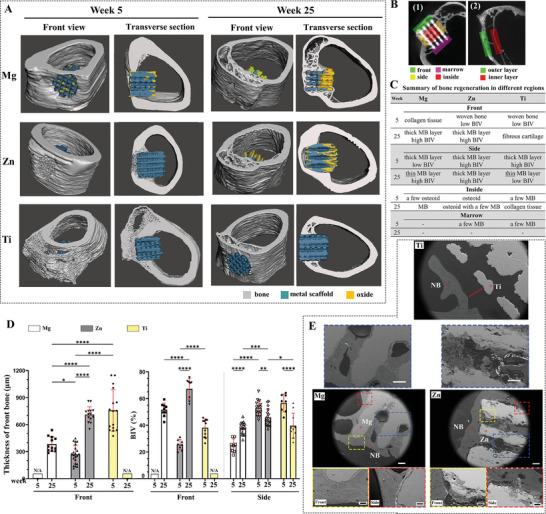
Bone regeneration. A) 3D models derived from representative Micro‐CT scans incorporating bone tissue and the scaffold. Six models: Mg scaffold at 5‐ and 25‐weeks post‐implantation, Zn scaffold at 5‐ and 25‐weeks post‐implantation, and Ti at 5‐ and 25‐weeks post‐implantation. Two angles of view: front view (left) and transverse section (right). B) Illustration of regions of interest for bone tissue assessment in 1) scaffold groups and 2) sham groups. C) Summary of bone regeneration in different regions, including front, side, inside, and marrow regions. MB: mineralized bone, BIV: bone‐to‐implant volume. D) The thickness of newly formed bone in the front region and bone‐implant volume (BIV) in front and side regions within a 1 mm range, derived from histochemistry staining images (>10). Data are presented as mean ± SEM. *P < 0.05, **P < 0.01, ***P < 0.001, ****P < 0.0001 (one‐way ANOVA). E) SEM images demonstrating osseointegration of Mg, Zn, and Ti scaffolds after 25 weeks of implantation. Yellow dotted boxes show detailed bone‐scaffold interfaces in the front regions, red ones present those in the side region, and blue ones demonstrate the tissue condition in the inside regions. Red arrows indicate gaps between bone tissue and Ti scaffold. E) White scale bar: 200 µm, black scale bar: 50 µm.

The overall structure of scaffolds and adjacent bone tissue was depicted through the reconstructed Micro‐CT scan results (**Figure** **3**A) and concisely summarized according to various regions (Figure 3B‐D). Representative transverse section images obtained from the Micro‐CT scans are presented in Figure S7 (Supporting Information).

In week 5, there was no continuous bone layer formed in the front region of the Mg scaffold, and the bone‐to‐implant volume (BIV) in the side region was limited, resulting in a relatively weak integration between the Mg scaffold and surrounding bone tissue. However, by week 25, the front region of the Mg scaffold was fully enveloped by a mineralized bone layer, 380 µm thick, with a BIV of 50%. Additionally, the BIV in the side region increased to 38%. This led to the successful integration of bone tissue with the front and side regions of the Mg scaffold during the late stage. The detailed interface condition between the bone and Mg scaffolds further confirmed the effective osseointegration of Mg scaffolds (Figure 3E). The ingrowth of bone tissue from the front region was observable, and the penetration of new bone tissue from the side region was also evident. Micro‐CT scan results revealed that the bone volume fraction (BV/TV) of bone tissue inside the Mg scaffold reached 0.23 ± 0.03, and the distribution of newly formed bone within Mg scaffolds favored the bone region (57%) in the transverse plane and the peripheral region (71%) in the frontal plane (Figure S8, Supporting Information), areas that were closer to the native bone tissue.

**Figure 4:**
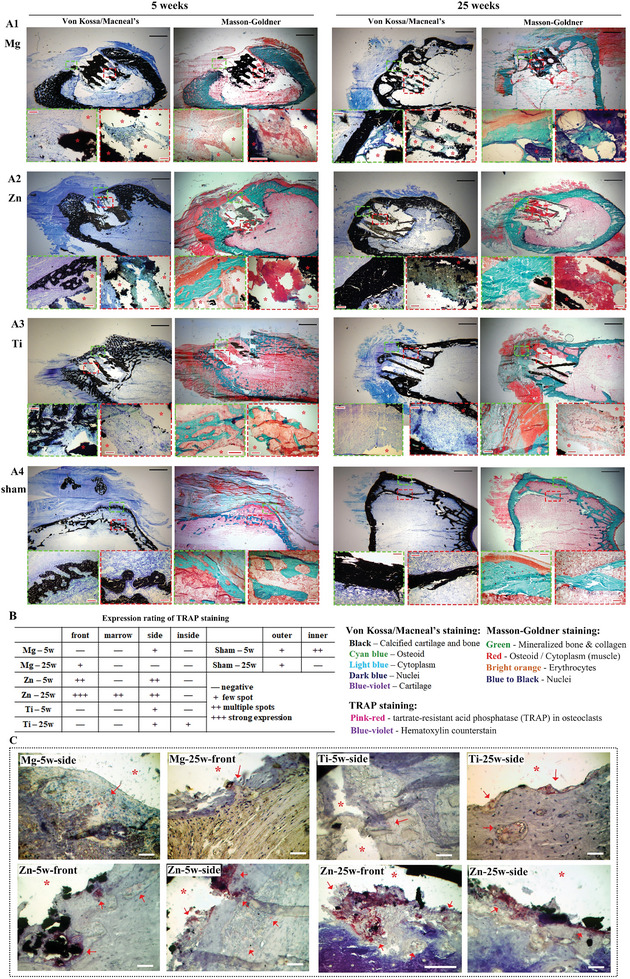
Histochemical staining. Von Kossa/Macneal's staining and Masson‐Goldner staining of A1) Mg scaffold, A2) Zn scaffold, A3) Ti scaffold, and A4) sham control after 5 and 25 weeks of implantation. Green boxes show detailed histological structures in front of the scaffold, while red boxes show structures inside the scaffold. Black scale bar: 2 mm, red scale bar: 200 µm. B) Summary of expression ratings for TRAP staining in all samples. C) Representative TRAP staining images of front and side regions of scaffolds. * indicates the location of the scaffold, → indicates spots with positive TRAP staining. White scale bar: 100 µm.

For the Zn scaffolds, the front region was already covered by a porous, mineralized bone layer in week 5, and the side region exhibited a high BIV (53%). By week 25, the porous front bone had transformed into a compact bone layer, 710 µm thick, with a BIV of 67%, signifying enhanced bone quality in this area. Although the side region had a comparable BIV (46%), the total bone mass surrounding the Zn scaffold was greater than that around the Mg scaffold. However, the osseointegration of the Zn scaffold was not as effective as the Mg scaffold, as small gaps were observed at the Zn scaffold‐bone interface (Figure 3E). The tissue inside the Zn scaffold was not visible through Micro‐CT scans; however, SEM and histological staining images (Figure 3E and Figure 4A2) revealed that osteoid tissue had penetrated the Zn scaffold by week 5. By week 25, the inside region of the scaffold was filled with osteoid tissue, but only a small portion had calcified into mineralized bone, which was distributed sporadically.

Ti scaffolds exhibited a contrasting bone regeneration trend compared to Mg scaffolds. In week 5, the front region was covered by a porous bone layer with a substantial thickness of 758 µm and a BIV of 38%, but by week 25, no bone layer was visible. The BIV in the side region decreased from 56% in week 5 to 39% in week 25. A noticeable decline in bone tissue occurred after week 5 surrounding the Ti scaffolds, resulting in a sizable gap between the bone tissue and Ti scaffolds, which indicated poor osseointegration. New bone formation in the marrow region exhibited similar patterns for all three types of scaffolds. A minimal amount of bone formed in the marrow regions of Zn and Ti scaffolds in week 5, and by week 25, no bone tissue was detectable for any of the scaffolds.

### 2.4 Histomorphology Evaluation after Implantation of 5 and 25 Weeks

Von Kossa/MacNeal's stain and Masson‐Goldner stain were employed to examine the detailed histological information surrounding and within the scaffolds. Histological staining confirmed the absence of mineralized bone in the front region of the Mg scaffold in week 5, although some collagen tissue was visible (Figure 4A1), and cartilage tissue was detected in the side region (Figure S9A, Supporting Information). No bone formation was observed inside the Mg scaffold in week 5, but collagen and osteoid tissue were present. In week 25, a compact mineralized bone layer, along with cartilage and osteoid, was found in the front region of the Mg scaffold, while mineralized bone penetrated into the scaffold and interconnected in the side, inside, and marrow regions.

In week 5, the front region of the Zn scaffold was covered by porous, mineralized bone, and a greater amount of collagen tissue was accumulating in this region (Figure 4A2). A small quantity of mineralized bone also formed in the marrow region, and collagen tissue appeared in the side region (Figure S9B, Supporting Information). Both staining methods confirmed that osteoid tissue with a spot of mineralized bone had already filled the interspaces within the Zn scaffold in week 5. By week 25, compact, mineralized bone layers covered the front and side regions of the Zn scaffolds. No mineralized bone was detected in the marrow region. Notably, the inside region of Zn scaffold in week 25 showed the similar outcome to week 5, as osteoid tissue filled the interior of the Zn scaffolds, which was further confirmed by EDS mapping that showed the osteoid tissue contained a significant amount of carbon, as well as calcium and phosphorus (Figure S10, Supporting Information). This was further confirmed by EDS mapping, which showed the osteoid tissue contained significant amounts of carbon, along with calcium and phosphorus (Figure S10, Supporting Information). However, only a small portion of the osteoid tissue transformed into mineralized bone.

Similar to Zn scaffolds, a porous mineralized bone layer covered the front region of the Ti scaffold in week 5, with a small amount of mineralized bone located in the marrow region (Figure 4A3; Figure S8C, Supporting Information). The bone in the side region also exhibited a porous feature, which contributed to the lower BIV for the Ti scaffold compared to the Zn scaffold at this time point. A small amount of bone tissue had already formed inside the Ti scaffolds in the early stage. In the late stage (week 25), there was no continuous bone layer present in the front region, only small residual bone tissue, indicating the occurrence of bone resorption. Additionally, a significant portion of cartilage tissue occupied the front region, and the non‐union tissue primarily consisted of fibrocartilage.^[^ ^48^ ^]^ Meanwhile, bone tissue only partially integrated with the Ti scaffolds in the side region, with the spaces between the scaffold and bone tissue filled with cytoplasm (muscle tissue). Instead of mineralized bone, only collagen tissue was observed inside the Ti scaffolds in week 25, indicating that degeneration of bone tissue also occurred in this region.

For the sham control group, bone regeneration in the defect region resulted in a two‐layer structure. Both outer and inner layers exhibited a porous structure in week 5, with the inner layer having a higher bone mass while the outer layer was attached to collagen tissue. By week 25, the outer layer transformed into a compact and thick bone layer, while the inner layer showed limited bone mass and thickness (Figure 4A4).

Osteoclast activity was visualized using tartrate‐resistant acid phosphatase (TRAP) staining (Figure 4B,C; Figure S11, Supporting Information). In week 5, TRAP‐positive osteoclasts were observed only in the side region of Mg and Ti scaffolds. In contrast, numerous TRAP‐positive osteoclasts were distributed in the front and side regions of the Zn scaffold, indicating high osteoclast activity. Strong TRAP staining was found in close proximity to the Zn scaffold, illustrating the aggregation of osteoclasts on its surface. By week 25, weak TRAP expression was observed in the front region of Mg scaffold and side and inside regions of Ti scaffolds. TRAP expression remained notably high surrounding Zn scaffolds, except in the inside region. For the sham control group, TRAP expression was consistently low in the outer layer at both time points, while the inner layer displayed multiple TRAP stains in week 5 and then became negative in week 25.

### 2.5 Analysis of Markers Expression in Different Regions

**Figure 5:**
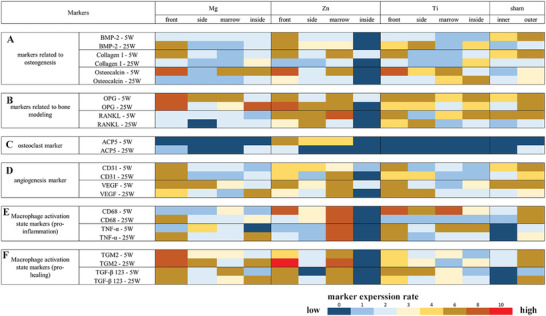
Rating mapping of markers expression. Rating mapping of marker expression related to A) osteogenesis, B) bone remodeling, C) osteoclasts, D) angiogenesis, E) M1, and F) M2 macrophages in Mg scaffold, Zn scaffold, Ti scaffold, and sham control groups. For Mg, Zn, and Ti scaffold groups, images for rating are taken in four regions: front, side, marrow, and inside regions. For the sham control group, two regions are assessed: inner bone and outer bone regions. Quantification of marker expression in each region is derived from IHC images (n = 5).

A semi‐quantitative analysis of immunohistochemistry (IHC) images was utilized to evaluate the expression of markers associated with various aspects of bone regeneration (**Figure** **5**). Tissue sections were obtained from polymethyl methacrylate (PMMA)‐embedded samples without decalcification, and as a result, markers could not differentiate within the mineralized bone. This study concentrated on the expression of markers in the peri‐implant region. Representative IHC images for each individual marker are depicted in Figures S12–S23 (Supporting Information), providing a visual representation of marker expression patterns.

Bone Morphogenetic Protein‐2 (BMP‐2), collagen I, and osteocalcin were analyzed as key osteogenesis markers. BMP‐2, secreted by osteoblasts and osteocytes into the bone matrix, plays a crucial role in inducing the differentiation of mesenchymal stem cells (MSCs) into osteoblasts.^[^ ^49^ ^]^ In the early stages (week 5), BMP‐2 expression displayed a uniform distribution across all regions of the Mg scaffold, indicating a well‐coordinated osteogenesis process around and within the Mg scaffold. In contrast, the Zn scaffold exhibited notably high BMP‐2 expression in the front region, while it remained undetectable inside the scaffold, highlighting potential differences in the spatial progression of bone formation. The Ti scaffolds revealed higher BMP‐2 expression in the front and side regions compared to other regions. By week 25, several interesting patterns emerged. The Mg scaffold showed a significant increase in BMP‐2 expression in the front region, suggesting robust bone formation over time. The Zn scaffold maintained high levels of BMP‐2 expression in the front, side, and marrow regions, indicating sustained osteogenic activity. For the Ti scaffold, BMP‐2 expression intensified in the front, side, and inside regions. These insights reveal a dynamic shift in osteogenic activity as the bone regeneration process progressed.

Collagen I, primarily produced by osteoblasts, constitutes 90% of the total collagen in bone tissue and plays a vital role in regulating the growth and osteogenic properties of osteoblasts through its distinct matrix composition.^[^ ^50^ ^]^ In week 5, all scaffolds exhibited strong collagen I expression in the front region, signifying the deposition of bone matrix in these areas. Notably, the marrow region of the Zn scaffold also demonstrated strong collagen I expression, indicating active bone matrix formation. By week 25, collagen I expression had decreased in the front region of all scaffolds. The formation of thick mineralized bone layers in the Mg and Zn scaffolds reduced the demand for bone matrix in their front regions. In the case of Ti scaffolds, bone tissue degeneration in the front region contributed to the decline in collagen I expression. Interestingly, the inside region of both Mg and Ti scaffolds displayed increased collagen I expression. This can be attributed to the ingrowth of bone tissue in Mg scaffolds and the development of cartilage tissue in Ti scaffolds. These findings highlight the dynamic changes in collagen I expression and its potential role in bone and cartilage tissue growth within various scaffold types.

Osteocalcin, a protein hormone secreted by osteoblasts, plays a crucial role in regulating bone matrix mineralization.^[^ ^51^ ^]^ In week 5, the front region of all scaffolds displayed strong osteocalcin expression, signifying high osteoblast activity and the robust initiation of hydroxyapatite crystal formation in this area. Concurrently, high osteocalcin expression was also observed in the marrow and inside regions of Mg scaffolds, the marrow region of Zn scaffolds, and the side and marrow regions of Ti scaffolds. By week 25, osteocalcin expression had decreased in most regions of all scaffolds, with the exception of the inside region of Ti scaffolds. As the formation of mineralized bone surrounding the scaffolds had already been established, osteocalcin levels were maintained at a lower level to ensure proper bone turnover.

The Receptor Activator of Nuclear Factor Kappa‐B Ligand/Receptor Activator of Nuclear Factor Kappa‐B (RANKL/RANK) signaling pathway regulates osteoclast formation, while Osteoprotegerin (OPG) protects bone from excessive resorption by binding to RANKL and preventing it from binding to RANK. Consequently, the RANKL/OPG ratio is a critical determinant of bone mass and skeletal integrity.^[^ ^52^, ^53^ ^]^ In weeks 5 and 25, OPG expression was stronger than RANKL in all regions of Mg scaffolds, suggesting that bone remodeling favored bone formation, particularly in the front and inside regions at week 25. For Zn scaffolds, RANKL expression was high in week 5, but higher OPG expression was observed in the front and marrow regions in week 25. In the case of Ti scaffolds, RANKL and OPG displayed similar levels in week 5, followed by stronger RANKL expression in the front and side regions in week 25. This finding aligns with the bone resorption observed in these regions of Ti scaffolds during the late stage. Additionally, a higher expression of OPG was noted in the marrow and inside regions of Ti scaffolds in week 25. Tartrate‐resistant acid phosphatase activity type 5 (ACP5 or TRAP) is highly expressed in osteoclasts and promotes the dephosphorylation of bone matrix phosphoproteins.^[^ ^54^ ^]^ ACP5 expression in tissue sections was generally weak in this study. Notable expression was observed surrounding Zn scaffolds in week 5, with only the front region of Zn scaffold and front/inside regions of Mg scaffold displaying low expression in week 25. No ACP5 expression was observed for Ti scaffolds.

Cluster of Differentiation 31 (CD31) and Vascular Endothelial Growth Factor (VEGF) are both well‐established markers of angiogenesis. CD31 is highly expressed on the surface of endothelial cells, and VEGF is a crucial growth factor and signaling molecule involved in angiogenesis.^[^ ^55^, ^56^ ^]^ As expected, the front region of all scaffolds in weeks 5 and 25 exhibited high expression of both angiogenesis markers, given that the periosteum of bone contains numerous blood vessels and bone regeneration in the defect region necessitates enhanced angiogenesis. In particular, the inside region of the Mg scaffold displayed strong VEGF expression in week 25, indicating significant angiogenesis penetration into the Mg scaffold in the late stage. Meanwhile, Zn scaffolds exhibited high expression of both markers in the marrow region, suggesting that Zn scaffolds can also stimulate angiogenesis at both ends. In week 25, the expression of both markers increased in the side region of Ti scaffolds, possibly due to the gap between Ti scaffolds and bone tissue being filled with muscle tissue containing blood vessels.

Cluster of Differentiation 68 (CD68) and Transglutaminase 2 (TGM2) are surface markers for pro‐inflammatory (M1) and pro‐healing (M2) macrophages, respectively.^[^ ^57^, ^58^, ^59^ ^]^ Tumor Necrosis Factor‐alpha (TNF‐*α*) and Transforming Growth Factor‐beta 1,2,3 (TGF‐*β*1,2,3) are secreted markers for pro‐inflammatory (M1) and pro‐healing (M2) macrophages, respectively,^[^ ^58^, ^60^, ^61^ ^]^ In week 5, the expression of pro‐inflammatory markers was weak for Mg scaffolds, but high expression occurred in the front and inside regions in week 25. Concurrently, pro‐healing markers displayed strong expression for Mg scaffolds in both week 5 and 25, with the front region consistently exhibiting high expression and the inside region showing higher levels compared to the marrow region in week 25. In week 5, Zn scaffolds elicited high CD68 expression in the front and marrow regions, maintaining high expression in the marrow region but decreasing in the front region by week 25. TNF‐*α* expression was predominantly high in the marrow region of Zn scaffolds in both weeks 5 and 25. The expression of pro‐healing markers remained consistent in the front and marrow regions of Zn scaffolds throughout the early and late stages. In week 5, pro‐inflammatory markers were generally high for Ti scaffolds, but only TNF‐*α* maintained high expression in the front region by week 25. Pro‐healing markers showed high expression in the front and side regions in week 5 but decreased in week 25. Notably, TGM2 expression was high in the inside region of the Ti scaffold.

Interestingly, the inside region of the Zn scaffold displayed distinct results. Only limited expression of Collagen I, OPG, CD31, and VEGF were detected in week 5, while other markers were negative in week 5, and no markers were expressed in week 25. In the sham control group, the outer layer exhibited higher osteogenesis markers compared to the inner layer, and a generally higher OPG‐to‐RANKL expression ratio in the outer layer. ACP5 was detected only in the outer layer in week 25, and angiogenesis markers showed greater expression in the outer layer. Additionally, both pro‐inflammatory and pro‐healing marker expressions were weaker in the inner layer compared to the outer layer.

### 2.6 Biomechanical Analysis

**Figure 6:**
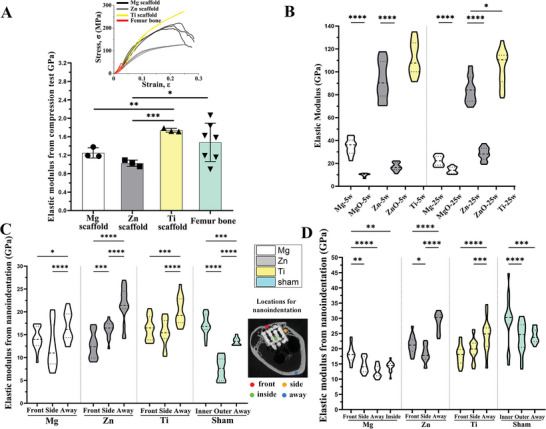
Mechanical analysis. A) Young's modulus of Mg, Zn, and Ti scaffolds and femur bone derived from the compression test. The inset figure shows the stress‐strain curves. B) Young's modulus of metals (Mg, Zn, and Ti) and oxides (MgO and ZnO) derived from the nanoindentation test on scaffolds after implantation of 5 and 25 weeks. Young's modulus derived from the nanoindentation test on bone tissue in different regions of the scaffold (front, side, inside, and away) and two bone layers of the sham control group (outer and inner) after implantation of 5 weeks C) and 25 weeks D). The inset figure indicates locations assessed with nanoindentation. Data are presented as means ± SEM. *P < 0.05, **P < 0.01, ***P < 0.001, ****P < 0.0001 (one‐way ANOVA).

The compression test was used to assess the bulk elastic modulus of scaffolds (**Figure** **6**A), while the nanoindentation test was employed to analyze the indentation modulus of metals and metal oxides at weeks 5 and 25 (Figure 6B). Mg and Zn scaffolds displayed comparable elastic moduli, with Zn scaffolds having a marginally higher value. Ti scaffolds, as anticipated, exhibited the highest modulus among all scaffolds. In the nanoindentation test, Mg metal displayed a significantly lower modulus compared to Zn metal, while Ti metal had the highest value. The low bulk modulus of Zn scaffolds did not correspond with the high indentation modulus of Zn metal, which was primarily attributed to the thin strut thickness and high cavity density within the strut (Figures S1 and S2, Supporting Information). Upon comparing metals and their corresponding metal oxides, it was evident that both Mg and Zn oxides displayed lower moduli than the metals themselves. An intriguing finding was the increase in the moduli of oxides by week 25, suggesting that the incorporation of Ca and P elements during the implantation period contributed to an enhancement in the mechanical strength of the degradation products.

The nanoindentation test was employed to examine the mechanical properties of bone tissue in various regions of the scaffolds. The “away bone” refers to the region in the same transverse plane, but opposite to the defect location. In week 5, the front and side bones surrounding Mg scaffolds displayed a lower modulus compared to the away bone. However, by week 25, the modulus of the front bone significantly increased, surpassing that of other regions. Notably, the inside bone of Mg scaffolds exhibited a modulus similar to the side and away bones, suggesting that it bore considerable mechanical loading. For Zn scaffolds, the away bone displayed the highest modulus at both week 5 and week 25. Interestingly, the modulus of the front bone was lower than that of the side bone in week 5, but this trend reversed by week 25. In the case of Ti scaffolds, the away bone demonstrated a higher modulus than the front and side bones in both week 5 and 25. In the sham groups, the inner bone layer had a higher modulus compared to the outer bone layer and away bone at weeks 5 and 25. The modulus of the outer bone layer increased significantly in week 25, reaching a level similar to the away bone. The hardness of the bone, as derived from the nanoindentation test, displayed a trend similar to that of the indentation modulus (Figure S24, Supporting Information).

### 2.7 Finite Element Analysis (FEA) to Demonstrate the Stress Distribution

**Figure 7:**
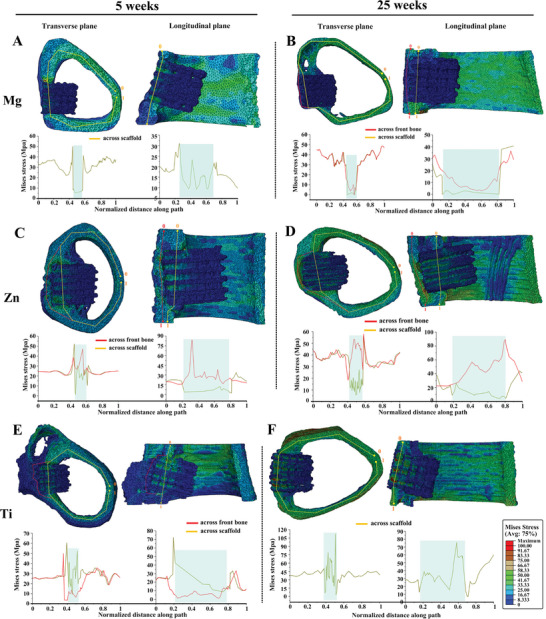
The von Mises stress distribution from FEA. The von Mises stress distribution of A) Mg‐5w, B) Mg‐25w, C) Zn‐5w, D) Zn‐25w, E) Ti‐5w, F) Ti‐25w models. Cross‐section views of each scaffold are displayed in transverse and longitudinal planes. Stress distribution is quantified along the yellow line across the scaffold and the red line across the front bone, with quantification results shown in the chart below. The blue area in the chart highlights the location of the scaffold. The deformation scale factor for all models is 1.

FEA was employed to analyze the distribution of the von Mises stress surround scaffolds (**Figure** **7**). Stress in the cross‐section of transverse plane of Mg‐5w model demonstrated a relatively uniform pattern in bone tissue, with significantly lower levels observed throughout the scaffold. The junction area between the bone tissue and the scaffold displayed peaks of stress distribution. A similar trend was seen in the longitudinal plane, although with a smaller gap between the bone tissue and the scaffold, and a few spots on the scaffold exhibited high peaks. Stress distribution around the Mg scaffold was not isotropic, with regions covered by bone tissue experiencing distinctly higher stress loading compared to regions exposed to the marrow cavity (Figure S25A, Supporting Information). For the Mg‐25w model, a significant portion of the scaffold exposed to the marrow cavity consisted of Mg oxides. Stress distribution across the scaffold was even lower than in the surrounding bone tissue in both transverse and longitudinal planes (Figure 7B). Notably, the stress distributed across the front bone was higher than that across the scaffold, indicating that the stress‐shielding effect on the front bone was limited. In comparison to week 5, stress loading on the Mg scaffold was significantly lower in week 25, and the distribution of stress was mainly observed in the region covered by bone tissue, without reaching the oxide region when the maximum limit was lowered (Figure S25B, Supporting Information). From the front view, looking toward the axial direction of the scaffold, stress distribution appeared to span across the entire Mg scaffold in week 5, and the stress on the newly formed bone in the front region was notably lower than that of the surrounding native bone tissue (Figure S26, Supporting Information).

The Zn‐5w model exhibited peaks in the junction area between the bone tissue and the Zn scaffold. However, the stress across the scaffold was only slightly lower than the surrounding bone tissue, with a gap much smaller than that observed in the Mg‐5w model. The stress distributed across the porous front bone layer was higher than the scaffold. A closer examination of the distribution on the scaffold revealed that the majority of stress was loaded on the region covered by bone tissue, with some stress also observed on the remaining parts of the scaffold (Figure S25C, Supporting Information). The stress distribution on the front bone in the Zn‐5w model was scattered, likely due to the porous structure of the newly formed bone at this early stage (Figure 7C; Figure S25C, Supporting Information). In the Zn‐25w model, the gap between the stress distribution across the scaffold and surrounding bone tissue widened compared to week 5. The stress across the front bone was higher than the scaffold and, notably, also higher than the surrounding bone tissue. The stress distribution on the front bone was uniform and at a high level (Figure S26D, Supporting Information), while the stress on the scaffold displayed a broader distribution compared to week 5 (Figure S25D, Supporting Information).

In the case of the Ti‐5w model, the stress distribution was more evenly spread across the scaffold compared to the adjacent bone tissue and the porous front bone layer (Figure 7E). The stress distribution on the Ti scaffold was broader than that of the other scaffolds in the early stage, and the front view showed that less stress was distributed on the front bone compared to the adjacent regions (Figures S24E and S25E, Supporting Information). For the Ti‐25w model, where no front bone was covering the scaffold, a high stress distribution on the scaffold was observed, with a broad distribution pattern similar to that of both week 5 and 25. The front view clearly showed a high stress distribution on the scaffold in week 25 (Figures S24F and S25F, Supporting Information).

## 3 Discussion

**Figure 8:**
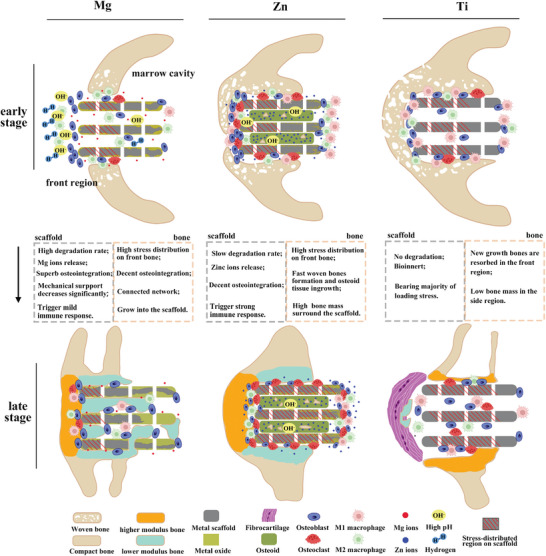
The schematic diagram of the interaction between scaffolds and bone regeneration. The schematic diagram illustrates the degradation trend of Mg and Zn scaffolds, along with bone regeneration strategies corresponding to different scaffolds in the early and late stages. Bone morphology and distribution are derived from Micro‐CT, SEM, and histochemical staining images, while cell distribution is based on TRAP staining and IHC staining.

Mg‐ and Zn‐based scaffolds exhibit unique and dynamic interactions with the surrounding bone tissue, which are characterized by differences in degradation behavior, biomechanical properties, and biological responses. These differences give rise to distinct regeneration strategies in the surrounding bone tissue. Understanding these interactions is crucial in the development of effective bone regeneration therapies (**Figure** **8**).

Degradation behavior and bone regeneration: The degradation behavior of scaffolds is a crucial factor that affects bone regeneration, while the growth of bone tissue can also influence the local physiological environment and thereby affect the degradation process of scaffolds. Among the three types of scaffolds investigated in this study, Mg scaffolds exhibited the highest degradation activity due to the high corrosion potential of Mg‐based alloys in the presence of chloride ions. While Mg ions are beneficial for osteogenesis, the intense degradation of Mg metal can cause a significant elevation in pH value and hydrogen evolution in the peri‐implant area, which may hinder bone regeneration in the early stage.^[^ ^62^ ^]^ The absence of bone formation in the front region of Mg scaffolds during the early stage can be attributed to rapid degradation. However, a high activity of osteoblasts was observed in the front region of Mg scaffolds in week 5, signifying osteogenesis progression. To mitigate the influence of Mg degradation in the early stage for a faster healing process in the future, an option is to employ coating techniques to reduce the initial degradation rate. Various types of coatings on Mg‐based materials have demonstrated promising anticorrosion properties while also enhancing biocompatibility.^[^ ^63^, ^64^ ^]^ In contrast, Zn metals exhibited a slow and mild degradation process, causing less increase in pH value and no hydrogen evolution. Moreover, Zn ions have been reported to promote osteogenesis.^[^ ^35^, ^36^, ^65^ ^]^ In the early stage, newly formed bone tended to wrap around Zn scaffolds, resulting in the robust growth of porous bone layers (woven bone) in front and side regions of Zn scaffolds, as well as the osteoid inside the scaffolds. Ti scaffolds, as a bio‐inert metal, demonstrated physiological stability and osteoconductivity in week 5, with decent bone mass in front and side regions. However, the absence of osteoid inside Ti scaffolds suggested that the osteoconductivity in this region was inferior to that of Zn scaffolds.

The adverse effect of Mg degradation on bone regeneration was limited to the initial phase, and the growth of front and side bone was integrated closely with Mg scaffolds (Figure 2A). The bone region of Mg scaffold only showed a thin oxide layer due to the limited penetration of body fluid caused by the bone tissue on the surface of Mg scaffold, which blocked the mass transfer process of the degradation. In contrast, the marrow region of Mg scaffold had more areas exposed to the body fluid and more metals were converted to oxides. A similar trend was observed for Zn scaffolds in week 25, where more Zn metal was converted to Zn oxides in the marrow region than the bone region. Despite the small gaps in the implant‐bone interface and cavities within the Zn scaffold, the degradation progress was still slow in the bone region. This can be attributed to the compact and continuous bone layers surrounding the bone region of Zn scaffold and the osteoid inside Zn scaffolds. Unlike the degradation of Mg metals, the degradation process of Zn metal requires the involvement of Oxygen. Bone layers can block the oxygen diffusion to the bone region of Zn scaffolds and thus lower the degradation rate.

It is notable that the osteoid tissue penetrated the Zn scaffolds in the early stage but only a small portion can be converted into mineralized bone after 25 weeks of implantation. According to the IHC results (Figure 5; Figure S27, Supporting Information), the osteoid tissue inside Zn scaffolds showed low biological activity, and there was a clear boundary of this inactive tissue located at the end of the Zn scaffold exposed to the front bone tissue. The main reason for the failure of the osteoid tissue to mineralize is likely due to the local aggregation of Zn ions, which causes local toxicity and interferes with ossification.^[^ ^66^, ^67^, ^68^ ^]^ The compact structure and slow degradation rate of the Zn scaffold prevented sufficient mass exchange inside the scaffold and led to the aggregation of Zn ions, as observed in EDX mapping images of osteoid tissue located inside Zn scaffolds (Figure S10, Supporting Information). To achieve more bone formation inside the Zn scaffold, two promising approaches can be explored in future studies. One is the optimization of scaffold design; the degradation behavior of Zn scaffolds can be modulated with different structures.^[^ ^42^, ^46^ ^]^ and a higher porosity design could promote bone tissue ingrowth and mass/ion transfer.^[^ ^69^, ^70^, ^71^ ^]^ Another direction is to develop alloys with lower Zn content and better osteocompatibility; while Zn‐Mg alloys continue to draw attention, Zn‐Ca, Zn‐Mn, and Zn‐Mg‐Mn alloys have been developed.^[^ ^72^, ^73^, ^74^, ^75^ ^]^

The inhomogeneous degradation progress of Mg and Zn scaffolds highlights an important consideration for the design and clinical application of bioresorbable metal scaffolds. Depending on their location within the body, different regions of a scaffold may be exposed to varying local physiological environments, such as bone tissue‐bone marrow cavity for long bones, skin tissue‐cranial cavity for skull bones, and muscle‐bone tissue for facial skeleton. To provide sufficient mechanical or functional support, the region that undertakes the majority of mechanical loading or is responsible for function should have a relatively lower degradation rate compared to other regions. In this study, a faster degradation progress in the bone region of Mg and Zn scaffolds was desired, as this region exhibited the highest stress distribution in both week 5 and 25 (Figure S25, Supporting Information). Furthermore, if the bone region of the scaffold degrades before the marrow region, it could result in the displacement or dislocation of the scaffold from the implantation site. Consequently, a higher degradation preference for the marrow region of scaffolds is more favorable for maintaining the overall stability of the implant.

Biomechanical properties: The bulk elastic modulus of scaffolds is a critical factor in the success of implantation since it should closely match the elastic modulus of surrounding bone tissue. The porous structure of the scaffolds significantly reduced the bulk elastic modulus compared to that of their corresponding metals, approximating the modulus of rabbit femur bone (Figure 6A,B). However, bone resorption in the front region of the Ti scaffold revealed a stress shielding effect, as evidenced by the low elastic modulus of the residual front bone and high stress distribution on the Ti scaffold. This effect was also present in the side region of the Ti scaffold, demonstrated by a noticeable gap between the side bone and scaffolds.

In contrast, Mg and Zn scaffolds showed no signs of stress shielding. The bulk elastic modulus of these scaffolds was anticipated to be lower in the later stage of implantation due to the significantly reduced modulus of oxides compared to metals. Stress distribution favored the front bone over Mg and Zn scaffolds, resulting in a higher modulus for the front bone compared to the side bone. In the case of Mg scaffolds, the bone inside displayed an interconnected structure with a respectable elastic modulus. Alongside the front and side bones, this formed a bone network that shared the majority of stress loading, resulting in low stress distribution on Mg scaffolds in week 25 within a confined region. For Zn scaffolds, the stress distribution in week 25 was almost evenly spread across the entire scaffold and was expected to be transmitted to the tissue inside. The lack of calcification in the osteoid tissue within Zn scaffolds was unlikely due to low stress distribution. Conversely, the increased thickness of the front bone layer in front of Zn scaffolds may have resulted from compensating for the absence of internal bone.

Angiogenesis: Bone tissue growth and maintenance rely on adequate oxygen and nutrient supply, making angiogenesis essential for bone regeneration. The robust expression of CD31 and VEGF markers in the front region of Mg and Zn scaffolds at weeks 5 and 25 aligned with the strong growth of front bone (Figure 5D). Angiogenesis markers displayed higher expression in the marrow region of Mg scaffolds compared to the inside region at week 5, but this relationship reversed by week 25, suggesting the concurrent ingrowth of blood vessels and bone tissue. (Figure 5D). In contrast to Mg scaffolds, the inside region of Zn scaffolds exhibited no angiogenesis marker expression at week 25, while the marrow region showed strong expression despite the absence of bone formation in the later stage. Zn ions have been reported to promote angiogenesis, and the high degradation activity in the marrow region of Zn scaffolds led to a locally elevated concentration of Zn ions and intensified angiogenesis.^[^ ^76^, ^77^ ^]^ Like the front bone of Mg and Zn scaffolds, the outer bone layer of the sham control group exhibited higher angiogenesis activity than the inner bone layer, indicating that increased osteogenesis was accompanied by heightened angiogenesis. Although fibrocartilage tissue is avascular, the front region of Ti scaffolds at week 25 still displayed high osteogenesis, potentially due to the presence of residual small bone tissue.

Immune response/osteoclast activity: The immune response to a scaffold can vary significantly based on material properties and other factors, and plays a critical role in tissue regeneration outcomes. We first examined the systemic inflammatory levels by measuring C‐reactive protein (CRP) levels in the serum (Figure S28, Supporting Information). CRP, an acute‐phase plasma protein, is commonly used as a biomarker in toxicity studies.^[^ ^78^ ^]^ The results revealed that the Mg scaffold group exhibited the highest systemic toxicity at week 5, likely due to the intense degradation activity of Mg during the early stage. At this time point, the Ti group displayed the lowest CRP levels among all groups, as expected for a bio‐inert material. CRP levels across all groups decreased to similar levels by week 10, and no significant systemic toxicity was observed from week 15 onwards.

We assessed the local immune response near the scaffolds by analyzing macrophages and osteoclasts activity. Osteoclasts play a crucial role in osteogenesis and bone remodeling, and are known to derive from macrophages.^[^ ^79^ ^]^ Osteoclastogenesis occurs when M‐CSF and RANKL stimulate monocytes/macrophages, leading to their fusion and differentiation into osteoclasts.^[^ ^80^ ^]^

Although the degradation activity of Mg scaffolds was high in week 5, M1 macrophage expression remained low, while M2 macrophage expression was elevated (Figure 5E,F). This pattern indicates that the local immune response was conducive to defect healing, with M2 macrophages promoting tissue repair and regeneration. In week 25, both M1 and M2 macrophages exhibited strong expression in the front and inside regions of Mg scaffolds, which corresponded to the high osteogenesis activity observed in these areas. The elevated M1 macrophage activity could potentially be attributed to the ongoing degradation of Mg scaffolds, as these macrophages are associated with inflammatory responses to foreign materials and tissue damage. Interestingly, despite the distinct macrophage activity, RANKL expression surrounding Mg scaffolds remained weak throughout the study, both at week 5 and 25. This low RANKL expression coincided with weak osteoclast activity observed in the TRAP staining results (Figure 4B,C), suggesting that osteoclast formation and activity were not significantly influenced by the release of Mg ions or the scaffold degradation process.

Unlike the nutritional impact of Zn, which demonstrates a decrease in osteoclast resorption activities,^[^ ^36^ ^]^ the localized release of Zn ions from the scaffold actually enhances osteoclast activities. A notably high expression of both M1 and M2 macrophages was observed in the front region of the Zn scaffold in week 5, corresponding to early bone formation. Zn ions have long been recognized for their immunomodulatory properties and their close association with macrophage functions.^[^ ^81^, ^82^ ^]^ In week 25, M2 macrophages were predominantly expressed in the front region of the Zn scaffold, indicating that bone formation was the primary activity in this area, with a greater involvement of pro‐healing macrophages. However, in the marrow region, the high local concentration of Zn ions was speculated to be the main factor triggering the strong expression of both M1 and M2 macrophages. Notably, RANKL expression was high around Zn scaffolds, promoting the differentiation of neighboring macrophages into osteoclasts, and leading to the aggregation of osteoclasts in the front, side, and marrow regions of Zn scaffolds. These osteoclasts were also responsible for the small gaps observed between Zn scaffolds and front/side bone tissue (Figure 3E).

In contrast to Mg and Zn scaffolds, Ti scaffolds exhibited significantly lower macrophage marker expression, indicating that scaffolds composed of bio‐inert metal elicit a substantially weaker immune response from surrounding tissue. However, this reduced immune response does not always favor bone regeneration, emphasizing the need for a balanced immune response to achieve optimal healing outcomes.

## 4 Conclusion

After conducting in vivo comparative study of bioresorbable Mg and Zn scaffolds, as well as bio‐inert Ti scaffolds, we gained valuable insights into the interaction between bone tissue and these metal scaffolds during the healing process. Our investigation revealed that the degradation behavior of biodegradable metal scaffolds was influenced by the local physiological environment, with significantly higher degradation rate in regions exposed to the bone marrow cavity compared to those covered by bone tissue. Bone regeneration outcomes varied among scaffold types: Mg scaffolds led to delayed but complete defect healing with bone ingrowth; Zn scaffolds encouraged early healing and the high quality of newly‐formed bone, despite limited calcification of osteoid inside the scaffold; and Ti scaffolds presented bone resorption and inferior osseointegration. Real geometry FEA emphasized stress distribution predominantly on the newly formed bone for bioresorbable scaffolds, while Ti scaffolds exhibited a noticeable stress‐shielding effect. IHC staining revealed that scaffold types and regions could trigger distinct biological responses, including osteogenesis, bone modeling, angiogenesis, and macrophage activation. Our study provides a comprehensive understanding of the metal scaffold‐assisted bone healing process, and we found that the dynamic interaction between bone tissue and bioresorbable scaffolds (Mg and Zn) is favorable for the bone defect healing process, making them promising candidate for bone regeneration applications.

## 5 Experimental Section

### Study Design

Three additively manufactured metal scaffolds—pure Ti (grade 1), Mg alloy (Mg4Y3REZr), and Zn alloy (Zn1Mg)—in a rabbit critical femur bone defect model was investigated to examine their distinct interaction patterns with bone regeneration and their impact on defect healing. The scaffolds, fabricated using L‐PBF under the same blueprinting, were labeled as Ti, Mg, and Zn scaffolds for simplicity. While Ti scaffolds were bio‐inert implants, Mg and Zn scaffolds were biodegradable, exhibiting different degradation behaviors. A circular defect with a 4 mm diameter was drilled into the distal metaphysis of the rabbit femur, and one scaffold was inserted. Bone samples and scaffolds were collected at weeks 5 and 25. The biodegradation behavior of Mg and Zn scaffolds in different regions was characterized to analyze the influence of local physiological environments on degradation tendencies. Micro CT, SEM analysis, histological, and immunohistochemical images were utilized to evaluate bone regeneration surrounding the scaffolds. Compression tests determined the bulk elastic modulus of scaffolds pre‐implantation, while nanoindentation tested the modulus of metal and oxide post‐implantation. Nanoindentation was employed to assess the mechanical properties of bone in various regions, and used FEA techniques to analyze mechanical loading distribution on bones and scaffolds, visualizing the stress‐shielding effect. A total of 31 animals were involved, with 30 reaching the collection point and one excluded due to an accidental fracture. In the Mg and Zn scaffold groups, four animals were collected in week 5, and six in week 25. For the Ti scaffold group, three animals were collected in weeks 5 and 25, respectively. Last, for the sham control group, two animals were collected in weeks 5 and 25, respectively.

### Metal Scaffolds Fabrication

The three powder materials, fabricated using gas atomization and subsequently sieved, achieved a particle size distribution of 25–63 µm, ideal for L‐PBF. The clinically used Mg alloy WE43MEO (Mg4Y3REZr) and Zn alloy ZM1MEO (Zn1Mg) were processed on a laboratory‐scale L‐PBF machine utilizing optimized parameters to maximize relative density (Meotec GmbH, Aachen), as reported in prior studies.^[^ ^83^ ^]^ All scaffolds shared identical blueprinting specifications, including a 250 µm strut thickness and a 500 µm pore size. The scaffold's unit structure consisted of a body‐centered cubic unit cell, yielding eight struts at each node. The Ti powder (Ti grade 1) was processed on an industrial‐scale L‐PBF machine, featuring a 50 µm spot size, 160 W laser power, 500 mm s^−1^ scan speed, 30 µm layer thickness, and 40 µm hatching distance. The actual parameters of each material exhibited minor deviations from the intended design (Figure S1, Supporting Information). Following manufacturing, each scaffold underwent manual sandblasting at a maximum pressure of 3 bar, using ZrO particles. Residues from sandblasting were removed via an ultrasonic cleaning bath in ethanol. Figure 1C presents the design file and post‐processed implants, along with their respective dimensions. The vertical pores were fully open, while the designed horizontal pores were only partially open. A static compression test was conducted to assess the compression modulus of the scaffolds.

### Critical‐size Femur Defects Model in Rabbit

Thirty‐one female New Zealand White rabbits (mean ± SD weight, 3.0 ± 0.3 kg; age ≥ 12 weeks) were included in this study. Animal protocols were approved by the Stony Brook University Institutional Animal Care and Use Committee (IACUC). Rabbits were anesthetized using ketamine/xylazine through IV injection and maintained with 2% isoflurane‐oxygen inhalant throughout surgery. A physiological monitoring system tracked the animals' temperature, heart rate, and respiration until surgery completion. The surgical site's skin was sterilized, and a 2 cm incision was made longitudinally along the distal femur using a scalpel. The femur was separated from the attaching muscles with a retractor. A cylindrical hole (4 mm in diameter) was drilled in the distal metaphysis of the right femur on the lateral side. Initial bleeding was controlled with a hemostatic sponge. The scaffold was inserted into the cylindrical hole and pressed inward until it was level with the compact bone. Most bleeding ceased after scaffold insertion. The incision was closed using 4‐0 resorbable polydioxanone sutures in layers and left un‐casted. Rabbits were kept in a veterinary incubator until they fully regained consciousness. A transdermal fentanyl patch was applied to the rabbits' backs immediately post‐operation and for three more days thereafter. The rabbits' health was monitored daily for one week, and weight loss was assessed one week post‐operation. Rabbits undergoing the same operation without scaffold insertion were analyzed as sham controls.

### Follow‐Up Tissue Collection

Blood collection was performed every 5 weeks until week 25 post‐surgery via the ear vein. Serum was separated and collected from the blood samples to assess CRP) levels using CRP kits. Serum samples collected in week 25 were sent to a qualified agent for blood chemistry panel analysis. Rabbits were euthanized with an injection of sodium pentobarbital. The entire femur bone, including the inserted scaffold, was harvested in weeks 5 and 25 and fixed in 10% neutral buffered formalin at 4 °C for 48 h, followed by further fixation in 70% ethanol for two weeks. Organs such as the heart, kidney, liver, spleen, and lung were collected in week 25. Harvested organs were immediately sliced and fixed in 10% neutral buffered formalin at 4 °C for 24 h, then subjected to serial dehydration, paraffin embedding, and sectioning into 4 µm slices. Organ sections were stained with hematoxylin and eosin (H&E) to evaluate systemic toxicity.

### Micro‐CT

Micro‐CT analysis was conducted on samples immersed in 70% ethanol to visualize scaffold degradation and bone regeneration at weeks 5 and 25. The micro‐CT parameters, including 80 kV voltage and 125 µA current, were kept consistent for all samples. Metal‐based artifacts in micro‐CT images were particularly noticeable in the case of Zn scaffolds (Figure S7, Supporting Information). Consequently, the 3D reconstruction of Zn scaffolds and surrounding bone was derived not only from micro‐CT data but also incorporated optimization based on SEM and histological staining images.

### Histological Preparation and Evaluation

Following fixation, undecalcified femur samples were sectioned, retaining the distal metaphysis region with the scaffold. Samples underwent PMMA embedding. For histological staining, all samples were sectioned in the mid‐transverse plane of the bone to visualize scaffold regions adjacent to the bone defect and exposed to bone marrow. The 4‐µm sections obtained using a microtome were stained with Von Kossa/MacNeal's and Masson‐Goldner techniques to distinguish tissue types and showcase tissue morphologies. Osteoclast quantity and activity were visualized through TRAP staining. IHC was employed to analyze protein expression surrounding the scaffolds. The 4‐µm sections were deplastified in xylene and underwent serial rehydration, endogenous peroxidase suppression, and enzymatic antigen retrieval. Chromogenic IHC staining of prepared section samples was performed by optimizing protocols from DAB‐staining kits. Several groups of markers targeted specific cellular activities, including osteogenesis (osteocalcin/collagen I/BMP‐2), bone modeling (OPG/RANKL), osteoclast activity (ACP5), angiogenesis (CD31/VEGF‐A), pro‐inflammatory macrophage (CD68/TNF‐*α*), and pro‐healing macrophage (TGM2/TGF‐*β*123). The marker‐positive area ratio in the peri‐implant region within 200–300 µm was quantified using ImageJ Fiji.^[^ ^84^ ^]^ Marker expression in different regions of scaffolds was semi‐quantified and rated based on the marker‐positive area ratio and expression intensity, with details provided in Tables S2 and S3 (Supporting Information).^[^ ^85^ ^]^

### Scanning Electron Microscopy

PMMA‐embedded samples were ground to expose the mid‐sagittal plane of the scaffold and subsequently polished to a 1 µm finish for microscopy. The samples were then gold sputtered to enhance conductivity. Prepared samples were examined using SEM to reveal detailed degradation behavior on scaffolds and bone‐implant interfaces. EDS and elemental mapping were utilized to visualize metal oxides and determine their composition.

### Mechanical Properties of Bone Tissues Surrounding Scaffolds

Nanoindentation was employed to determine the Young's modulus of bone in different regions and that of scaffold materials, including metals and oxides. PMMA‐embedded samples were ground to expose the mid‐sagittal plane of the scaffold. The resulting embedded beams were further ground to create a horizontal testing surface and polished to a 1 µm finish. Nanoindentation testing was conducted using a TI 950 Triboindenter, employing a diamond Berkovich indenter tip and a 3×2 indentation array with an interval spacing of 1.5 µm. The mechanical behavior of the femur bone within the defect region was investigated under static axial compression using a tabletop test system (MTS 858 Mini Bionix II). Following fixation, bone samples collected from the Mg and Zn groups at 25 weeks were sawed, and the defect region was preserved. The resulting sections were ground to achieve two flat surfaces perpendicular to the bone's axis. Static compression was applied at a rate of 1 N s^−1^. The left femur without a bone defect was also tested with a static compression test to obtain the standard modulus value of femur bone in this study.

### Finite Elements Analysis

FE models for all scaffold groups were established to analyze the stress distribution of mechanical loading on the scaffold and surrounding bones. FE models were derived from micro‐CT results, including the scaffold and surrounding bones. The model for the Mg scaffold at week 25 also incorporated degradation products (mainly Mg oxides) on the scaffold. Models of Zn and Ti scaffolds were optimized based on SEM and histological images to counteract metal‐induced artifacts in micro‐CT results. FEA was performed using Abaqus (v2016, Dassault Systemes, RI, USA), with a loading regimen of 1000 N applied to the top surface and the bottom fixed. Material properties used in FE analysis were derived from compression tests as follows: Bone, Young's modulus: 1486 MPa, Poisson's ratio: 0.3; Pure Ti (scaffold), Young's modulus: 1741 MPa, Poisson's ratio: 0.3; Zn metal, Young's modulus: 1026 MPa, Poisson's ratio: 0.25; Mg metal, Young's modulus: 1250 MPa, Poisson's ratio: 0.3. Specifically, considering the indentation modulus of Mg oxides at week 25 was 63% of Mg metal, the Young's modulus of Mg oxides was set to 789 MPa in FEA, with a Poisson's ratio of 0.18.

### Statistical Analysis

Statistical analysis was performed using SPSS version 26.0 (IBM Corp., USA) and GraphPad Prism version 9.0 (GraphPad Software, USA). Data were presented as mean ± standard deviation. One‐way ANOVA followed by Tukey's post hoc test was employed to compare multiple groups. Two‐way ANOVA followed by Bonferroni's post hoc test was utilized to analyze the interaction between different factors. A p‐value <0.05 was considered statistically significant. A semi‐quantification scoring system was used for the IHC analysis. The femur bone tissue samples with Mg and Zn scaffolds used in the compression test had only two duplicate specimens; therefore, only the stress–strain curves were listed in the supplemental material for reference.

## Conflict of Interest

The authors declare no conflict of interest.

## Author Contributions

J.Z. wrote the animal ethics approval and manuscript, prepared images, and performed various tasks including animal care, anesthesia, surgeries, tissue collection, electro spectroscopy, and histological analysis, results interpretation, radiographic analysis and interpretation, biomechanical analysis and interpretation, and FEA analysis. E.G. conducted animal care, anesthesia, surgeries, biomechanical analysis, and FEA analysis. Y.S. contributed to animal care, anesthesia, surgeries, and radiographic analysis. J.Z. contributed to histological analysis, biomechanical analysis, and FEA analysis. N.K. contributed to scaffold and surface design and revised preclinical and in vitro results. F.B. contributed to the design and fabrication of prototypes. A.K. supervised prototype manufacturing and revised the manuscript. Y.Q. provided guidance and participated in animal care, anesthesia, and surgeries. D.Z. designed the study, supervised its performance and evaluation, and wrote and revised the manuscript, as well as securing external funding.

## Supporting information

Supporting Information

## Data Availability

The data that support the findings of this study are available in the supplementary material of this article.

## References

[1] C. G. Finkemeier , JBJS 2002, 84, 454. 10.2106/00004623-200203000-00020 11886919

[2] R. Capanna , D. A. Campanacci , N. Belot , G. Beltrami , M. Manfrini , M. Innocenti , M. Ceruso , Orthop. Clin. North Am. 2007, 38, 51. 17145294 10.1016/j.ocl.2006.10.008

[3] L. Zhang , G. Yang , B. N. Johnson , X. Jia , Acta Biomater. 2019, 84, 16. 30481607 10.1016/j.actbio.2018.11.039

[4] C. Wang , W. Huang , Y. Zhou , L. He , Z. He , Z. Chen , X. He , S. Tian , J. Liao , B. Lu , Y. Wei , M. Wang , Bioact. Mater. 2020, 5, 82. 31956737 10.1016/j.bioactmat.2020.01.004 PMC6962643

[5] M. A. A. Ansari , A. A. Golebiowska , M. Dash , P. Kumar , P. K. Jain , S. P. Nukavarapu , S. Ramakrishna , H. S. Nanda , Biomater. Sci. 2022, 10, 2789. 35510605 10.1039/d2bm00035k

[6] F. S. L. Bobbert , K. Lietaert , A. A. Eftekhari , B. Pouran , S. M. Ahmadi , H. Weinans , A. A. Zadpoor , Acta Biomater. 2017, 53, 572. 28213101 10.1016/j.actbio.2017.02.024

[7] R. Wauthle , S. M. Ahmadi , S. Amin Yavari , M. Mulier , A. A. Zadpoor , H. Weinans , J. Van Humbeeck , J.‐P. Kruth , J. Schrooten , Mater. Sci. Eng. C. Mater. Biol. Appl. 2015, 54, 94. 26046272 10.1016/j.msec.2015.05.001

[8] C. Yan , L. Hao , A. Hussein , P. Young , D. Raymont , Mater. Des. 2014, 55, 533.

[9] R. Wauthle , J. Van Der Stok , S. Amin Yavari , J. Van Humbeeck , J. P. Kruth , A. A. Zadpoor , H. Weinans , M. Mulier , J. Schrooten , Acta Biomater. 2015, 14, 217. 25500631 10.1016/j.actbio.2014.12.003

[10] S. Limmahakhun , A. Oloyede , K. Sitthiseripratip , Y. Xiao , C. Yan , Mater. Des. 2017, 114, 633.

[11] Y. Qin , P. Wen , H. Guo , D. Xia , Y. Zheng , L. Jauer , R. Poprawe , M. Voshage , J. H. Schleifenbaum , Acta Biomater. 2019, 98, 3. 31029830 10.1016/j.actbio.2019.04.046

[12] X. Pei , L. Wu , C. Zhou , H. Fan , M. Gou , Z. Li , B. Zhang , H. Lei , H. Sun , J. Liang , Q. Jiang , Y. Fan , X. Zhang , Biofabrication 2021, 13, 015008. 10.1088/1758-5090/abdb89 35034011

[13] H.‐K. Lim , M. Ryu , S.‐H. Woo , I.‐S. Song , Y.‐J. Choi , U.‐L. Lee , Materials 2021, 14, 3892. 34300810 10.3390/ma14143892 PMC8303426

[14] Y. Gu , Y. Sun , S. Shujaat , A. Braem , C. Politis , R. Jacobs , J.Orthop. Surg. Res. 2022, 17, 68. 35109907 10.1186/s13018-022-02960-6 PMC8812248

[15] S. Cleemput , S. E. F. Huys , R. Cleymaet , W. Cools , M. Y. Mommaerts , Biomater. Res. 2021, 25, 18. 34112248 10.1186/s40824-021-00216-8 PMC8191027

[16] S. Barui , S. Chatterjee , S. Mandal , A. Kumar , B. Basu , Mater. Sci. Eng., C 2017, 70, 812. 10.1016/j.msec.2016.09.040 27770959

[17] K. S. Hamid , S. G. Parekh , S. B. Adams , Foot & Ankle Int. 2016, 37, 433. 10.1177/1071100715620895 26764314

[18] H. R. Cho , T. S. Roh , K. W. Shim , Y. O. Kim , D. H. Lew , I. S. Yun , Arch. Craniofac. Surg. 2015, 16, 11. 28913212 10.7181/acfs.2015.16.1.11 PMC5556788

[19] E.‐K. Park , J.‐Y. Lim , I.‐S. Yun , J.‐S. Kim , S.‐H. Woo , D.‐S. Kim , K.‐W. Shim , J. Craniofac. Surg. 2016, 27, 943. 27192643 10.1097/SCS.0000000000002656

[20] L. Zhang , G. Yang , B. N. Johnson , X. Jia , Acta Biomater. 2019, 84, 16. 30481607 10.1016/j.actbio.2018.11.039

[21] C. Schlundt , H. Schell , S. B. Goodman , G. Vunjak‐Novakovic , G. N. Duda , K. Schmidt‐Bleek , J. Exp. Orthop. 2015, 2, 1. 26914869 10.1186/s40634-014-0017-6 PMC4545842

[22] S. Egawa , S. Miura , H. Yokoyama , T. Endo , K. Tamura , Dev. Growth Differ. 2014, 56, 410. 24860986 10.1111/dgd.12136

[23] J. D. Boerckel , Y. M. Kolambkar , H. Y. Stevens , A. S. P. Lin , K. M. Dupont , R. E. Guldberg , J. Orthop. Res. 2012, 30, 1067. 22170172 10.1002/jor.22042 PMC3307871

[24] F. Wang , X. Cai , Y. Shen , L. Meng , Bioact. Mater. 2023, 23, 16. 36406245 10.1016/j.bioactmat.2022.10.029 PMC9650009

[25] C. Murphy , F. O'Brien , D. Little , A. Schindeler , Eur. Cell Mater. 2013, 26, 120. 24052425 10.22203/ecm.v026a09

[26] C. Li , C. Guo , V. Fitzpatrick , A. Ibrahim , M. J. Zwierstra , P. Hanna , A. Lechtig , A. Nazarian , S. J. Lin , D. L. Kaplan , Nat. Rev. Mater. 2020, 5, 61.

[27] G. Chandra , A. Pandey , Biocybern. Biomed. Eng. 2020, 40, 596.

[28] H. Li , Y. Zheng , L. Qin , Prog. Nat. Sci.: Mater. Int. 2014, 24, 414.

[29] M. Heiden , E. Walker , L. Stanciu , J. Biotechnol. Biomater. 2015, 5, 1.

[30] Y. Lai , Y. Li , H. Cao , J. Long , X. Wang , L. Li , C. Li , Q. Jia , B. Teng , T. Tang , J. Peng , D. Eglin , M. Alini , D. W. Grijpma , G. Richards , L. Qin , Biomaterials 2019, 197, 207. 30660996 10.1016/j.biomaterials.2019.01.013

[31] J. Long , W. Zhang , Y. Chen , B. Teng , B. Liu , H. Li , Z. Yao , D. Wang , L. Li , X.‐F. Yu , L. Qin , Y. Lai , Biomaterials 2021, 275, 120950. 34119886 10.1016/j.biomaterials.2021.120950

[32] S. Bose , G. Fielding , S. Tarafder , A. Bandyopadhyay , Trends Biotechnol. 2013, 31, 594. 24012308 10.1016/j.tibtech.2013.06.005 PMC3825404

[33] T. Qi , J. Weng , F. Yu , W. Zhang , G. Li , H. Qin , Z. Tan , H. Zeng , Biol. Trace Elem. Res. 2021, 199, 559. 32449009 10.1007/s12011-020-02183-y

[34] X. Chen , B. Tan , S. Wang , R. Tang , Z. Bao , G. Chen , S. Chen , W. Tang , Z. Wang , C. Long , W. W. Lu , D. Yang , L. Bian , S. Peng , Biomaterials 2021, 274, 120895. 34020269 10.1016/j.biomaterials.2021.120895

[35] M. Yamaguchi , M. N Weitzmann , Mol. Cell. Biochem. 2011, 355, 179. 21533765 10.1007/s11010-011-0852-z

[36] K. B. Hadley , S. M. Newman , J. R. Hunt , J. Nutr. Biochem. 2010, 21, 297. 19369052 10.1016/j.jnutbio.2009.01.002

[37] L. Jauer , W. Meiners , B. Jülich , M. Voshage , Eur. Cells Mater. 2015, 30, 1.

[38] Y. Li , J. Zhou , P. Pavanram , M. A. Leeflang , L. I. Fockaert , B. Pouran , N. Tümer , K. U. Schröder , J. M. C. Mol , H. Weinans , H. Jahr , A. A. Zadpoor , Acta Biomater. 2018, 67, 378. 29242158 10.1016/j.actbio.2017.12.008

[39] C. Liu , M. Zhang , C. Chen , Mater. Sci. Eng., A 2017, 703, 359.

[40] X. Yue , J. Shang , M. Zhang , B. Hur , X. Ma , Mater. Sci. Eng., A 2022, 859, 144167.

[41] N. Sezer , Z. Evis , M. Koç , J. Magnesium Alloys 2021, 9, 392.

[42] Y. Qin , P. Wen , M. Voshage , Y. Chen , P. G. Schückler , L. Jauer , D. Xia , H. Guo , Y. Zheng , J. H. Schleifenbaum , Mater. Des. 2019, 181, 107937.

[43] P. Wen , Y. Qin , Y. Chen , M. Voshage , L. Jauer , R. Poprawe , J. H. Schleifenbaum , J. Mater. Sci. Technol. 2019, 35, 368.

[44] Y. Yang , F. Yuan , C. Gao , P. Feng , L. Xue , S. He , C. Shuai , J. Mech. Behav. Biomed. Mater. 2018, 82, 51. 29567530 10.1016/j.jmbbm.2018.03.018

[45] Y. Qin , A. Liu , H. Guo , Y. Shen , P. Wen , H. Lin , D. Xia , M. Voshage , Y. Tian , Y. Zheng , Acta Biomater. 2022, 145, 403. 35381400 10.1016/j.actbio.2022.03.055

[46] Y. Li , P. Pavanram , J. Zhou , K. Lietaert , P. Taheri , W. Li , H. San , M. A. Leeflang , J. M. C. Mol , H. Jahr , A. A. Zadpoor , Acta Biomater. 2020, 101, 609. 31672587 10.1016/j.actbio.2019.10.034

[47] K. Buckwalter , C. Lin , J. Ford , Semin. Musculoskelet. Radiol. 2011, 15, 309. 21928156 10.1055/s-0031-1286013

[48] M. Panteli , I. Pountos , E. Jones , P. V. Giannoudis , J. Cell Mol. Med. 2015, 19, 685. 25726940 10.1111/jcmm.12532 PMC4395185

[49] S. Varma , J. P. R. O. Orgel , J. D. Schieber , Biophys. J. 2016, 111, 50. 27410733 10.1016/j.bpj.2016.05.038 PMC4945622

[50] X. Lin , S. Patil , Y.‐G. Gao , A. Qian , Front Pharmacol. 2020, 11, 757. 32528290 10.3389/fphar.2020.00757 PMC7264100

[51] M. L. Zoch , T. L. Clemens , R. C. Riddle , Bone 2016, 82, 42. 26055108 10.1016/j.bone.2015.05.046 PMC4670816

[52] B. F. Boyce , L. Xing , Arch. Biochem. Biophys. 2008, 473, 139. 18395508 10.1016/j.abb.2008.03.018 PMC2413418

[53] B. F. Boyce , L. Xing , Arthritis Res. Ther. 2007, 9 , S1. 17634140 10.1186/ar2165 PMC1924516

[54] M. J. F. Blumer , B. Hausott , C. Schwarzer , A. R. Hayman , J. Stempel , H. Fritsch , Mech. Dev. 2012, 129, 162. 22579636 10.1016/j.mod.2012.04.003 PMC3419267

[55] S. Ylä‐Herttuala , T. T. Rissanen , I. Vajanto , J. Hartikainen , J. Am. Coll Cardiol. 2007, 49, 1015. 17349880 10.1016/j.jacc.2006.09.053

[56] A. Schlüter , P. Weller , O. Kanaan , I. Nel , L. Heusgen , B. Höing , P. Haßkamp , S. Zander , M. Mandapathil , N. Dominas , J. Arnolds , B. A. Stuck , S. Lang , A. Bankfalvi , S. Brandau , BMC. Cancer 2018, 18, 272. 29523110 10.1186/s12885-018-4180-5 PMC5845191

[57] F. O. Martinez , S. Gordon , F1000Prime Rep. 2014, 6, 13. 24669294 10.12703/P6-13 PMC3944738

[58] M. H. Abdelaziz , S. F. Abdelwahab , J. Wan , W. Cai , W. Huixuan , C. Jianjun , K. D. Kumar , A. Vasudevan , A. Sadek , Z. Su , S. Wang , H. Xu , J. Transl. Med. 2020, 18, 58. 32024540 10.1186/s12967-020-02251-w PMC7003359

[59] P. J. Murray , T. A. Wynn , J. Leukoc. Biol. 2011, 89, 557. 21248152 10.1189/jlb.0710409 PMC3058818

[60] M. Wu , G. Chen , Y.‐P. Li , Bone Res. 2016, 4, 16009. 27563484 10.1038/boneres.2016.9 PMC4985055

[61] B. Osta , G. Benedetti , P. Miossec , Front Immunol. 2014, 5, 48. 24592264 10.3389/fimmu.2014.00048 PMC3923157

[62] W. Yu , H. Zhao , Z. Ding , Z. Zhang , B. Sun , J. Shen , S. Chen , B. Zhang , K. Yang , M. Liu , D. Chen , Y. He , Colloids Surf. B. Biointerfaces 2017, 149, 330. 27792982 10.1016/j.colsurfb.2016.10.037

[63] H. Hornberger , S. Virtanen , A. R. Boccaccini , Acta Biomater. 2012, 8, 2442. 22510401 10.1016/j.actbio.2012.04.012

[64] L.‐Y. Li , L.‐Y. Cui , R.‐C. Zeng , S.‐Q. Li , X.‐B. Chen , Y. Zheng , M. B. Kannan , Acta Biomater. 2018, 79, 23. 30149212 10.1016/j.actbio.2018.08.030

[65] H. Guo , D. Xia , Y. Zheng , Y. Zhu , Y. Liu , Y. Zhou , Acta Biomater. 2020, 106, 396. 32092431 10.1016/j.actbio.2020.02.024

[66] B. Wang , Y. Li , S. Wang , F. Jia , A. Bian , K. Wang , L. Xie , K. Yan , H. Qiao , H. Lin , J. Lan , Y. Huang , Mater. Sci. Eng., C 2021, 129, 112387. 10.1016/j.msec.2021.112387 34579906

[67] J. Katouno , K. Fujioka , S. Kidera , Y. Mabuchi , K. Sato , Y. Ohgoe , Y. Manome , M. Hiratsuka , H. Nakamori , H. Masuda , H. Honda , K. Hirakuri , Diamond Relat. Mater. 2017, 77, 131.

[68] W. Song , J. Zhang , J. Guo , J. Zhang , F. Ding , L. Li , Z. Sun , Toxicol. Lett. 2010, 199, 389. 20934491 10.1016/j.toxlet.2010.10.003

[69] R. Wu , Y. Li , M. Shen , X. Yang , L. Zhang , X. Ke , G. Yang , C. Gao , Z. Gou , S. Xu , Bioact Mater 2021, 6, 1242. 33210022 10.1016/j.bioactmat.2020.11.003 PMC7653208

[70] A. Entezari , I. Roohani , G. Li , C. R. Dunstan , P. Rognon , Q. Li , X. Jiang , H. Zreiqat , Adv. Healthc. Mater. 2019, 8, 1801353. 10.1002/adhm.201801353 30536610

[71] P. R. Buenzli , M. Lanaro , C. S. Wong , M. P. Mclaughlin , M. C. Allenby , M. A. Woodruff , M. J. Simpson , Acta Biomater. 2020, 114, 285. 32673750 10.1016/j.actbio.2020.07.010

[72] J. Zhang , Y. Jiang , Z. Shang , B. Zhao , M. Jiao , W. Liu , M. Cheng , B. Zhai , Y. Guo , B. Liu , X. Shi , B. Ma , Bioactive Mater. 2021, 6, 4027. 10.1016/j.bioactmat.2021.03.035 PMC8089787 33997491

[73] X. Liu , J. Sun , F. Zhou , Y. Yang , R. Chang , K. Qiu , Z. Pu , L. Li , Y. Zheng , Mater. Des. 2016, 94, 95.

[74] B. Jia , H. Yang , Y. Han , Z. Zhang , X. Qu , Y. Zhuang , Q. Wu , Y. Zheng , K. Dai , Acta Biomater. 2020, 108, 358. 32165194 10.1016/j.actbio.2020.03.009

[75] H. F. Li , X. H. Xie , Y. F. Zheng , Y. Cong , F. Y. Zhou , K. J. Qiu , X. Wang , S. H. Chen , L. Huang , L. Tian , L. Qin , Sci. Rep. 2015, 5, 10719. 26023878 10.1038/srep10719 PMC4448657

[76] Y. Yu , G. Jin , Y. Xue , D. Wang , X. Liu , J. Sun , Acta Biomater. 2017, 49, 590. 27915020 10.1016/j.actbio.2016.11.067

[77] S. A. Sreenivasamurthy , F. F. Akhter , A. Akhter , Y. Su , D. Zhu , Biomater. Adv. 2022, 139, 213023. 35882117 10.1016/j.bioadv.2022.213023

[78] E. Oohashi , Y. Kimura , K. Matsumoto , Vet. Rec. Open 2019, 6, e000272. 31565226 10.1136/vetreco-2017-000272 PMC6746537

[79] Y. Yao , X. Cai , F. Ren , Y. Ye , F. Wang , C. Zheng , Y. Qian , M. Zhang , Front Immunol. 2021, 12, 664871. 33868316 10.3389/fimmu.2021.664871 PMC8044404

[80] N. Lampiasi , R. Russo , F. Zito , Biomed Res. Int. 2016, 2016, 9089610. 26977415 10.1155/2016/9089610 PMC4761668

[81] S. L. Stafford , N. J. Bokil , M. E. S. Achard , R. Kapetanovic , M. A. Schembri , A. G. Mcewan , M. J. Sweet , Biosci. Rep. 2013, 33, e00049. 23738776 10.1042/BSR20130014 PMC3712485

[82] H. Gao , W. Dai , L. Zhao , J. Min , F. Wang , J. Immunol. Res. 2018, 2018, 6872621. 30622979 10.1155/2018/6872621 PMC6304900

[83] F. Benn , N. Kröger , M. Zinser , K. Van Gaalen , T. J. Vaughan , M. Yan , R. Smeets , E. Bibiza , S. Malinov , F. Buchanan , A. Kopp , Mater. Sci. Eng. C. Mater. Biol. Appl. 2021, 124, 112016. 33947530 10.1016/j.msec.2021.112016

[84] A. Crowe , W. Yue , Bio‐protocol 2019, 9, e3465. 31867411 10.21769/BioProtoc.3465 PMC6924920

[85] N. Fedchenko , J. Reifenrath , Diagn. Pathol. 2014, 9, 1. 25432701 10.1186/s13000-014-0221-9 PMC4260254

