# Supplementary material for: Evolution from Bioinert to Bioresorbable: In Vivo Comparative Study of Additively Manufactured Metal Bone Scaffolds
Source: Adv Sci (Weinh). 2023 Jul 10;10(26):2302702. doi: 10.1002/advs.202302702 (PMC10502659; doi:10.1002/advs.202302702)
Supplement: Supplementary file 1 — Supporting Information [file ADVS-10-2302702-s001.pdf]

ADVANCED SCIENCE
Open Access

Supporting Information

for *Adv. Sci.*, DOI 10.1002/advs.202302702

Evolution from Bioinert to Bioresorbable: In Vivo Comparative Study of Additively Manufactured Metal Bone Scaffolds

*Juncen Zhou, Elias Georgas, Yingchao Su, Jiayi Zhou, Nadja Kröger, Felix Benn, Alexander Kopp, Yi-Xian Qin and Donghui Zhu**

Supplementary Materials for

Evolution from Bioinert to Bioresorbable: In Vivo Comparative Study of Additively Manufactured Metal Bone Scaffolds

Authors: Juncen Zhou[1], Elias Georgas[1], Yingchao Su[1], Jiayi Zhou[1], Nadja Kröger[2], Felix Benn[3], Alexander Kopp[3], Yixian Qin[1], Donghui Zhu[1]*

*Corresponding author. Email: donghui.zhu@stonybrook.edu

The PDF file includes:

Fig. S1. The architectural structure of scaffolds.

Fig. S2. The microstructure of scaffolds.

Fig. S3. H&E staining of organs.

Fig. S4. Additional information related to the degradation behavior of Mg scaffolds.

Fig. S5. Additional information related to the degradation behavior of Zn scaffolds.

Fig. S6. Additional information related to the degradation behavior of Ti scaffolds.

Fig. S7. Representative μCT scan images.

Fig. S8. Bone regeneration inside Mg scaffolds.

Fig. S9. Additional staining images of side and marrow regions of scaffolds.

Fig. S10. Additional information related to the osteoid tissue inside Zn scaffolds.

Fig. S11. Additional TRAP staining images.

Fig. S12. IHC images of BMP-2.

Fig. S13. IHC images of collagen I.

Fig. S14. IHC images of osteocalcin.

Fig. S15. IHC images of Osteoprotegerin (OPG).

Fig. S16. IHC images of Receptor activator of nuclear factor kappa-B ligand (RANKL).

Fig. S17. IHC images of acid phosphatase 5 (ACP5).

Fig. S18. IHC images of cluster of differentiation 31 (CD31).

Fig. S19. IHC images of vascular endothelial growth factor (VEGF).

Fig. S20. IHC images of cluster of differentiation 68 (CD68).

Fig. S21. IHC images of tumor necrosis factor alpha (TNF-α).

Fig. S22. IHC images of transglutaminase 2 (TGM2).

Fig. S23. IHC images of Transforming growth factor-beta-1,2,3 (TGF-β 123).

Fig. S24. Additional biomechanical tests.

Fig.S25. Von Mises stress distribution on scaffolds.

Fig.S26. Von Mises stress distribution on the front bone.

Fig. S27. Staining images showing the interface of osteoid in the front region of Zn scaffolds.

Fig. S28. CRP level in serum.

Table S1. Blood chemistry panel of serum.

Table S2. Scoring system used for semi-quantification IHC evaluation.

Table S3. Summary of semi-quantification of IHC evaluation (Exile file).

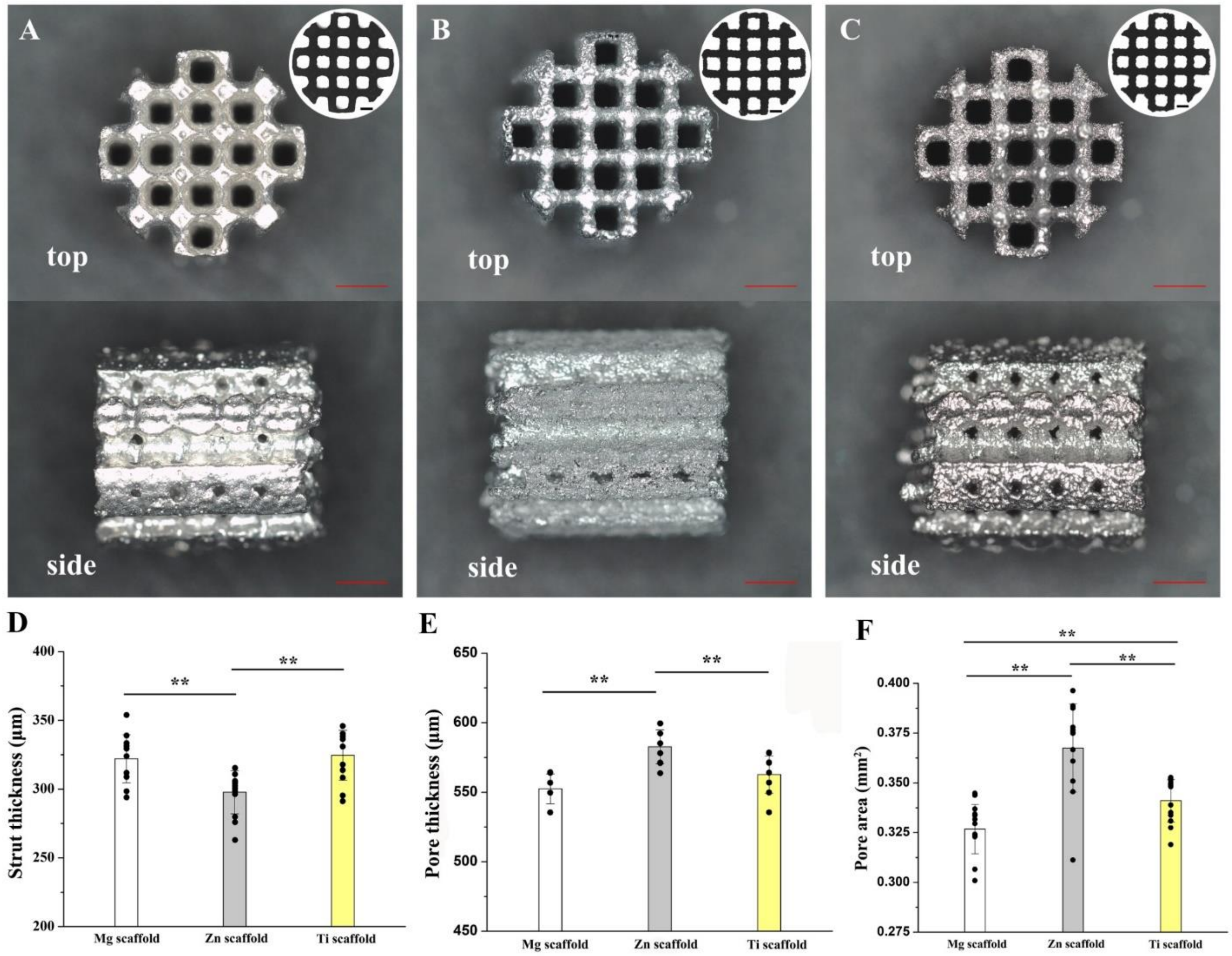

Fig. S1. The architectural structure of scaffolds. Optical images and morphological analysis of 3D-printed Mg, Zn, and Ti scaffolds. (A) Mg scaffold, (B) Zn scaffold, and (C) Ti scaffold are shown in both top and side views, with a red bar of 1 mm. Insets depict the boundaries of struts and pores, with a black bar of 0.5 mm. (D) The strut thickness, (E) the pore size, and (F) the pore area of the scaffolds were analyzed.

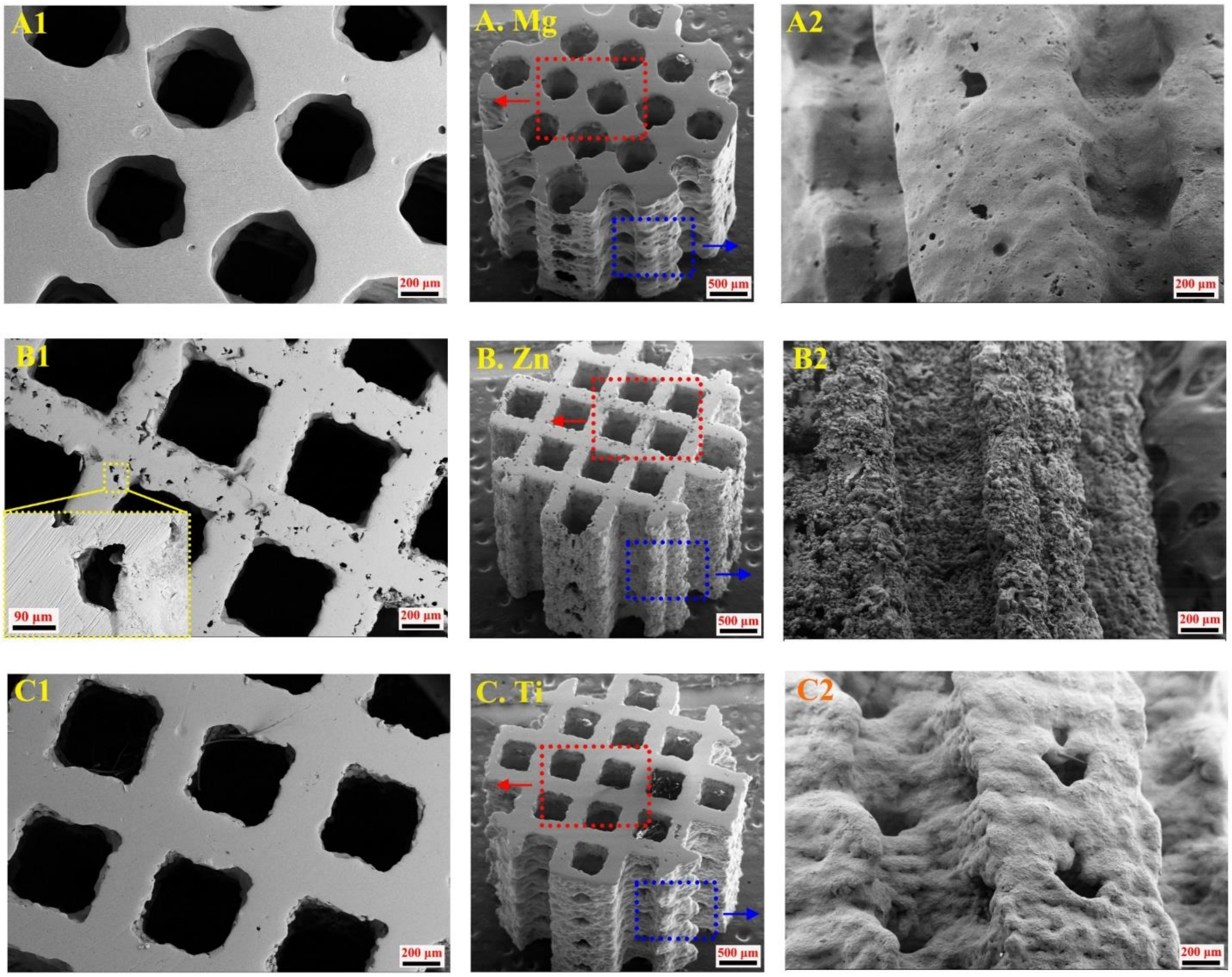

Fig. S2. The microstructure of scaffolds. SEM images of (A) Mg scaffold, (B) Zn scaffold, and (C) Ti scaffold, showing the polished top surfaces in the left column and the as-printed side surfaces in the right column.

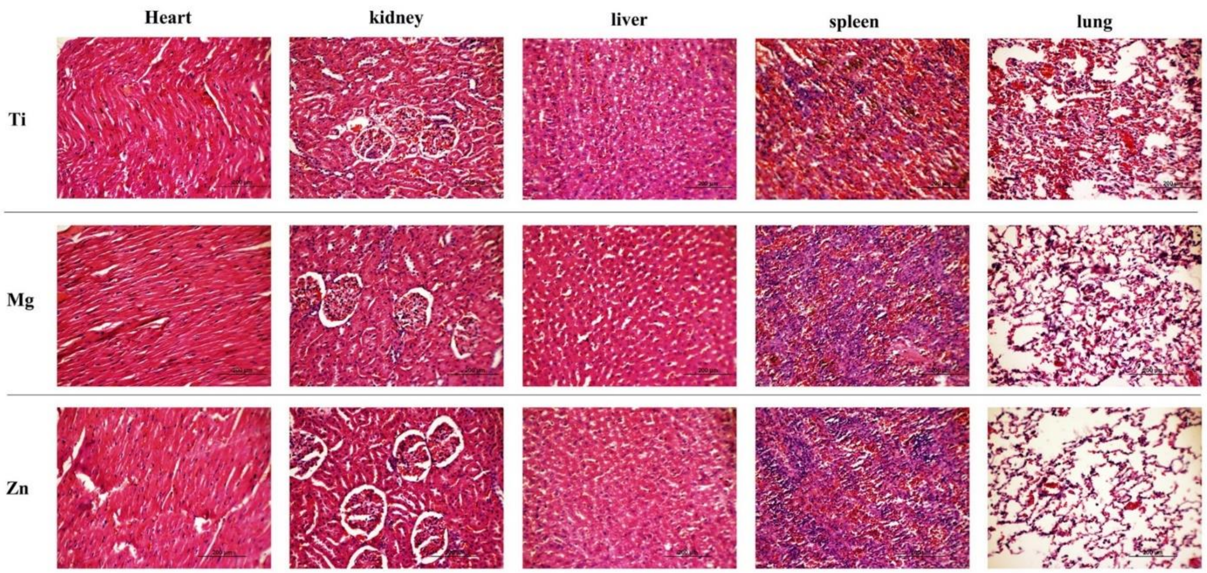

Fig. S3. H&E staining of organs. Representative H&E staining images of organs including heart, kidney, liver, spleen, and lung collected at week 25 after implantation of Ti, Mg, and Zn scaffolds. No significant differences were observed among the groups.

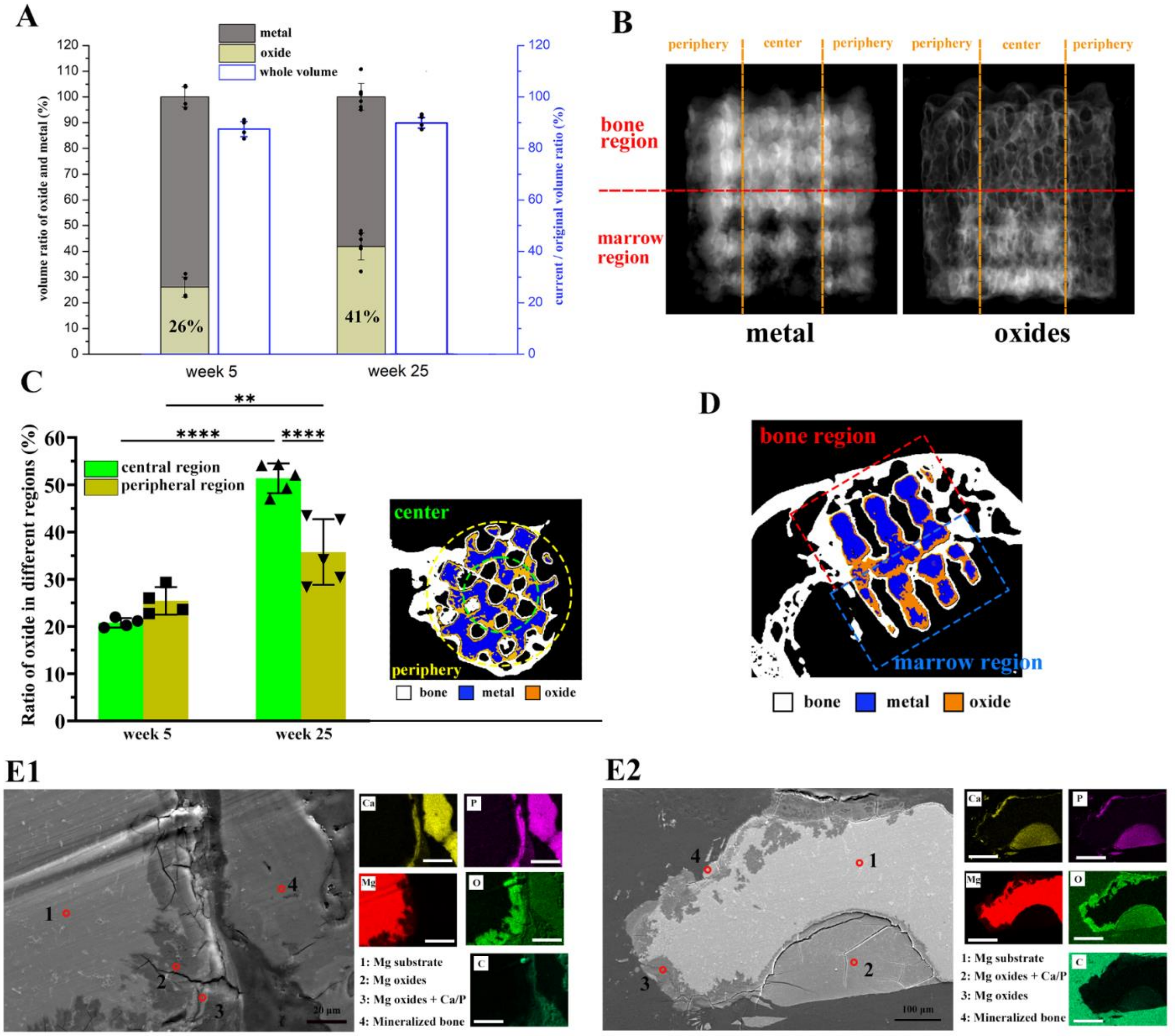

Fig. S4. Additional information related to the degradation behavior of Mg scaffolds. (A) The volume change of the whole Mg scaffold as derived from μCT results. The blue bar represents the entire volume of the scaffold, including Mg metal and Mg oxides (degradation products). The grey bar represents the volume ratio of Mg metal, and the brown bar represents the volume ratio of Mg oxides. (B) X-ray model images of a 25-week-implanted Mg scaffold derived from μCT results. The left image shows Mg metal, while the right image shows oxides. (C-D) The distribution of Mg oxides in different regions in week 25. The ratio of oxides in the center and periphery regions in the frontal plane is shown in (C), while the bone and marrow regions in the transverse plane are shown in (D). SEM images and EDS/Elemental mapping of a week 5-implanted Mg scaffold in the bone region (E1) and marrow region (E2).

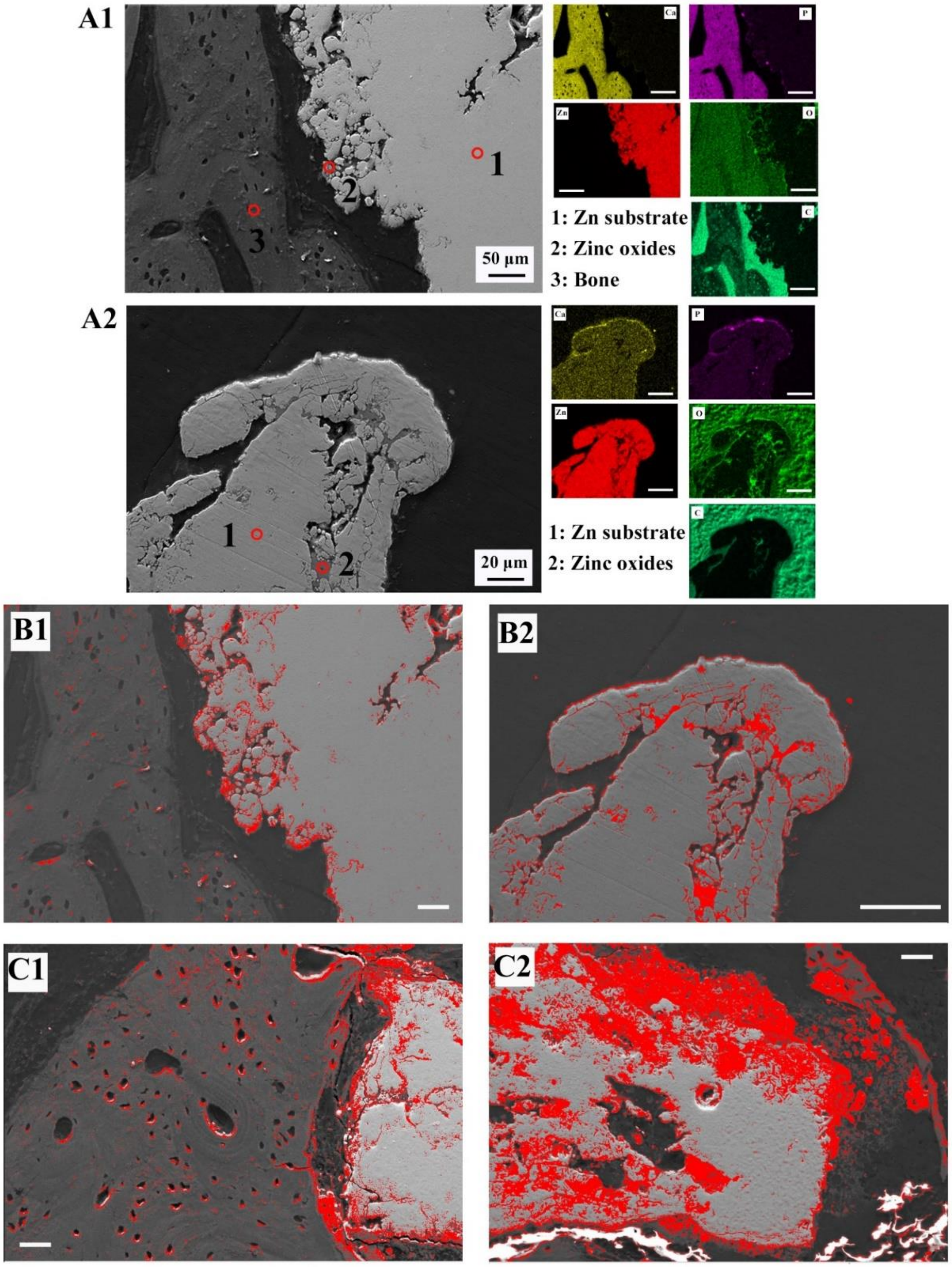

Fig. S5. Additional information related to the degradation behavior of Zn scaffolds. SEM images and EDS/Elemental mapping of Zn scaffolds implanted for 5 weeks in the bone region (A1) and marrow region (A2) are presented. SEM images of bone (B1) and marrow (B2) regions of 5-week implanted Zn scaffold, and bone (C1) and marrow (C2) regions of 25-week implanted Zn scaffold are also shown. Zinc oxides are marked in red. White scale bar is 20 µm.

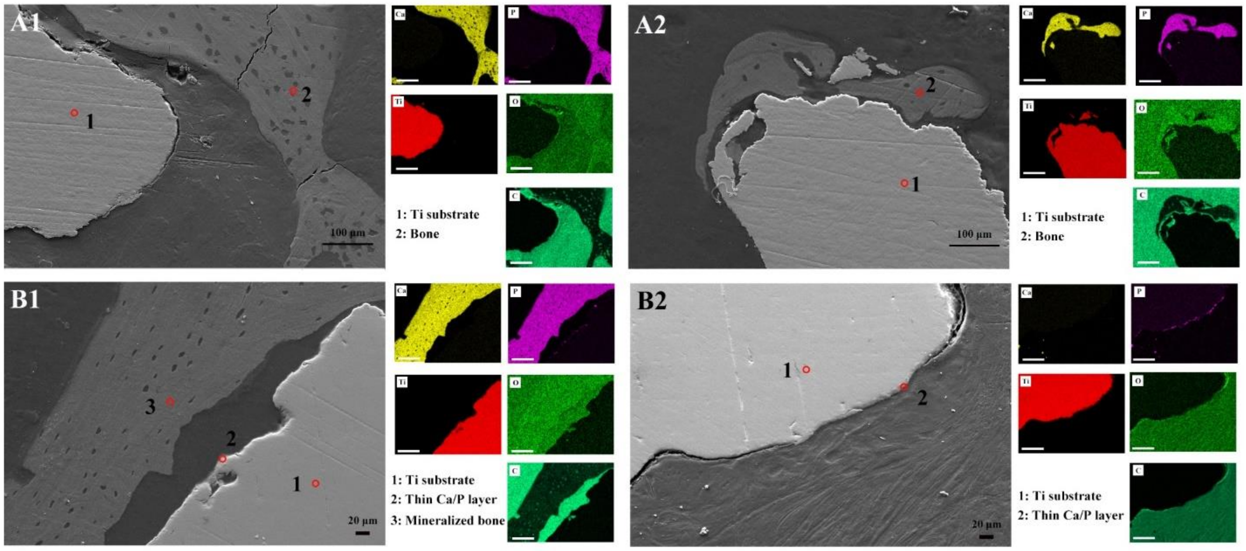

Fig. S6. Additional information related to the degradation behavior of Ti scaffolds. SEM images and EDS/Elemental mapping of (A1) bone region and (A2) marrow region in week 5-implanted Ti scaffold, and (B1) bone region and (B2) marrow region in week 25-implanted Ti scaffold. White scale bar: 200 μm.

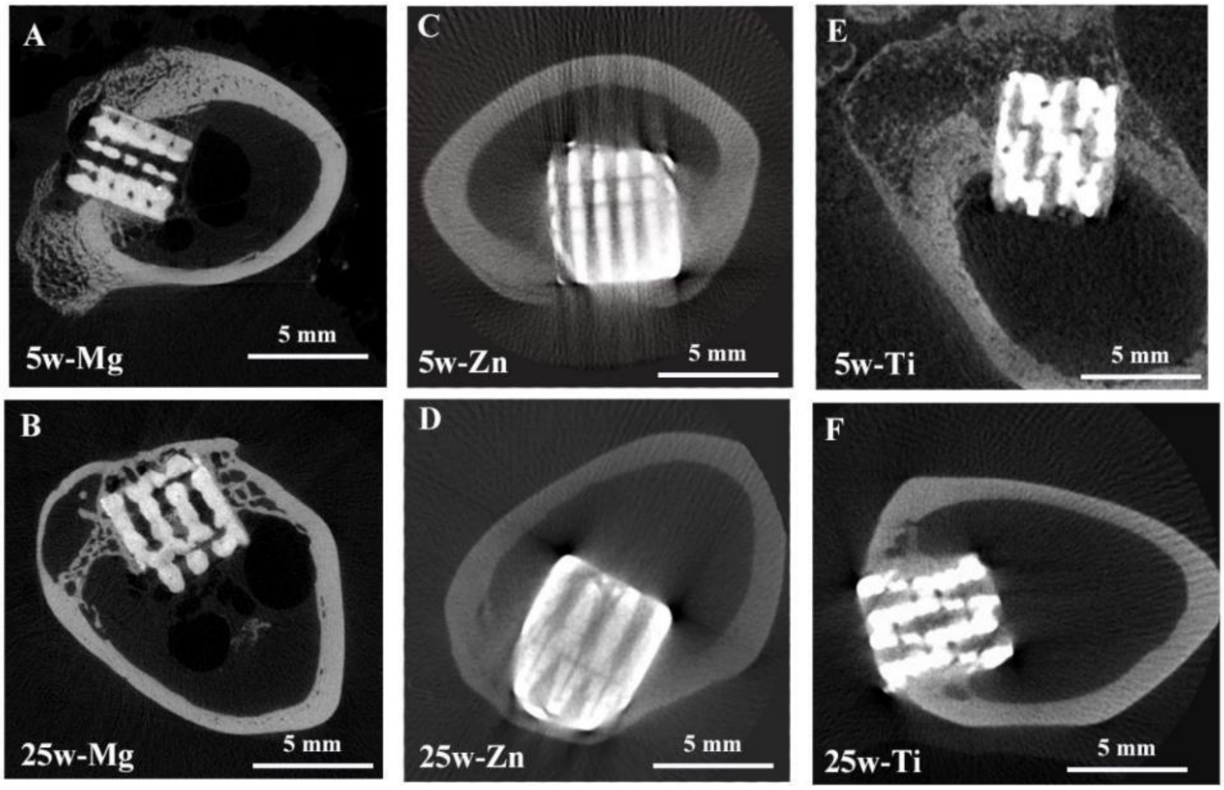

Fig. S7. Representative μCT scan images. Representative transverse plane μCT scan images of Mg scaffold at 5 (A) and 25 (B) weeks, Zn scaffold at 5 (C) and 25 (D) weeks, and Ti scaffold at 5 (E) and 25 (F) weeks after implantation.

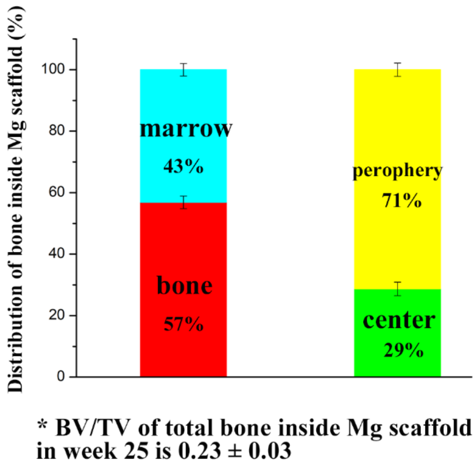

Fig. S8. Bone regeneration inside Mg scaffolds. The distribution of newly formed bone within the Mg scaffold after 25 weeks of implantation in the transverse and frontal planes, derived from μCT scan results. The bone volume fraction (BV/TV) within the entire Mg scaffold was found to be 0.23±0.03 in week 25. In contrast, no significant bone formation was observed within the Mg-5w, Zn-5w, Zn-25w, Ti-5w, or Ti-25w scaffolds.

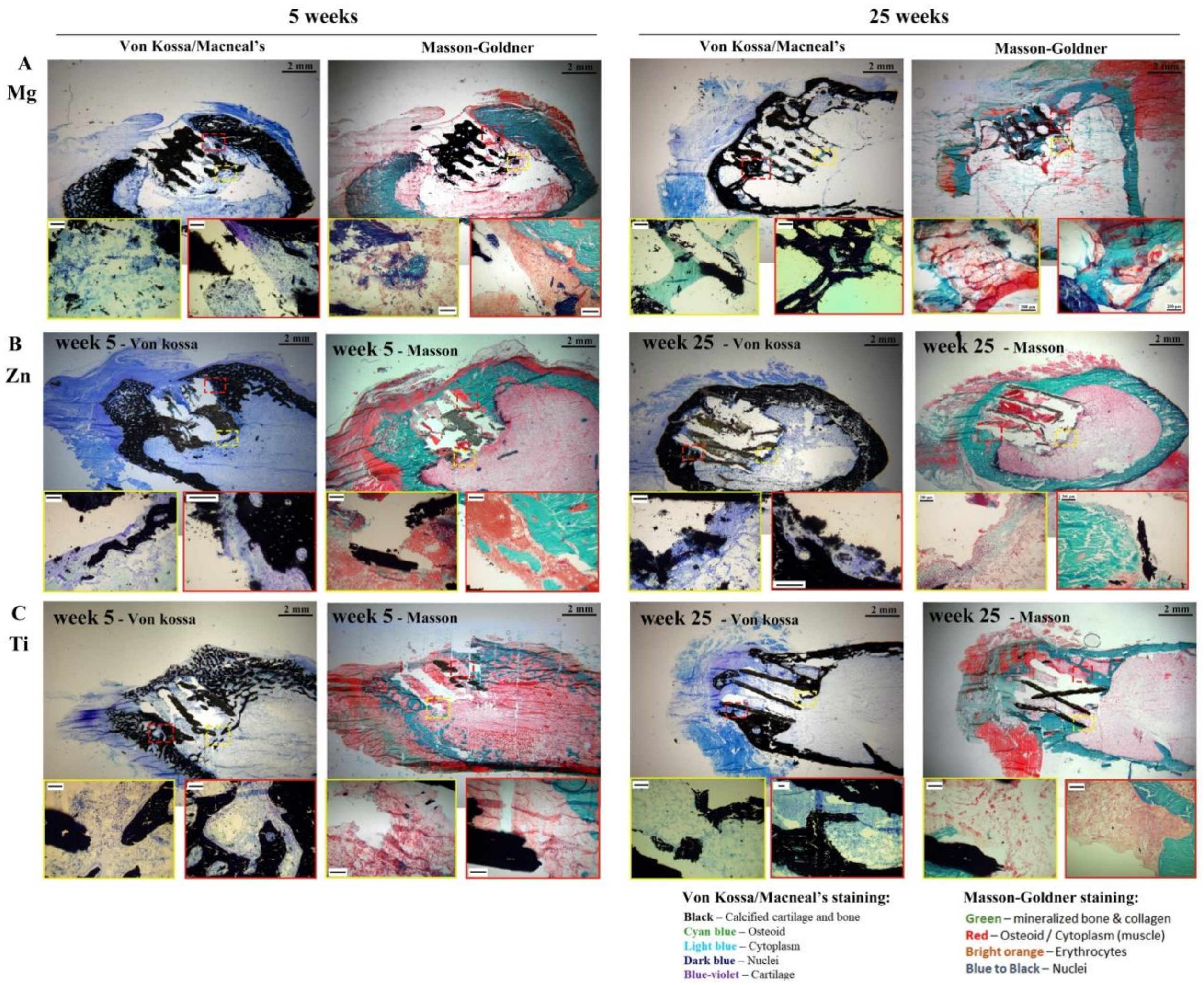

Fig. S9. Additional staining images of side and marrow regions of scaffolds. Representative Von Kossa/Macneal's and Masson-Goldner stained histological images of (A) Mg scaffold, (B) Zn scaffold, (C) Ti scaffold, and (D) sham control after 5 and 25 weeks of implantation. The yellow box highlights the detailed histological structure in the marrow region of the scaffold, and the red box highlights the side region. Black scale bar: 200 μm.

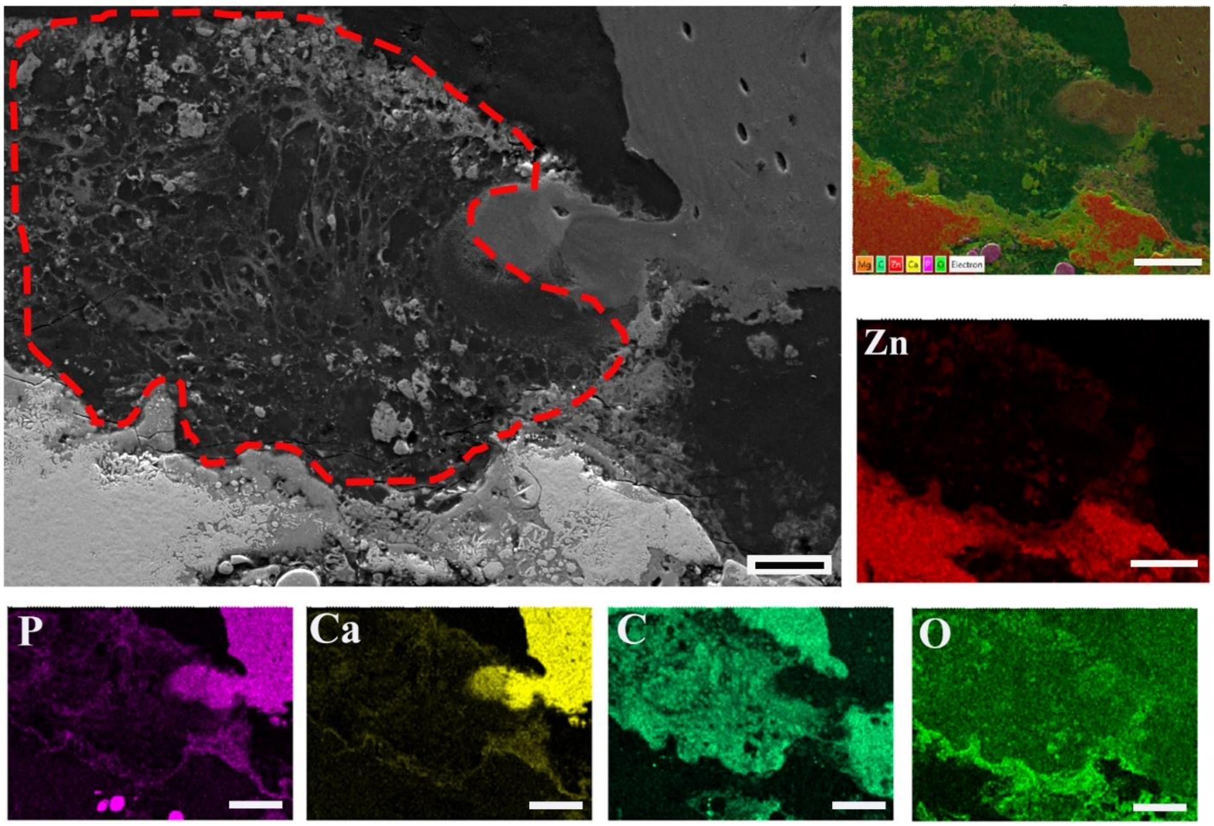

Fig. S10. Additional information related to the osteoid tissue inside Zn scaffolds. SEM and EDS/Elemental mapping images of the inside region in Zn scaffold after 25 weeks of implantation, highlighting the osteoid tissue with a red dotted line. Notably, a significant amount of Zn element can be seen in the osteoid tissue. Black scale bar: 40 μm, white scale bar: 100 μm.

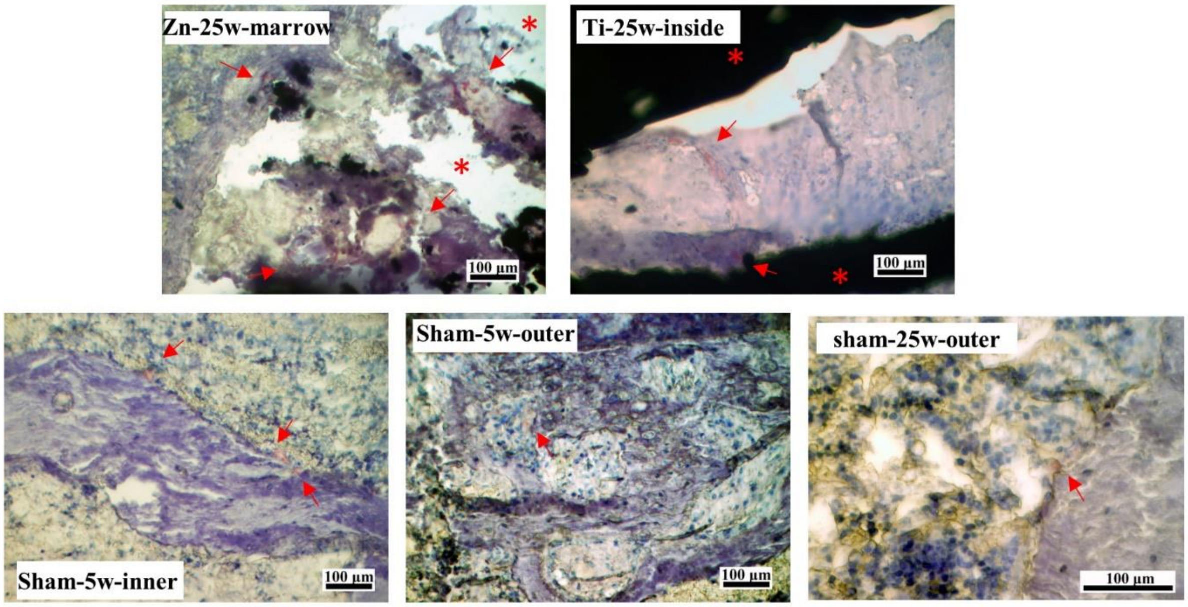

Fig. S11. Additional TRAP staining images. Representative TRAP staining images of the marrow and inside regions of scaffolds, as well as the inner and outer bone layers of the sham control group. The location of the scaffold is indicated by *, while spots with positive TRAP staining are marked by →.

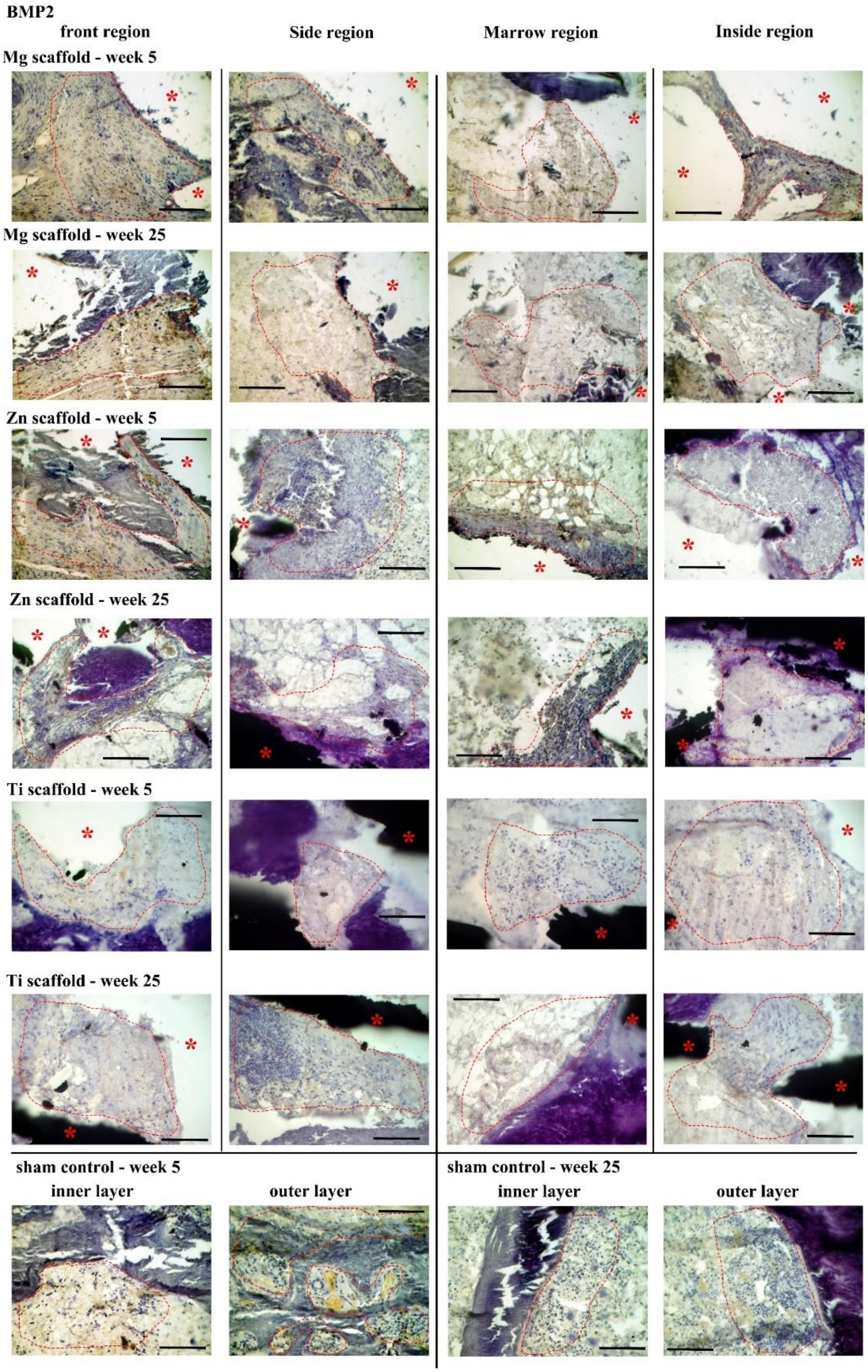

Fig. S12. IHC images of BMP-2. Representative images of BMP-2 marker expression from Mg-5w, Mg-25w, Zn-5w, Zn-25w, Ti-5w, Ti-25w, sham-5w, and sham-25w groups. Images were captured from the front, side, marrow, and inside regions of the scaffold, and the inner and outer bone layers for the sham control groups. The location of the scaffold is indicated by *. The red dotted line represents the region of interest (ROI) for marker expression quantification. Black scale bar: 100 μm.

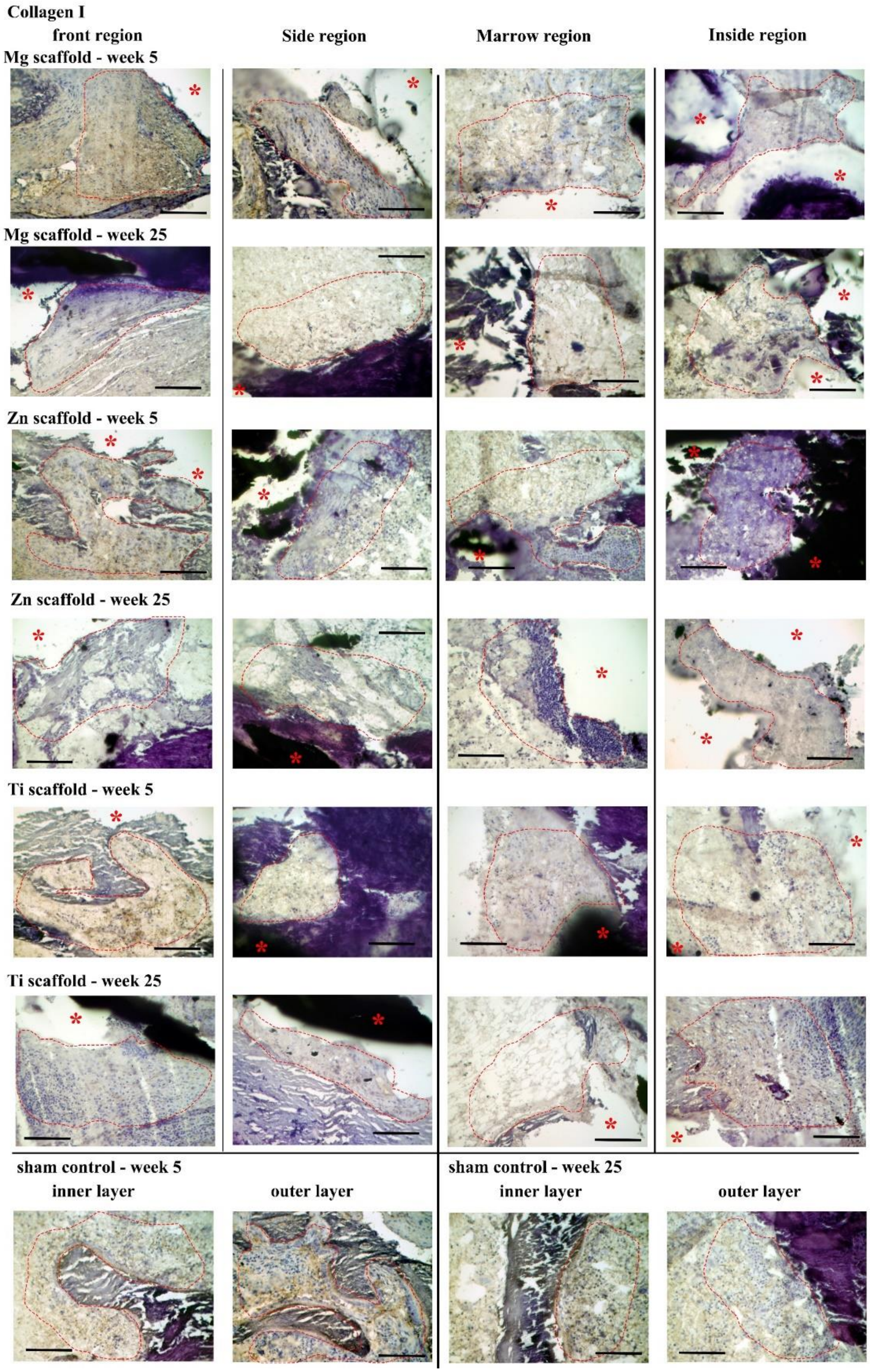

Fig. S13. IHC images of collagen I. Representative images of collagen I marker expression from Mg-5w, Mg-25w, Zn-5w, Zn-25w, Ti-5w, Ti-25w, sham-5w, and sham-25w groups. Images were captured from the front, side, marrow, and inside regions of the scaffold, and the inner and outer bone layers for the sham control groups. The location of the scaffold is indicated by *. The red dotted line represents the region of interest (ROI) for marker expression quantification. Black scale bar: 100 µm.

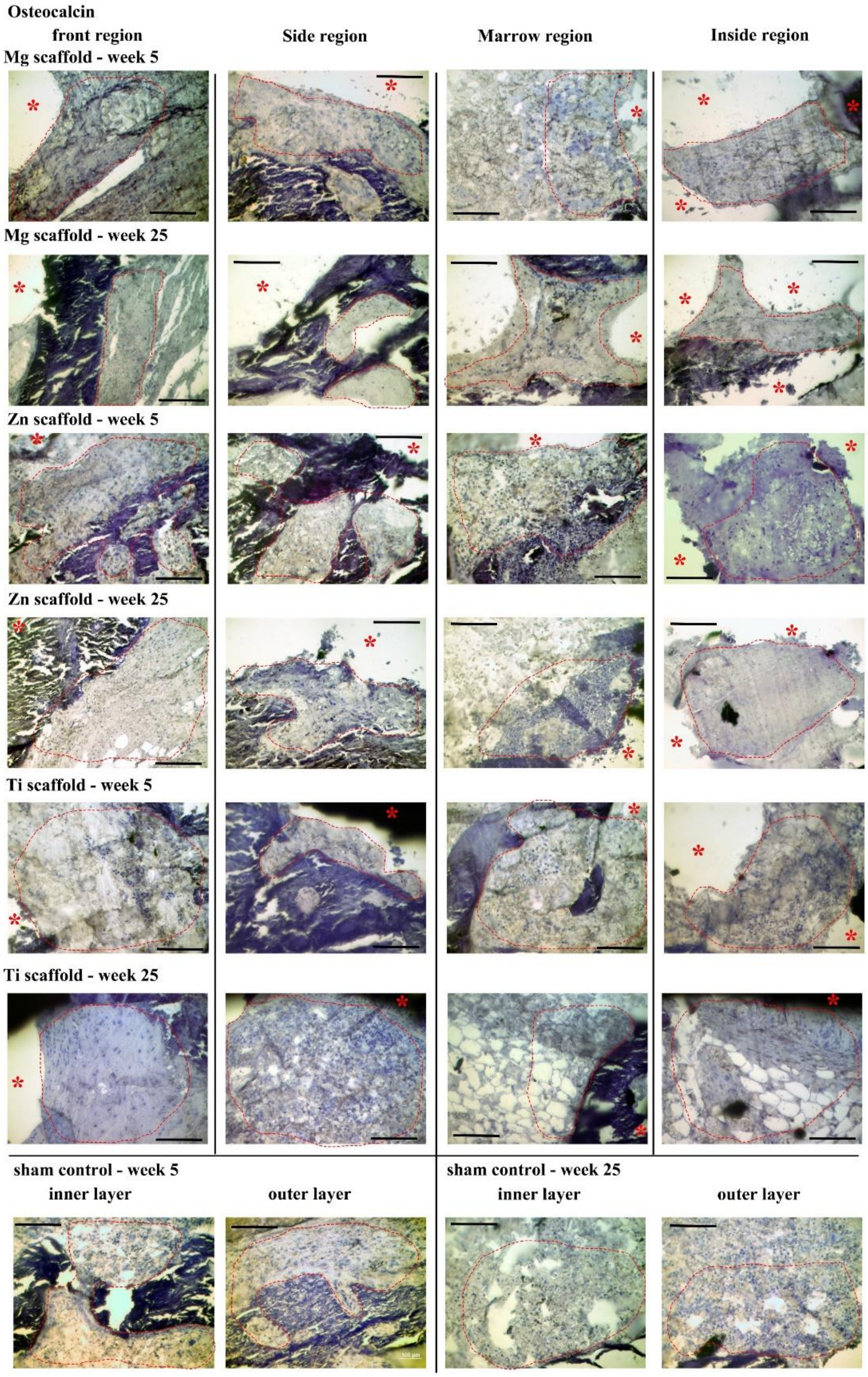

Fig. S14. IHC images of osteocalcin. Representative images of osteocalcin marker expression from Mg-5w, Mg-25w, Zn-5w, Zn-25w, Ti-5w, Ti-25w, sham-5w, and sham-25w groups. Images were captured from the front, side, marrow, and inside regions of the scaffold, and the inner and outer bone layers for the sham control groups. The location of the scaffold is indicated by *. The red dotted line represents the region of interest (ROI) for marker expression quantification. Black scale bar: 100 µm.

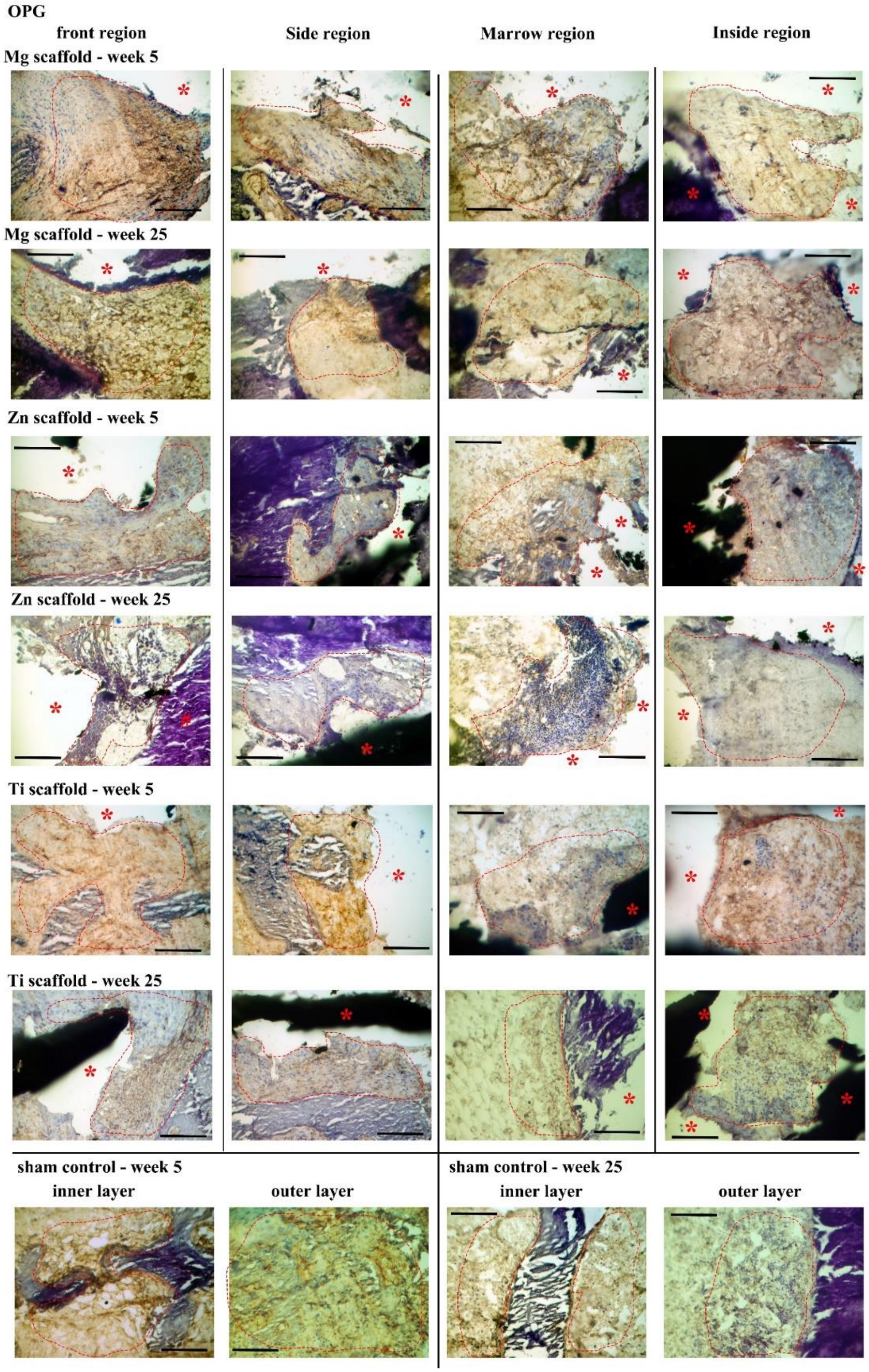

Fig. S15. IHC images of Osteoprotegerin (OPG). Representative images of OPG marker expression from Mg-5w, Mg-25w, Zn-5w, Zn-25w, Ti-5w, Ti-25w, sham-5w, and sham-25w groups. Images were captured from the front, side, marrow, and inside regions of the scaffold, and the inner and outer bone layers for the sham control groups. The location of the scaffold is indicated by *. The red dotted line represents the region of interest (ROI) for marker expression quantification. Black scale bar: 100 µm.

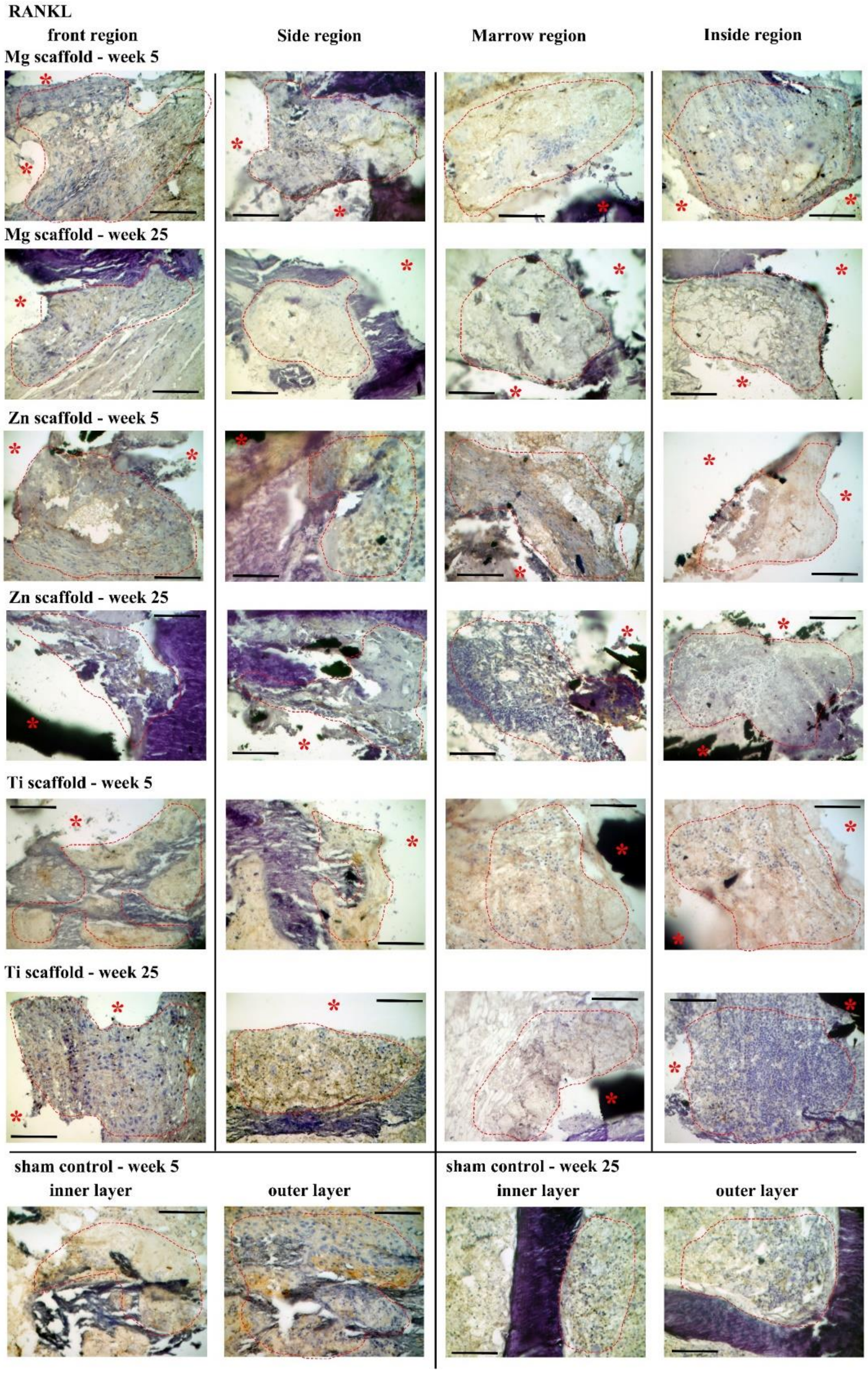

Fig. S16. IHC images of Receptor activator of nuclear factor kappa-B ligand (RANKL). Representative images of RANKL marker expression from Mg-5w, Mg-25w, Zn-5w, Zn-25w, Ti-5w, Ti-25w, sham-5w, and sham-25w groups. Images were captured from the front, side, marrow, and inside regions of the scaffold, and the inner and outer bone layers for the sham control groups. The location of the scaffold is indicated by *. The red dotted line represents the region of interest (ROI) for marker expression quantification. Black scale bar: 100 μm.

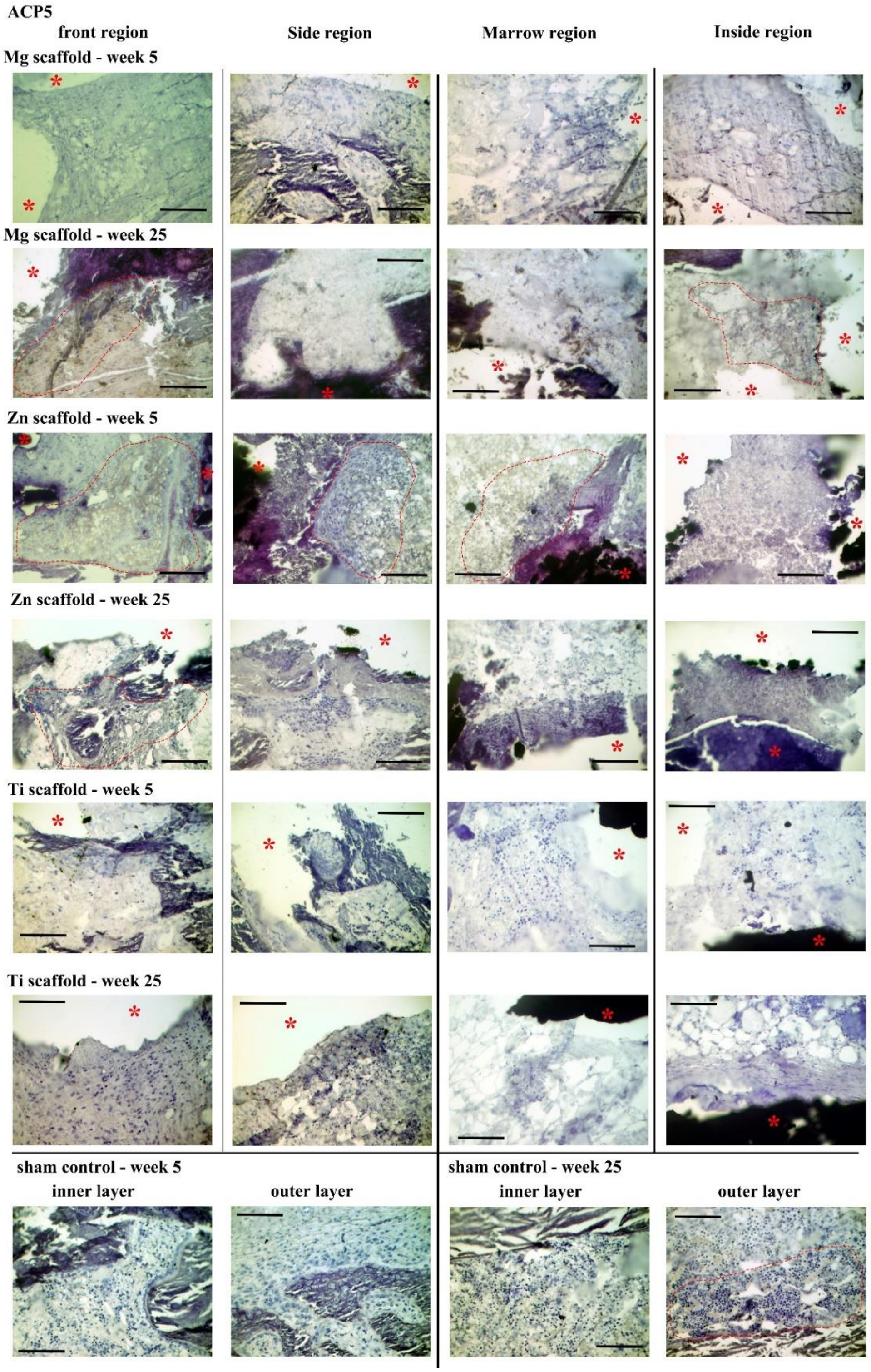

Fig. S17. IHC images of acid phosphatase 5 (ACP5). Representative images of ACP5 marker expression from Mg-5w, Mg-25w, Zn-5w, Zn-25w, Ti-5w, Ti-25w, sham-5w, and sham-25w groups. Images were captured from the front, side, marrow, and inside regions of the scaffold, and the inner and outer bone layers for the sham control groups. The location of the scaffold is indicated by *. The red

dotted line represents the region of interest (ROI) for marker expression quantification. Black scale bar: 100 μm.

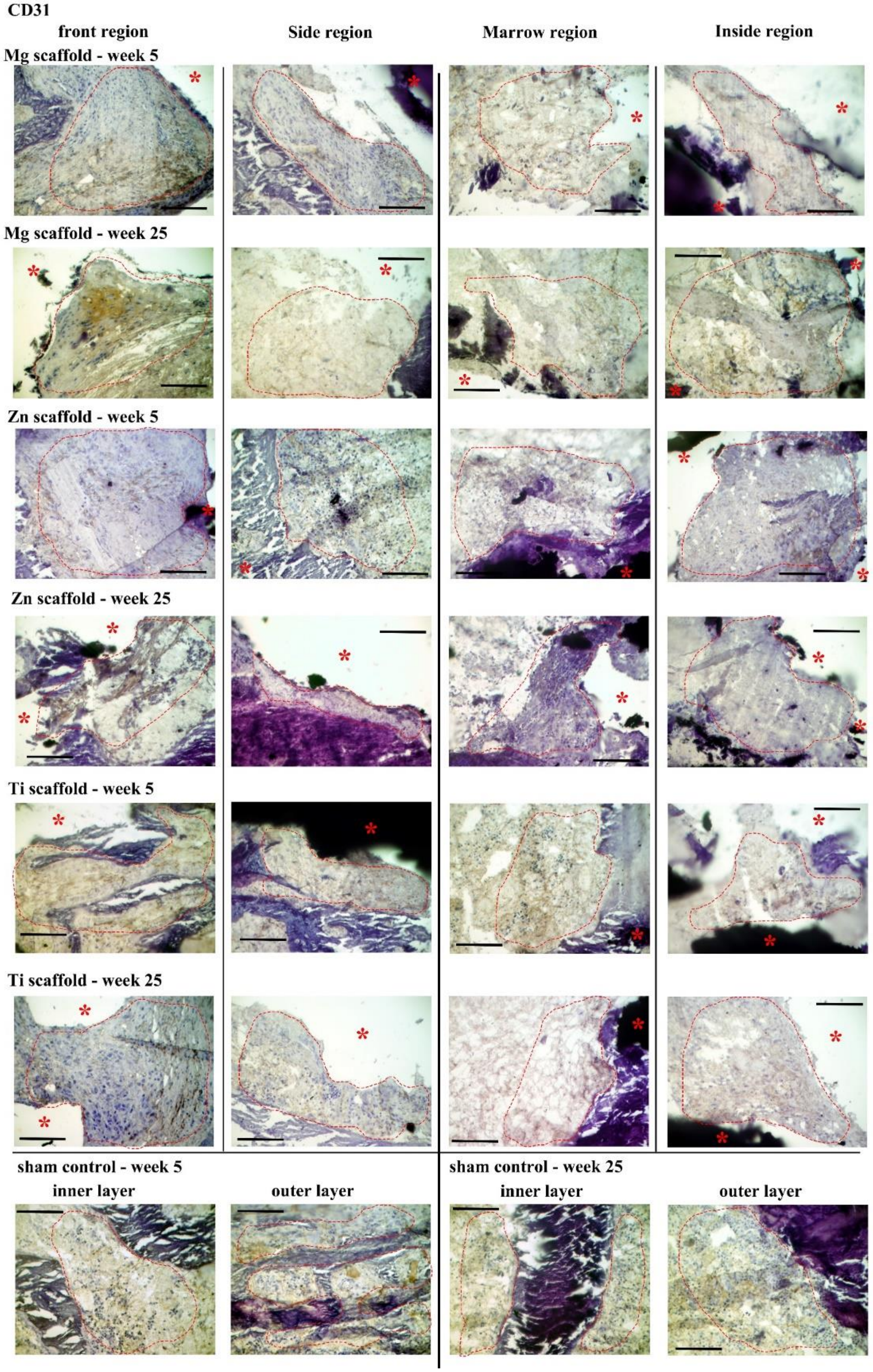

Fig. S18. IHC images of cluster of differentiation 31 (CD31). Representative images of CD31 marker expression from Mg-5w, Mg-25w, Zn-5w, Zn-25w, Ti-5w, Ti-25w, sham-5w, and sham-25w groups.

Images were captured from the front, side, marrow, and inside regions of the scaffold, and the inner and outer bone layers for the sham control groups. The location of the scaffold is indicated by *. The red dotted line represents the region of interest (ROI) for marker expression quantification. Black scale bar: 100 μm.

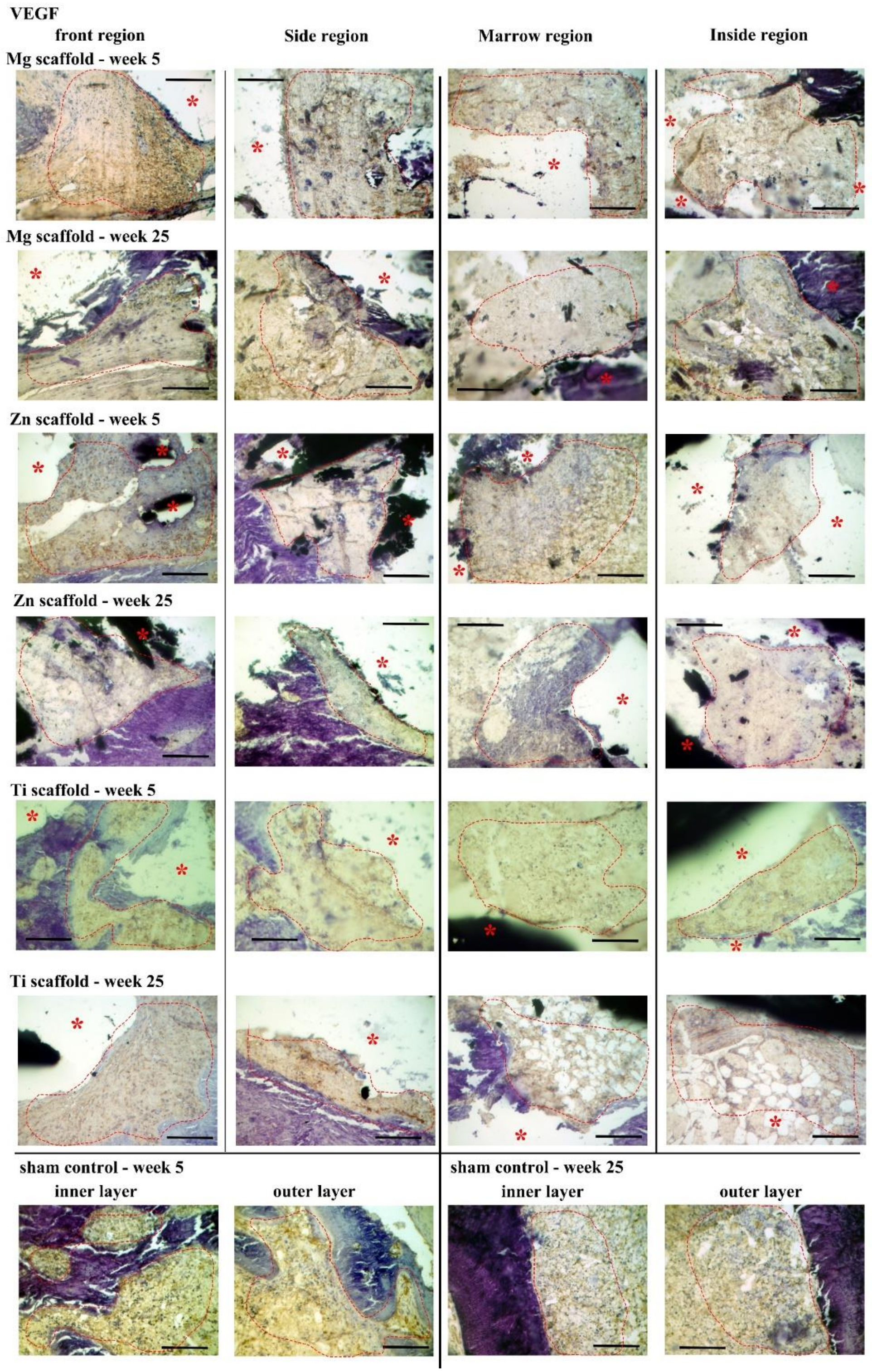

Fig. S19. IHC images of vascular endothelial growth factor (VEGF). Representative images of VEGF marker expression from Mg-5w, Mg-25w, Zn-5w, Zn-25w, Ti-5w, Ti-25w, sham-5w, and sham-25w groups. Images were captured from the front, side, marrow, and inside regions of the scaffold, and the inner and outer bone layers for the sham control groups. The location of the scaffold is indicated by *. The red dotted line represents the region of interest (ROI) for marker expression quantification. Black scale bar: 100 μm.

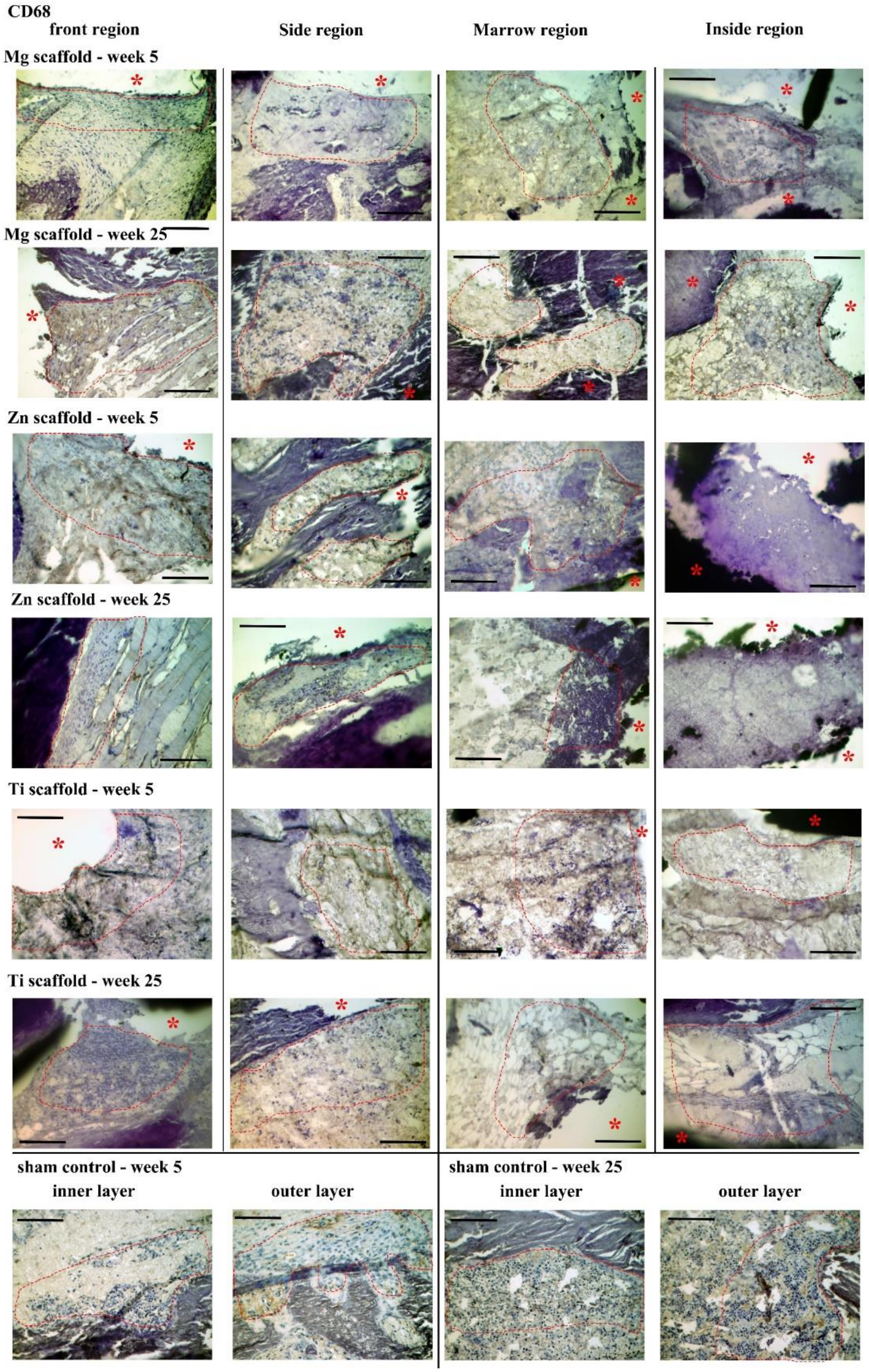

Fig. S20. IHC images of cluster of differentiation 68 (CD68). Representative images of CD68 marker expression from Mg-5w, Mg-25w, Zn-5w, Zn-25w, Ti-5w, Ti-25w, sham-5w, and sham-25w groups. Images were captured from the front, side, marrow, and inside regions of the scaffold, and the inner and outer bone layers for the sham control groups. The location of the scaffold is indicated by *. The red

dotted line represents the region of interest (ROI) for marker expression quantification. Black scale bar: 100 μm.

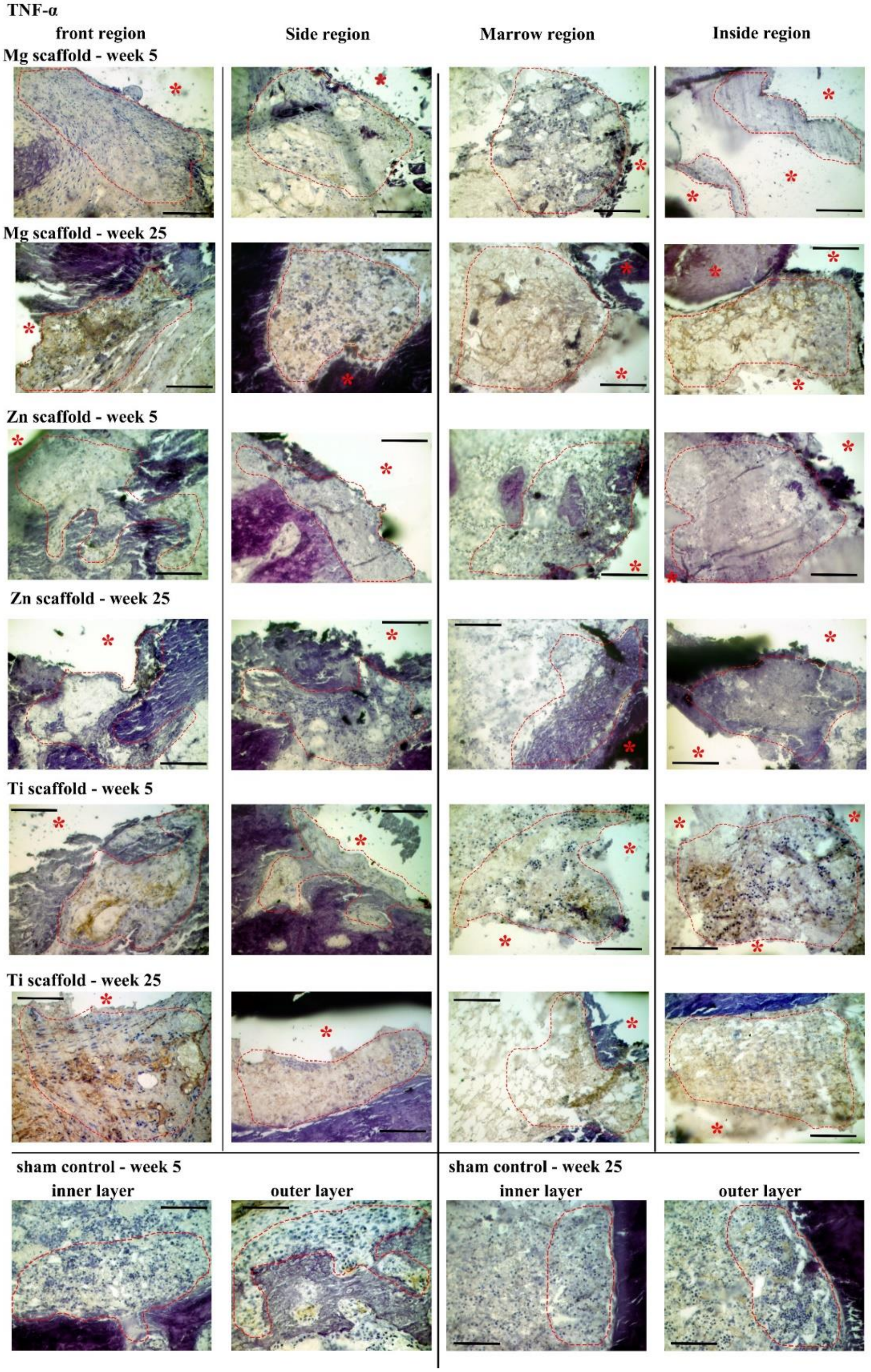

Fig. S21. IHC images of tumor necrosis factor alpha (TNF-α). Representative images of TNF-α marker expression from Mg-5w, Mg-25w, Zn-5w, Zn-25w, Ti-5w, Ti-25w, sham-5w, and sham-25w groups. Images were captured from the front, side, marrow, and inside regions of the scaffold, and the

inner and outer bone layers for the sham control groups. The location of the scaffold is indicated by *. The red dotted line represents the region of interest (ROI) for marker expression quantification. Black scale bar: 100 µm.

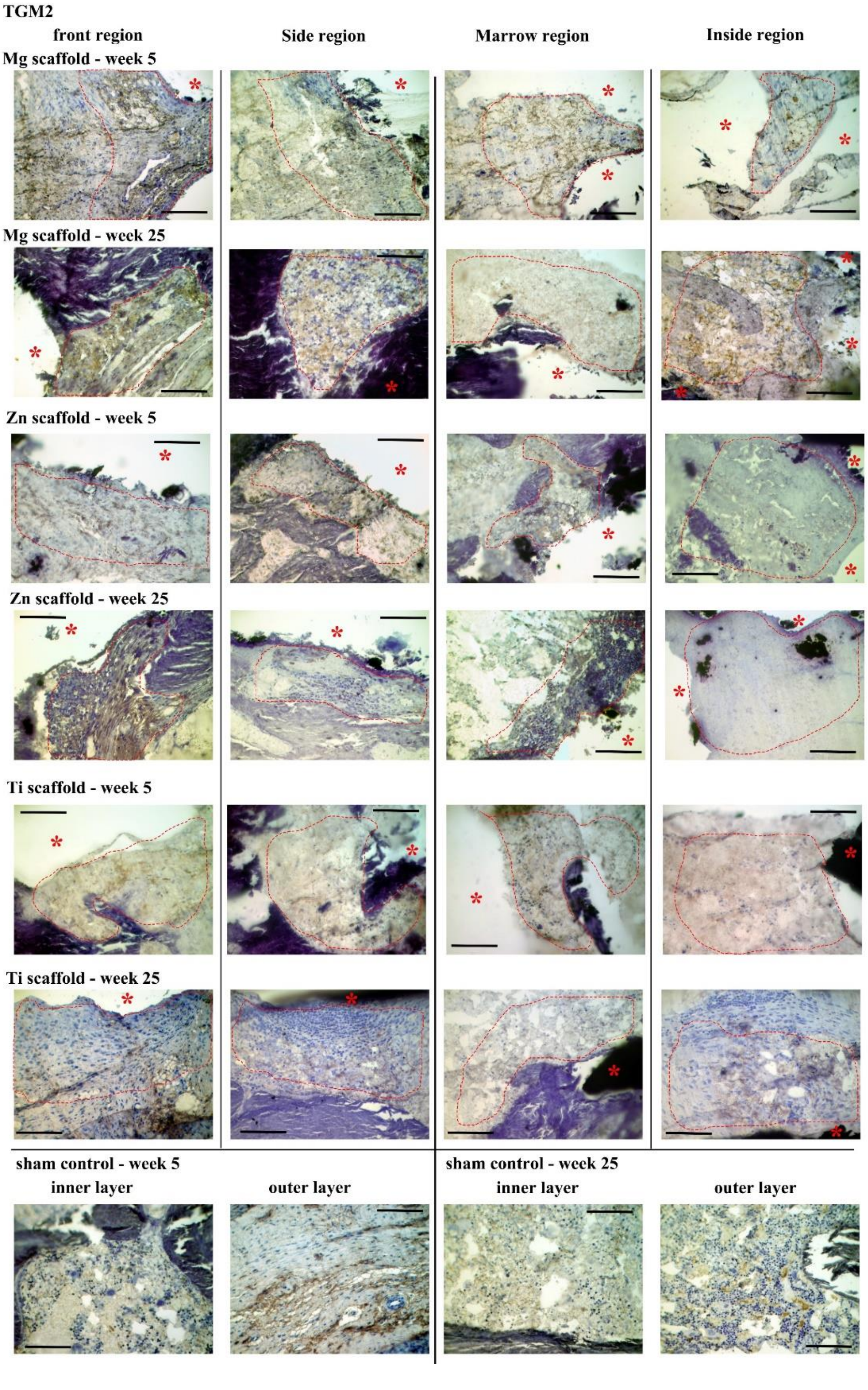

Fig. S22. IHC images of transglutaminase 2 (TGM2). Representative images of TGM2 marker expression from Mg-5w, Mg-25w, Zn-5w, Zn-25w, Ti-5w, Ti-25w, sham-5w, and sham-25w groups.

Images were captured from the front, side, marrow, and inside regions of the scaffold, and the inner and outer bone layers for the sham control groups. The location of the scaffold is indicated by *. The red dotted line represents the region of interest (ROI) for marker expression quantification. Black scale bar: 100 μm.

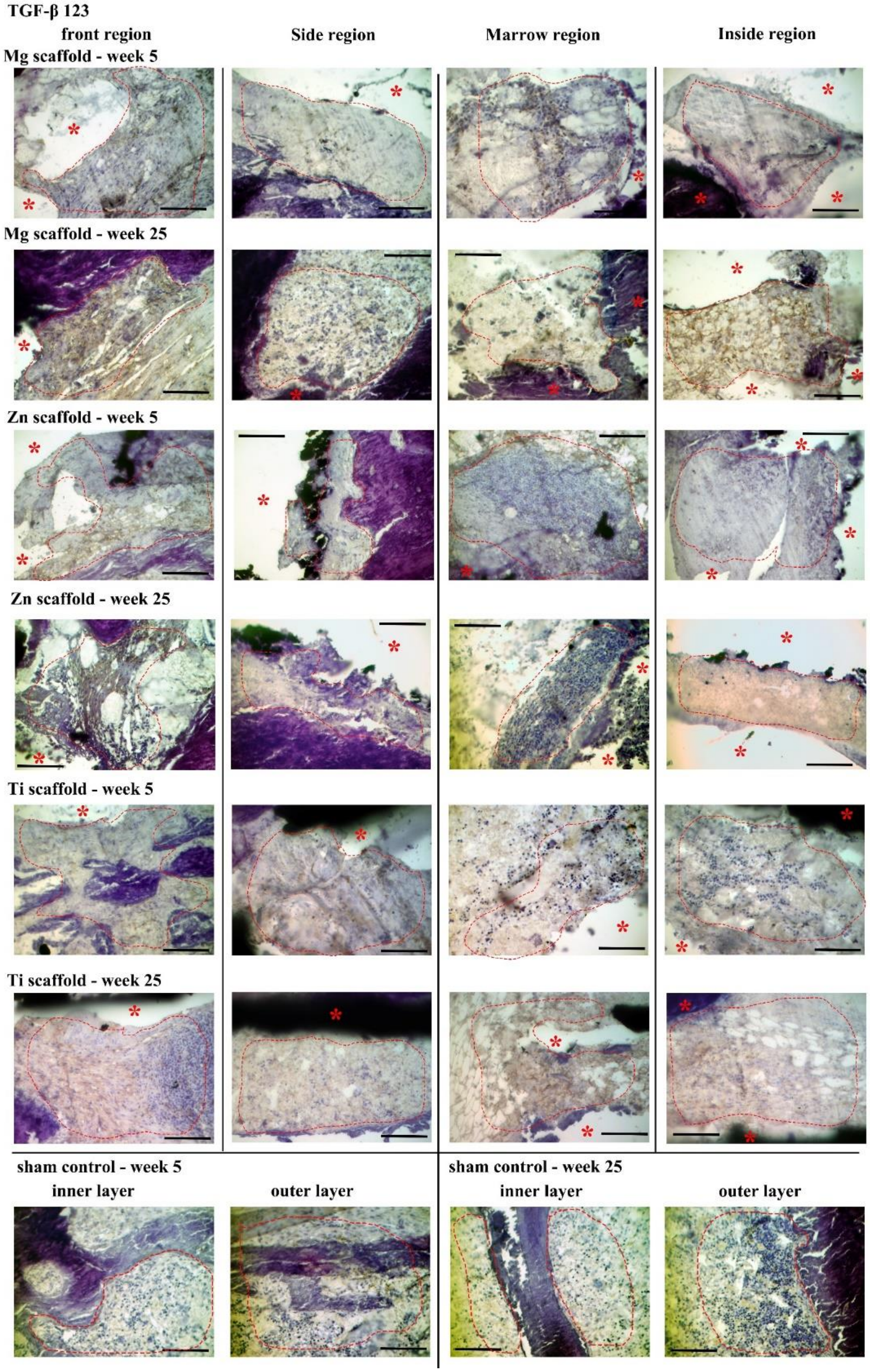

Fig. S23. IHC images of Transforming growth factor-beta-1,2,3 (TGF-β 123). Representative images of TGF-β 123 marker expression from Mg-5w, Mg-25w, Zn-5w, Zn-25w, Ti-5w, Ti-25w, sham-5w, and sham-25w groups. Images were captured from the front, side, marrow, and inside regions of the scaffold, and the inner and outer bone layers for the sham control groups. The location of the scaffold is indicated by *. The red dotted line represents the region of interest (ROI) for marker expression quantification. Black scale bar: 100 μm.

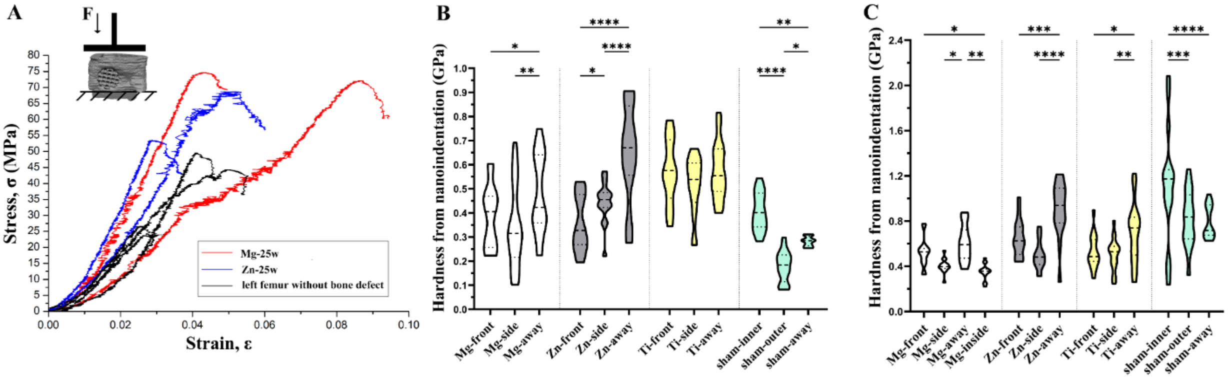

Fig. S24. Additional biomechanical tests. (A) Stress-strain curves obtained from compression tests on the bone tissue sample with Mg-25w, Zn-25w, and the left femur without bone defect. The inserted graph illustrates the configuration of compression tests. Hardness derived from nanoindentation tests on the bone tissue sample after implantation for (B) 5 weeks and (C) 25 weeks.

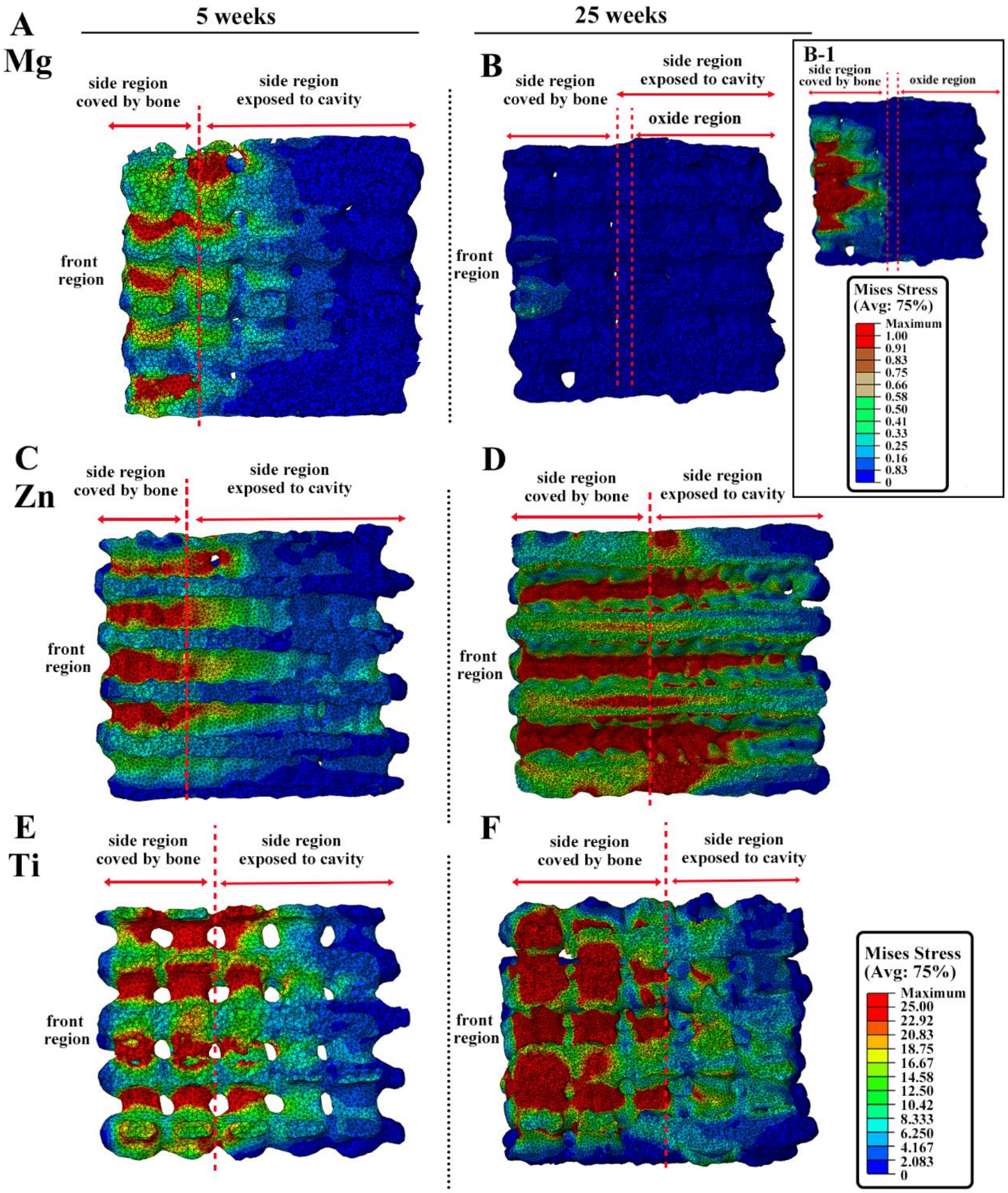

Fig.S25. Von Mises stress distribution on scaffolds. The von Mises stress distribution of Mg-5w (A), Mg-25w (B), Zn-5w (C), Zn-25w (D), Ti-5w (E), and Ti-25w (F) scaffolds on the cross-section of the longitudinal plane. The maximum limitation is set to 25 Mpa. In the Mg-25w group, the maximum limit is set to 1 Mpa for better visualization of the stress distribution (B-1). The red line indicates the scaffold within the front region. For the Mg-25w group, the oxide region is also labeled.

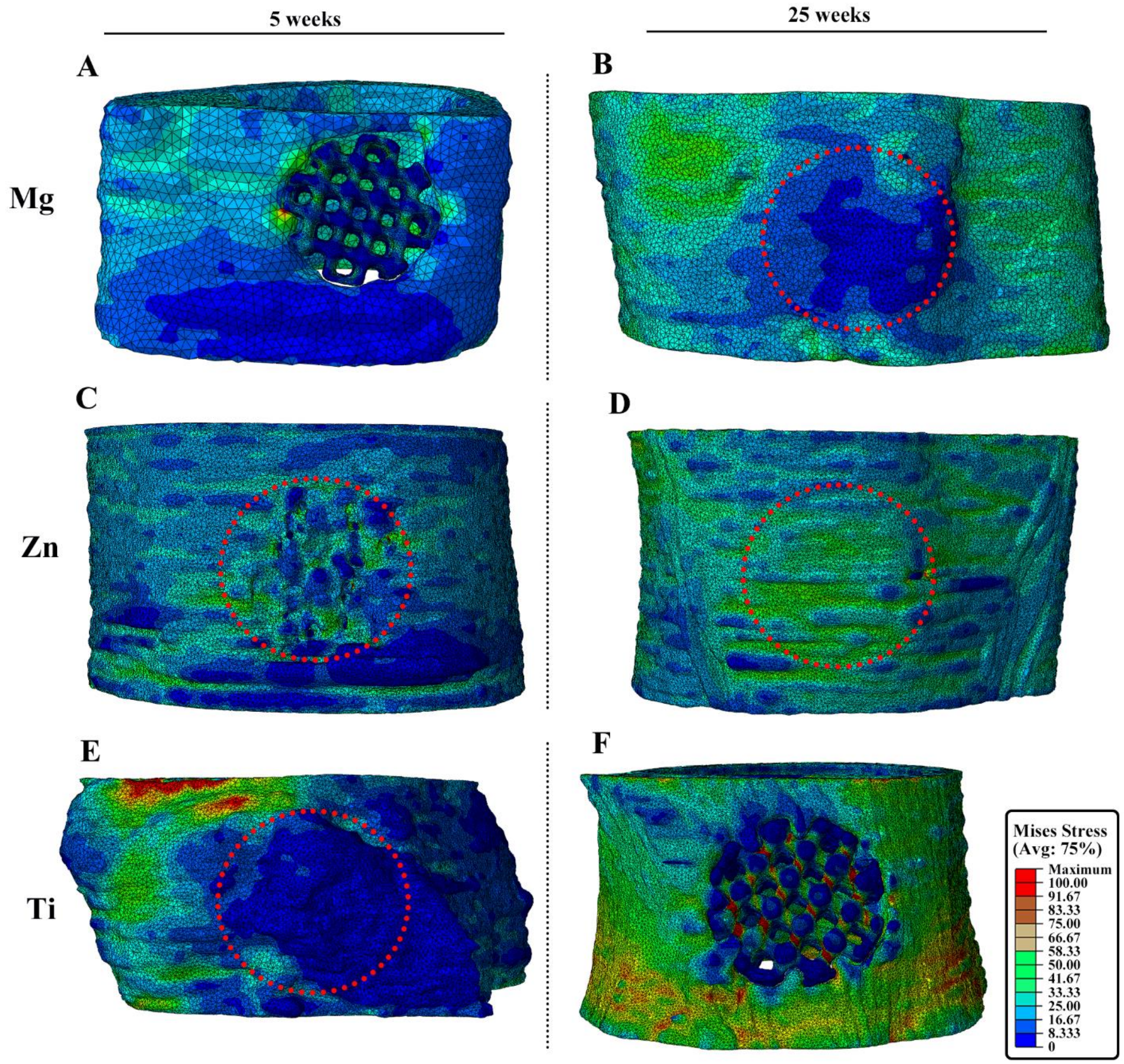

Fig.S26. Von Mises stress distribution on the front bone. The von Mises stress distribution on the front view of Mg-5w (A), Mg-25w (B), Zn-5w (C), Zn-25w (D), Ti-5w (E), and Ti-25w (F) models is shown. The maximum limitation is set to 100 MPa. The scaffold region covered by bone is delineated by the red dotted line.

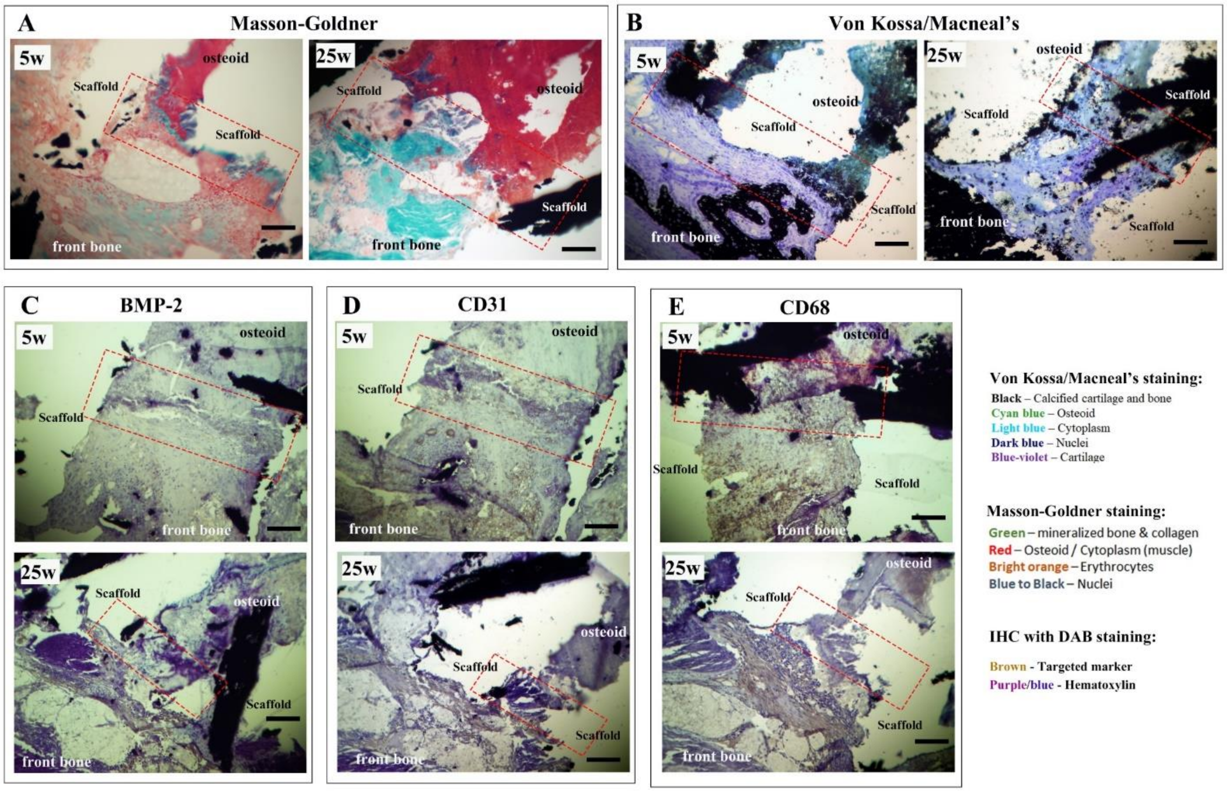

Fig. S27. Staining images showing the interface of osteoid in the front region of Zn scaffolds. Von Kossa/Macneal's staining (A) and Masson-Goldner staining (B) of Zn scaffold after 5- and 25-weeks implantation. Representative images of BMP-2 (C), CD31 (D), and CD68 (E) in the interface between the front and inside region of the Zn scaffold assessed by immunohistochemistry (IHC) with DAB staining. The red dotted box indicates the interface.

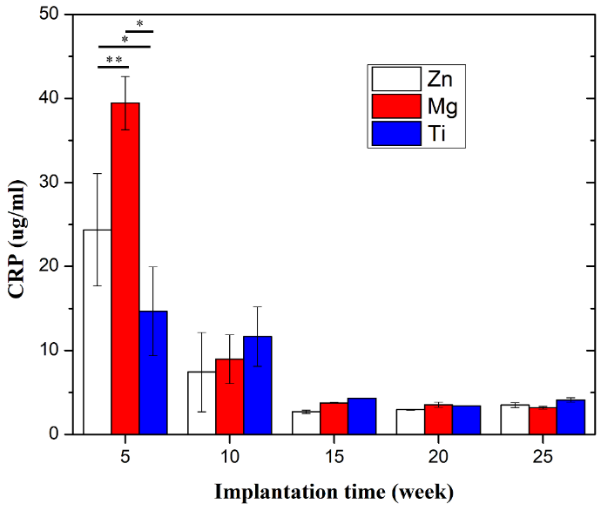

Fig. S28. CRP level in serum. Polished caption: The C-reactive protein (CRP) level in rabbit serum was measured at different time points for the Zn, Mg, and Ti scaffold groups.

Species	Strain /Breed	Sex	Date Collected
rabbit	NZW	F	8/11/2021

	Zn	Ti	Mg
ALP (U/L)	55	26	30
AST (U/L)	5	6	7
ALT (U/L)	11	14	11
Creatine kinase (U/L)	115	249	206
Albumin (g/dL)	4.1	4.2	4.3
Total Bilirubin (mg/dL)	0.1	0.1	0.1
Total Protein (g/dL)	5.6	6.1	6.3
Globulin (g/dL)	1.5	1.9	2.0
Bilirubin - Conjugated (mg/dL)	0.0	0.0	0.0
BUN (mg/dL)	18	16	14
Creatinine (mg/dL)	0.9	1.2	1.2
Cholesterol (mg/dL)	21	33	46
Glucose (mg/dL)	137	132	125
Calcium (mg/dL)	13.9[1]	7.2	14.5
Phosphorus (mg/dL)	4.0	3.9	3.5
Bicarbonate TCO2 (mmol/L)	21	19	22
Chloride (mmol/L)	100	100	101
Potassium (mmol/L)	4.3	9.0	5.0
ALB/GLOB ratio	2.7	2.2	2.2
Sodium (mmol/L)	140	144	146
BUN/Creatinine Ratio	20.0	13.3	11.7
Bilirubin - Unconjugated (mg/dL)	0.1	0.1	0.1
NA/K Ratio	33	16	29
Hemolysis Index	Normal	Normal	Normal
Lipemia Index	Normal	Normal	Normal

FootNotes

Index	Text
1	Results verified by repeat analysis.

Table S1. Blood chemistry panel of serum. Blood chemistry panel of serum samples collected from rabbits with 25-week implantation of Zn/Ti/Mg scaffold.

Marker expression in peri-implant region (within 200-300 um)							
A (positive area%)	0	<5%	5-10%	10-25%	25-50%	>50%	Final score
score	0	1	2	3	4	5	A x B (0-10)
B (intensity)	no expression	weak	strong				
score	0	1	2				

Colors assigned to the final score

0	1	2	3	4	6	8	10

Table S2. Scoring system used for semi-quantification IHC evaluation. The Immunoreactive Score (IRS) was calculated by multiplying the marker-positive area ratio (Score A: 0-5) with the staining intensity score (Score B: 0-2) in the peri-implant region within 200-300 μm. A rating mapping was established using different colors assigned to the final score (A x B: 0-10).

peri-implant tissue (within 200-300 um)						
apositive rea(%)	0	<5%	5-10%	10-25%	25-50%	> 50%
score A	0	1	2	3	4	5
expression intensity	negtive	weak	strong			
score B	0	1	2			
Final score: A x B (0-10)						

groups	Mg -5w				Mg -25w				Zn-5w				Zn-25w				Ti-5w				Ti-25w				sham-5w		sham-25w	
regions	front	side	marrow	inside	front	side	marrow	inside	front	side	marrow	inside	front	side	marrow	inside	front	side	marrow	inside	front	side	marrow	inside	front	side	marrow	inside
**BMP2**	6.6	5.8	5.7	9.4	16.2	3.3	4.5	6.4	19.5	10.5	8.2	0	14.8	25.5	8.0	0	6.0	6.3	1.7	2.4	8.5	14.2	4.1	5.2	9.6	10.9	3.2	6.1
	5.9	4.8	6.6	9.7	13.5	1.8	3.8	7.0	13.6	7.2	8.2		24.6	13.4	14.2		8.6	7.9	4.1	4.3	9.7	10.2	4.0	3.2	7.8	23.4	3.7	9.8
	6.3	4.0	9.6	13.4	13.8	1.3	5.5	8.4	16.7	6.1	8.4		16.3	10.2	7.5		7.6	7.1	2.3	4.4	8.7	10.0	7.0	9.7	7.1	15.4	2.5	5.9
	5.2	6.6	7.5	7.8	15.6	1.4	5.7	7.5	12.4	8.5	8.5		20.2	12.2	14.5		5.7	3.7	3.6	2.9	9.3	17.2	4.2	11.1	5.5	19.6	4.0	6.4
	6.6	8.7	12.9	4.8	7.3	2.9	3.4	5.0	16.8	11.4	9.0		16.1	14.4	9.1		6.8	3.1	2.0	2.9	7.6	13.4	2.6	4.5	8.6	13.6	5.4	9.3
average	6.1	6.0	8.5	9.0	13.3	2.2	4.6	6.9	15.8	8.7	8.4	0	18.4	15.2	10.7	0	6.9	5.6	2.7	3.4	8.8	13.0	4.4	6.7	7.7	16.6	3.8	7.5
SD	0.5	1.6	2.6	2.8	3.2	0.8	0.9	1.2	2.5	2.0	0.3	0	3.6	5.4	3.0	0	1.1	1.9	0.9	0.8	0.7	2.7	1.5	3.1	1.4	4.4	1.0	1.7
A score	2	2	2	2	3	1	1	2	3	2	2	0	3	3	3	0	2	2	1	1	2	3	1	2	2	3	1	2
B score	1	1	1	1	2	1	1	1	2	1	1	0	2	1	1	0	1	1	1	1	2	2	1	2	2	2	1	1
**final**	**2**	**2**	**2**	**2**	**6**	**1**	**1**	**2**	**6**	**2**	**2**	**0**	**6**	**3**	**3**	**0**	**2**	**2**	**1**	**1**	**4**	**6**	**1**	**4**	**4**	**6**	**1**	**2**
**Collagen I**	12.4	6.0	4.3	7.8	8.2	3.5	3.9	15.1	22.9	5.6	13.4	5.8	3.8	7.0	6.3	0	13.6	2.3	6.6	4.0	9.6	5.9	8.0	11.2	7.5	21.3	11.1	7.7
	17.1	9.1	4.1	4.4	8.6	7.0	4.1	9.6	12.0	3.9	9.0	8.0	4.9	4.3	8.7		13.6	3.4	2.7	5.1	7.3	3.6	3.1	10.8	7.4	15.0	8.6	10.6
	13.0	7.7	6.8	6.4	4.4	4.1	3.6	13.7	10.2	7.5	12.4	9.7	4.2	3.7	3.3		11.4	4.2	3.7	8.6	9.4	2.5	2.3	7.2	8.0	13.4	8.6	7.8
	15.0	8.0	3.6	6.2	7.3	3.1	1.7	16.0	11.5	7.8	9.4	4.4	2.2	3.1	3.4		13.6	1.8	3.4	9.3	8.6	3.4	3.3	9.4	9.3	13.4	7.3	9.2
	10.5	5.6	4.4	3.9	5.6	2.7	5.4	16.5	12.8	5.2	11.3	9.1	4.0	6.3	6.9		9.5	3.2	4.7	7.3	5.9	3.7	1.6	7.0	6.3	14.8	9.9	9.1
average	13.6	7.3	4.6	5.7	6.8	4.1	3.7	14.2	13.9	6.0	11.1	7.4	3.8	4.9	5.7	0	12.3	3.0	4.2	6.9	8.2	3.8	3.7	9.1	7.7	15.6	9.1	8.9
SD	2.3	1.3	1.1	1.4	1.6	1.5	1.2	2.5	4.6	1.5	1.7	2.0	0.9	1.5	2.1	0	1.6	0.8	1.4	2.0	1.4	1.1	2.2	1.8	1.0	2.9	1.3	1.1
A score	3	2	1	2	2	1	1	3	3	2	3	2	1	1	2	0	3	1	1	2	2	1	1	2	2	3	2	2
B score	2	1	1	1	1	1	1	1	2	1	2	1	1	1	1	0	2	1	1	1	1	1	1	2	2	2	1	1
**final**	**6**	**2**	**1**	**2**	**2**	**1**	**1**	**3**	**6**	**2**	**6**	**2**	**1**	**1**	**2**	**0**	**6**	**1**	**1**	**2**	**2**	**1**	**1**	**4**	**4**	**6**	**2**	**2**
**osteocalcin**	22.7	3.5	12.8	15.7	6.9	2.9	3.6	8.2	30.8	3.4	9.4	0	25.8	4.7	9.4	0	22.3	6.2	21.4	10.3	3.9	6.2	3.2	6.4	4.0	14.8	6.1	14.1
	27.2	1.5	9.1	14.6	2.7	2.4	4.0	6.5	31.3	3.3	9.1		15.1	6.3	7.2		29.8	7.7	22.7	11.3	2.9	2.2	6.7	12.8	3.7	22.3	3.8	7.6
	36.9	2.7	15.5	13.1	2.6	1.4	1.3	11.9	30.0	11.7	12.7		19.7	8.3	6.7		28.0	3.6	18.9	5.0	5.0	7.6	6.2	16.3	8.2	21.5	5.1	13.2
	42.8	4.9	11.1	13.7	5.5	0.7	5.6	8.6	23.5	8.4	13.3		22.1	3.0	10.2		23.2	5.6	16.5	6.0	9.4	2.2	7.6	16.5	6.2	28.8	9.4	11.3
	25.2	1.0	14.3	11.5	5.0	1.8	2.4	8.2	26.5	10.6	11.7		21.2	5.7	7.9		24.8	5.8	20.1	9.9	5.7	3.2	5.8	13.2	2.0	22.9	8.8	14.3
average	31.0	2.7	12.6	13.7	4.5	1.8	3.4	8.7	28.4	7.5	11.2	0	20.8	5.6	8.3	0	25.6	5.8	19.9	8.5	5.4	4.3	5.9	13.0	4.8	22.1	6.6	12.1
SD	7.6	1.4	2.3	1.4	1.7	0.8	1.4	1.8	3.0	3.5	1.7	0	3.5	1.8	1.3	0	2.9	1.3	2.1	2.5	2.2	2.2	1.5	3.7	2.2	4.5	2.1	2.5
A score	4	1	3	3	1	1	1	2	4	2	3	0	3	2	2	0	4	2	3	2	2	1	2	3	1	3	2	3
B score	2	1	2	2	1	1	1	1	2	1	2	0	1	1	1	0	2	2	2	1	1	2	2	2	1	1	1	1
**final**	**8**	**1**	**6**	**6**	**1**	**1**	**1**	**2**	**8**	**2**	**6**	**0**	**3**	**2**	**2**	**0**	**8**	**4**	**6**	**2**	**2**	**2**	**4**	**6**	**1**	**3**	**2**	**3**
**OPG**	33.3	17.5	6.4	6.4	36.2	6.7	17.7	24.6	19.3	7.7	14.8	12.3	27.5	16.4	17.8	0	12.5	14.8	10.2	12.3	11.9	9.4	8.9	10.2	8.2	10.4	8.7	13.2
	27.7	18.4	13.2	7.4	22.4	6.2	16.9	31.3	11.5	6.6	28.3	13.2	29.6	17.4	20.3		8.8	8.9	10.3	14.6	11.5	6.3	8.4	7.8	7.9	10.1	7.8	12.0
	21.5	12.1	15.8	11.5	23.8	4.2	11.4	33.1	17.8	11.7	17.6	4.5	21.4	21.7	26.1		9.4	9.2	8.2	9.7	4.7	8.4	13.1	11.5	10.1	11.7	4.6	9.3
	25.1	10.9	14.1	9.2	22.5	14.4	13.2	33.5	29.1	8.1	12.3	2.2	24.6	11.8	21.0		14.1	13.6	5.0	11.7	10.4	9.8	9.9	9.3	4.9	14.1	7.2	10.3
	21.7	12.2	14.3	10.0	24.6	8.0	15.6	31.2	23.9	14.5	16.3	7.2	25.5	15.3	18.7		8.0	7.3	12.2	12.7	8.1	14.2	8.6	10.0	7.2	14.2	7.7	13.8
average	25.9	14.2	12.8	8.9	25.9	7.9	15.0	30.7	20.3	9.7	17.9	7.9	25.7	16.5	20.8	0	10.6	10.8	9.2	12.2	9.3	9.6	9.8	9.8	7.7	12.1	7.2	11.7
SD	4.4	3.1	3.3	1.8	5.2	3.5	2.3	3.2	5.9	2.9	5.5	4.3	2.8	3.2	2.9	0	2.3	2.9	2.4	1.6	2.7	2.6	1.7	1.2	1.7	1.8	1.4	1.7
A score	4	3	3	2	4	2	3	4	3	2	3	2	4	3	3	0	3	3	2	3	2	2	2	2	2	3	2	3
B score	2	2	2	1	2	1	1	2	2	1	2	1	2	2	2	0	2	2	2	2	2	2	1	2	2	2	1	2
**final**	**8**	**6**	**6**	**2**	**8**	**2**	**3**	**8**	**6**	**2**	**6**	**2**	**8**	**6**	**6**	**0**	**6**	**6**	**4**	**6**	**4**	**4**	**2**	**4**	**4**	**6**	**2**	**6**
**RANKL**	9.4	7.0	4.0	4.1	6.8	0	6.2	4.5	15.8	20.1	25.4	0	13.8	21.0	5.0	0	14.5	5.7	6.3	12.4	10.5	12.5	2.1	6.7	14.1	12.0	1.8	4.9
	8.9	4.1	8.1	3.7	11.6		5.3	4.4	13.0	13.3	22.7		13.7	20.5	1.3		14.2	5.2	13.6	13.5	7.3	10.2	6.5	7.6	9.4	12.2	4.8	5.2
	6.8	5.2	7.7	4.2	9.4		6.1	6.0	10.8	15.8	35.3		15.7	15.1	3.8		18.8	4.6	6.0	8.4	5.7	10.1	3.5	5.4	12.8	8.0	4.7	7.3
	8.9	5.6	7.1	8.4	11.3		4.4	7.8	11.6	19.0	24.5		6.7	22.2	6.4		16.7	1.8	11.1	10.5	7.2	9.3	1.2	6.4	14.0	10.1	6.5	4.8
	11.6	5.5	4.9	3.1	6.6		4.8	5.7	15.2	16.3	23.4		8.5	11.9	10.7		9.2	4.2	11.0	13.8	5.1	16.4	4.2	8.2	7.4	12.1	3.7	5.5
average	9.1	5.5	6.4	4.7	9.1	0	5.4	5.7	13.3	16.9	26.3	0	11.7	18.1	5.4	0	14.7	4.3	9.6	11.7	7.2	11.7	3.5	6.9	11.5	10.9	4.3	5.5
SD	1.5	0.9	1.6	1.9	2.1	0	0.7	1.2	2.0	2.4	4.6	0	3.5	4.0	3.1	0	3.2	1.4	3.0	2.0	1.9	2.6	1.8	1.0	2.7	1.6	1.5	0.9
A score	2	2	2	1	2	0	2	2	3	3	4	0	3	3	2	0	3	1	2	3	2	3	1	2	3	3	1	2
B score	1	1	1	1	1	0	1	1	2	2	2	0	1	2	1	0	2	1	2	2	2	2	1	1	2	2	1	1
**final**	**2**	**2**	**2**	**1**	**2**	**0**	**2**	**2**	**6**	**6**	**8**	**0**	**3**	**6**	**2**	**0**	**6**	**1**	**4**	**6**	**4**	**6**	**1**	**2**	**6**	**6**	**1**	**2**
**ACP5**	0	0	0	0	3.8	0	0	8.2	11.3	3.8	5.8	0	7.1	0	0	0	0	0	0	0	0	0	0	0	0	0	0	8.5
					2.6		0	16.6	12.0	5.3	6.5		5.3															7.9
					2.4		0	8.5	8.2	7.3	7.6		7.3															8.0
					2.5		0	9.2	17.3	5.1	9.8		7.2															5.1
					2.1		0	7.4	10.5	6.0	10.5		7.4															4.8
average	0	0	0	0	2.7	0	0	10.0	11.9	5.5	8.0	0	6.8	0	0	0	0	0	0	0	0	0	0	0	0	0	0	6.9
SD	0	0	0	0	0.6	0	0	3.3	3.0	1.2	1.8	0	0.8	0	0	0	0	0	0	0	0	0	0	0	0	0	0	1.6
A score	0	0	0	0	1	0	0	2	3	2	2	0	2	0	0	0	0	0	0	0	0	0	0	0	0	0	0	2
B score	0	0	0	0	1	0	0	1	2	2	2	0	1	0	0	0	0	0	0	0	0	0	0	0	0	0	0	1
**final**	**0**	**0**	**0**	**0**	**1**	**0**	**0**	**2**	**6**	**4**	**4**	**0**	**2**	**0**	**0**	**0**	**0**	**0**	**0**	**0**	**0**	**0**	**0**	**0**	**0**	**0**	**0**	**2**
**CD31**	9.2	7.0	9.7	2.9	15.8	1.0	7.5	5.9	11.6	2.5	13.4	1.8	7.0	2.8	6.1	0	10.9	2.6	8.7	1.8	9.2	5.3	4.8	3.9	6.9	8.7	7.1	11.1
	11.5	8.9	4.7	2.8	14.8	0.4	5.7	6.2	8.6	8.2	8.2	4.4	8.1	2.2	13.0		19.6	3.3	8.5	3.8	8.4	6.3	2.6	3.8	5.1	14.1	5.4	6.1
	11.0	6.4	5.7	3.2	20.8	3.1	3.2	4.7	5.4	4.6	10.6	3.5	11.0	5.3	18.3		9.6	4.0	9.7	6.1	6.5	7.1	2.1	5.0	5.2	8.3	6.7	5.4
	11.5	4.4	7.8	5.3	7.5	2.4	2.8	2.8	10.4	6.4	9.5	7.1	5.1	1.6	11.4		10.7	6.6	7.3	4.2	10.5	4.1	3.7	4.4	7.7	14.4	4.8	9.3
	11.1	7.5	5.9	1.9	9.5	2.9	2.9	2.4	8.5	4.8	9.7	2.2	4.1	7.1	19.6		13.7	2.0	3.6	4.2	7.2	8.2	2.5	4.2	5.0	14.7	3.5	5.2
average	10.9	6.8	6.8	3.2	13.7	2.0	4.4	4.4	8.9	5.3	10.3	3.8	7.0	3.8	13.7	0	12.9	3.7	7.6	4.0	8.4	6.2	3.1	4.3	6.0	12.0	5.5	7.4
SD	0.8	1.5	1.8	1.1	4.7	1.1	1.9	1.6	2.1	1.9	1.7	1.9	2.4	2.1	4.9	0	3.6	1.6	2.1	1.4	1.4	1.4	1.0	0.4	1.1	2.9	1.3	2.4
A score	3	2	2	1	3	1	1	1	2	2	3	1	2	1	3	0	3	1	2	1	2	2	1	1	2	3	2	2
B score	2	1	1	1	2	1	1	2	2	2	2	1	2	1	2	0	2	1	1	1	2	2	1	1	2	2	1	2
**final**	**6**	**2**	**2**	**1**	**6**	**1**	**1**	**2**	**4**	**4**	**6**	**1**	**4**	**1**	**6**	**0**	**6**	**1**	**2**	**1**	**4**	**4**	**1**	**1**	**4**	**6**	**2**	**4**
**VEGF**	23.8	19.7	8.1	8.7	8.9	6.4	3.2	13.9	16.9	12.2	19.1	0.0	10.7	7.7	16.3	0	17.5	9.4	3.5	10.2	14.8	16.4	11.4	7.4	12.1	18.1	7.4	12.8
	19.0	20.6	12.1	5.9	9.7	12.1	2.8	10.5	17.9	14.1	27.0	7.6	10.2	6.0	16.1		20.2	7.1	2.2	12.1	14.8	13.9	8.3	5.4	10.9	22.3	10.0	17.3
	25.9	19.3	13.3	4.4	10.4	4.9	2.9	16.5	21.8	8.7	19.8	2.8	13.5	11.0	17.2		23.5	9.4	5.0	8.9	10.4	19.0	13.3	7.0	12.2	17.3	7.2	12.3
	28.0	24.1	14.8	6.7	8.6	9.5	1.6	12.7	18.5	7.3	18.3	7.5	10.4	5.5	14.9		22.9	9.6	6.7	11.5	12.5	18.5	8.0	6.9	7.8	18.9	8.3	12.9
	23.8	17.2	18.8	4.0	9.6	6.6	5.3	12.8	21.7	11.4	15.9	4.5	11.2	6.5	23.1		21.7	9.0	6.3	12.2	12.2	18.2	6.3	7.4	12.2	18.6	6.6	19.0
average	24.1	20.2	13.4	5.9	9.5	7.9	3.2	13.3	19.4	10.7	20.0	4.5	11.2	7.3	17.5	0	21.1	8.9	4.7	11.0	12.9	17.2	9.5	6.8	11.0	19.1	7.9	14.8
SD	3.0	2.2	3.5	1.7	0.6	2.6	1.2	1.9	2.0	2.5	3.7	2.9	1.2	2.0	2.9	0	2.2	0.9	1.7	1.3	1.7	1.9	2.5	0.8	1.7	1.7	1.2	2.8
A score	3	3	3	2	2	2	1	3	3	3	3	2	3	2	3	0	3	2	1	3	3	3	2	2	3	3	2	3
B score	2	2	1	1	2	1	1	2	2	1	2	1	1	1	2	0	2	1	1	1	2	2	1	1	2	2	1	1
**final**	**6**	**6**	**3**	**2**	**4**	**2**	**1**	**6**	**6**	**3**	**6**	**2**	**3**	**2**	**6**	**0**	**6**	**2**	**1**	**3**	**6**	**6**	**2**	**2**	**6**	**6**	**2**	**3**
**CD68**	3.8	1.7	13.3	6.9	16.9	9.1	4.4	20.3	27.8	10.2	27.5	0	7.3	9.5	50.2	0	21.6	18.1	34.3	18.5	1.8	3.4	2.7	4.3	6.3	26.8	2.9	9.1
	4.0	4.5	14.3	3.3	9.1	8.1	6.8	12.9	23.6	13.0	33.7		8.4	20.0	46.0		29.6	14.1	40.9	25.4	4.9	3.3	5.5	5.5	4.1	17.3	4.3	13.9
	3.6	4.5	14.6	6.3	19.7	8.7	8.7	13.4	23.3	10.2	38.5		10.1	13.0	33.2		20.4	22.8	37.5	22.6	4.3	5.9	5.1	1.8	5.4	25.0	4.2	12.9
	1.7	1.6	8.4	2.3	23.5	5.5	8.9	9.2	27.1	11.6	21.6		7.9	12.1	54.8		32.7	21.4	39.3	19.8	3.4	4.0	2.3	3.9	3.1	16.9	3.2	9.2
	2.3	1.2	12.1	4.5	9.0	6.8	3.2	13.1	26.1	12.3	28.5		8.9	14.1	44.2		23.5	18.5	35.4	20.9	3.7	4.3	3.0	1.2	6.5	16.5	3.9	11.5
average	3.1	2.7	12.5	4.7	15.6	7.6	6.4	13.8	25.6	11.5	30.0	0	8.5	13.7	45.7	0	25.6	19.0	37.5	21.4	3.6	4.2	3.7	3.4	5.1	20.5	3.7	11.3
SD	0.9	1.5	2.2	1.7	5.8	1.3	2.3	3.6	1.8	1.1	5.7	0	1.0	3.5	7.2	0	4.8	3.0	2.4	2.4	1.0	0.9	1.3	1.6	1.3	4.5	0.6	1.9
A score	1	1	3	1	3	2	2	3	4	3	4	0	2	3	4	0	4	3	4	3	1	1	1	1	2	3	1	3
B score	1	1	1	1	2	1	1	1	2	1	2	0	1	1	2	0	2	2	2	1	1	1	1	1	1	2	1	2
**final**	**1**	**1**	**3**	**1**	**6**	**2**	**2**	**3**	**8**	**3**	**8**	**0**	**2**	**3**	**8**	**0**	**8**	**6**	**8**	**3**	**1**	**1**	**1**	**1**	**2**	**6**	**1**	**6**
**TNF-alpha**	4.5	7.4	6.0	0	19.4	5.0	6.4	15.7	1.4	3.4	29.3	0	12.5	3.0	39.1	0	18.0	8.3	14.7	15.8	21.5	4.6	5.0	6.4	0	3.0	0	10.1
	1.4	3.6	5.4		19.5	3.5	8.1	17.3	3.7	1.8	23.5		9.7	3.2	42.5		13.4	9.7	17.1	15.9	30.0	6.3	5.1	8.6		7.1		10.8
	3.6	4.4	7.3		13.9	3.8	11.1	14.1	6.2	2.9	26.7		9.7	2.2	23.9		12.4	7.3	13.7	10.4	20.6	3.8	6.7	6.1		2.6		11.3
	5.2	6.1	2.5		20.2	9.3	8.7	8.8	2.4	1.7	27.3		6.8	3.5	44.2		14.9	8.5	12.6	6.5	21.8	5.2	9.1	9.2		4.7		13.6
	3.8	5.5	5.0		23.0	6.5	4.4	13.5	3.8	2.1	27.2		9.5	5.8	39.2		14.2	8.2	11.2	10.2	16.7	4.8	6.7	10.8		4.1		12.7
average	3.7	5.4	5.2	0	19.2	5.6	7.7	13.9	3.5	2.4	26.8	0	9.6	3.5	37.8	0	14.6	8.4	13.9	11.8	22.1	4.9	6.5	8.2	0	4.3	0	11.7
SD	1.3	1.3	1.6	0	3.0	2.1	2.2	2.9	1.6	0.7	1.9	0	1.8	1.2	7.2	0	1.9	0.8	2.0	3.6	4.3	0.8	1.5	1.8	0	1.6	0	1.3
A score	1	2	2	0	3	2	2	3	1	1	4	0	2	1	4	0	3	2	3	3	3	1	2	2	0	1	0	3
B score	1	2	1	0	2	1	1	2	1	1	2	0	1	1	2	0	2	1	2	2	2	1	1	1	0	2	0	1
**final**	**1**	**4**	**2**	**0**	**6**	**2**	**2**	**6**	**1**	**1**	**8**	**0**	**2**	**1**	**8**	**0**	**6**	**2**	**6**	**6**	**6**	**1**	**2**	**2**	**0**	**2**	**0**	**3**
**TGM2**	24.0	20.9	14.9	9.8	34.5	17.2	5.1	24.5	7.2	10.7	28.3	0	52.7	6.6	42.2	0	6.5	5.5	12.0	3.5	13.5	15.8	4.3	12.9	5.3	20.4	5.2	10.2
	28.8	15.8	17.8	10.5	30.4	20.0	8.1	28.9	9.9	9.2	20.2		63.1	6.1	36.1		7.1	4.7	15.8	3.9	9.0	13.4	0.7	21.8	4.2	20.6	4.0	11.7
	36.5	17.8	13.3	8.7	22.2	12.8	6.4	18.0	4.7	7.4	13.3		56.3	9.7	50.2		6.3	6.5	9.5	7.1	8.5	9.5	2.6	17.8	6.7	19.6	6.5	12.4
	32.8	18.2	12.8	8.6	31.1	11.3	6.9	20.1	6.7	11.0	15.8		61.3	8.7	32.8		10.8	6.4	12.2	2.2	8.2	8.8	2.1	13.4	6.5	19.7	5.9	11.1
	27.4	18.8	13.8	8.5	25.2	12.6	7.1	16.7	7.3	9.8	11.4		52.5	7.9	24.4		2.5	8.3	15.5	4.2	8.1	15.4	2.5	15.8	6.3	15.8	7.8	12.9
average	29.9	18.3	14.5	9.2	28.7	14.8	6.7	21.6	7.2	9.6	17.8	0	57.2	7.8	37.1	0	6.6	6.3	13.0	4.2	9.5	12.6	2.4	16.3	5.8	19.2	5.9	11.7
SD	4.3	1.6	1.8	0.8	4.4	3.3	1.0	4.5	1.7	1.3	6.0	0	4.4	1.3	8.7	0	2.6	1.2	2.4	1.6	2.0	2.9	1.2	3.2	0.9	1.8	1.3	1.0
A score	4	3	3	2	4	3	2	3	2	2	3	0	5	2	4	0	2	2	3	1	2	3	1	3	2	3	2	3
B score	2	1	1	1	2	2	1	2	2	1	2	0	2	1	2	0	2	1	1	1	2	1	1	1	1	2	1	2
**final**	**8**	**3**	**3**	**2**	**8**	**6**	**2**	**6**	**4**	**2**	**6**	**0**	**10**	**2**	**8**	**0**	**4**	**2**	**3**	**1**	**4**	**3**	**1**	**3**	**2**	**6**	**2**	**6**
**TGF-β 123**	11.9	2.4	18.4	6.3	15.8	5.9	12.2	24.5	15.8	0	7.1	0	32.9	7.4	14.4	0	6.3	9.7	8.7	3.7	12.0	6.5	8.3	6.4	0	3.1	0	11.8
	15.3	4.6	13.1	6.0	16.1	8.4	7.5	29.0	10.1		10.9		41.4	8.3	18.1		10.4	16.8	12.4	2.7	10.8	2.4	15.4	4.7		7.5		13.8
	14.6	7.8	12.6	3.4	16.8	5.0	11.2	25.0	10.0		13.0		28.5	3.8	17.2		14.5	17.3	13.9	10.4	10.5	5.5	10.9	7.2		2.9		8.8
	9.4	5.2	9.2	3.4	15.6	11.4	16.0	15.9	14.2		15.6		21.2	4.1	12.5		11.9	11.3	14.8	2.8	6.6	10.7	10.3	6.3		3.7		9.1
	13.2	6.1	14.5	4.1	15.4	4.8	13.5	22.8	14.1		18.6		28.7	5.3	20.7		18.5	9.0	12.6	3.6	7.1	7.4	15.8	8.7		4.2		14.3
average	12.9	5.2	13.6	4.6	15.9	7.1	12.1	23.4	12.8	0	13.0	0	30.5	5.8	16.6	0	12.3	12.8	12.5	4.6	9.4	6.5	12.1	6.7	0	4.3	0	11.6
SD	2.1	1.8	3.0	1.3	0.5	2.5	2.8	4.3	2.4	0	3.9	0	6.6	1.8	2.9	0	4.1	3.5	2.1	2.9	2.1	2.7	3.0	1.3	0	1.7	0	2.3
A score	3	2	3	1	3	2	3	3	3	0	3	0	3	2	3	0	3	3	3	1	2	2	3	2	0	1	0	3
B score	2	1	2	1	2	1	1	2	2	0	2	0	2	1	2	0	2	2	1	1	2	1	1	1	0	1	0	2
**final**	**6**	**2**	**6**	**1**	**6**	**2**	**3**	**6**	**6**	**0**	**6**	**0**	**6**	**2**	**6**	**0**	**6**	**6**	**3**	**1**	**4**	**2**	**3**	**2**	**0**	**1**	**0**	**6**

Table S3. Summary of semi-quantification of IHC evaluation (Exile file). The expression of different markers in the front, side, marrow, and inside regions of each group. The region of interest (ROI) for evaluation is the peri-implant region within 200-300 µm. Evaluation of each region was performed on 5 images. The marker-positive area ratio (score A) was determined by using ImageJ Fiji (*47*). The expression intensity (score B) was observed and classified according to the following parameters: score 0 (absent expression), score 1 (weak expression), and score 2 (strong expression). The final score = A x B.
